# Strengthening mental health research outcomes through genuine partnerships with young people with lived or living experience: A pilot evaluation study

**DOI:** 10.1111/hex.13777

**Published:** 2023-05-17

**Authors:** Grace Yeeun Lee, Sarah McKenna, Yun Ju C. Song, Alexis Hutcheon, Samuel J. Hockey, Rachael Laidler, Jo‐An Occhipinti, Claudia Perry, Tara Lindsay‐Smith, Annabel Ramsay, Skye Choi, Dakota Feirer, Andrew W. Shim, Jessica Cottle, Anith Mukherjee, Joshua New, Rebecca Yu, Elizabeth Mary Scott, Louise Freebairn, Ian Bernard Hickie

**Affiliations:** ^1^ Brain and Mind Centre New South Wales Australia; ^2^ Computer Simulation & Advanced Research Technologies (CSART) Sydney New South Wales Australia; ^3^ National Centre for Epidemiology and Population Health The University of Canberra Canberra Australian Capital Territory Australia

**Keywords:** consumer partnership, empowerment evaluation, lived experience, nothing about us without us, participatory action research, researcher‐youth lived experience partnership, youth mental health

## Abstract

**Background:**

Despite increasing support for stakeholder inclusion in research, there is limited evaluative research to guide safe (i.e., youth‐friendly) and meaningful (i.e., non‐tokenistic) partnerships with young people with lived experience of mental ill‐health in research. This paper describes a pilot evaluation and iterative design of a Youth Lived Experience Working Group (LEWG) protocol that was established by the Youth Mental Health and Technology team at The University of Sydney's Brain and Mind Centre, based on the results of two studies.

**Methods:**

Study one consisted of a pilot evaluation of the extent to which youth partners felt empowered to contribute, to qualitatively explore how LEWG processes could be improved. Youth partners completed online surveys, and results were shared over two LEWG meetings in 2021 to empower youth partners to collectively identify actions of positive change regarding LEWG processes. These meetings were audio‐recorded and transcripts were subsequently coded using thematic analysis. Study two assessed whether LEWG processes and proposed improvements were acceptable and feasible from the perspective of academic researchers via an online survey in 2022.

**Results:**

Quantitative and qualitative data collected from nine youth partners and 42 academic researchers uncovered initial learnings regarding facilitators, motivators, and barriers to partnering with young people with lived experience in research. Implementing clear processes for youth partners and academic researchers on effective partnership strategies, providing training opportunities for youth partners to develop research skills, and providing regular updates on how youth partner contributions led to research outcomes were identified as key facilitators.

**Conclusions:**

This pilot study provides insight into a growing international field on how to optimise participatory processes so that researchers and young people with lived experience can be better supported and engaged to make meaningful contributions to mental health research. We argue that more transparency is needed around participatory research processes so that partnerships with young people with lived experience are not merely tokenistic.

**Consumer Contributions:**

Our study has also been approved by and reflects the concepts and priorities of our youth lived experience partners and lived experience researchers, all of whom are authors of this paper.

## 1 INTRODUCTION

Health researchers face persistent challenges translating key findings into real‐world settings, limiting the impact of their research and more critically, its ability to improve the health outcomes of patients.^1^ For this reason, leaders in health service delivery have endorsed the *Nothing About Us Without Us* principle^2^ to ensure that the values and preferences of key stakeholders, particularly people with lived experience, are incorporated in the design and implementation of programmes and services that impact their everyday lives, improving the translatability of health policies and research.^3^, ^4^ Participatory approaches may be particularly valuable in youth mental health research, where ‘youth’ may broadly be defined between 12 and 30 years old, as low rates of help‐seeking and high drop‐out rates indicate a strong impetus to improve the acceptability of evidence‐based treatments for this population.^4^, ^5^, ^6^, ^7^ Despite this, genuine partnerships with young people with lived experience of mental ill‐health continue to be limited in academic research.^6^, ^7^, ^8^, ^9^

Reviews of the field have continuously pointed to a lack of transparency from research teams regarding the processes and protocols that are used to engage consumers as research partners, coupled with limited evaluative research that can guide participatory research efforts.^10^, ^11^, ^12^ Given the unique challenges that exist when working with youth populations, particularly those with mental ill‐health, it is important that clear guidelines and protocols are provided for youth mental health research. Barriers include young people mistrusting researchers due to a belief that lived experience expertise will be devalued in favour of academic expertise, and power imbalances between academic staff and lived experience staff that are further heightened when academics are working with young people.^13^, ^14^, ^15^, ^16^ On the other hand, research partnerships can help young people to feel empowered and have positive benefits for their wellbeing.^10^, ^11^, ^12^, ^17^ Moreover, researchers report that these partnerships improve the quality and translatability of their research.^12^, ^18^, ^19^ It is therefore incumbent upon academics to provide greater transparency regarding participatory research processes to ensure that their methods are optimally designed to empower young people and to ensure that all types of knowledge, including ‘lived experience’ and ‘academic’, are equally valued.^9^, ^20^

### 1.1 The Brain and Mind Centre's Youth Lived Experience Working Group (LEWG)

#### 1.1.1 Overview and theoretical framework

The Youth Mental Health and Technology (YMH) team at The University of Sydney's Brain and Mind Centre established the LEWG in February 2021. This presented a valuable opportunity to design and evaluate a LEWG protocol (i.e., the standard operating procedure for involving young people with lived experience of mental ill‐health in projects conducted by the YMH team; see Supplementary Material 1). The LEWG aims to embed young people with lived and/or living experience in all aspects of the research cycle from the design through to dissemination across every research stream (or sub‐team) within the YMH team.^21^ The nature of LEWG involvement in research projects can involve consultation (e.g., academic researchers seeking feedback on various aspects of their project from youth partners during monthly meetings), partnership (e.g., academic researchers, lived experience researchers, and LEWG members co‐author academic outputs and co‐design LEWG protocols), and citizen control (e.g., LEWG members and lived experience researchers produce their own podcasts, webinars and articles).^22^ A pilot study was conducted to evaluate the LEWG's protocol against guidelines for lived experience partnerships from the perspectives of both young people and academic researchers ahead of a longitudinal evaluation study of the impact of the LEWG on the YMH team's research projects and outputs.

In designing our own protocol, we sought processes that aligned with existing frameworks for participatory involvement but also suited the needs and values of our group members. For example, whilst Arnstein's seminal ladder of participation conceptualises ‘citizen‐control’ as the highest rung of participation, young people often prefer to move dynamically between different levels of participation based on their levels of interest, experience, and comfort over time.^23^ Thus, according to Arunkumar and colleagues, it is important that researchers enable young people to choose and change their depth of involvement, increase the capacity for young people to be involved in decision‐making when problems arise, and ensure accountability between researchers and young people.^24^, ^25^ Likewise, Kirwan and colleagues have recommended that researchers follow ‘basic principles’ rather than prescriptive frameworks when designing participatory methods, given that the nature and goals of consumer research participation may greatly differ between research projects and real‐world settings.^26^ These principles include supportive institutional policies, supportive attitudes that ensure strong communication and shared goals, mutual respect, addressing training needs, providing resources and advanced planning, and recognising the value of research partnerships across all stages of research from conceptualisation through the dissemination of findings.^26^ In collaboration with our lived experience researchers, we sought to ensure that these principles recommended by Arunkumar et al. and Kirwan et al. were integrated into the planning and implementation of our LEWG.

#### 1.1.2 Terminology and roles

Young people who are members of the LEWG are referred to as *youth partners*. Staff members of the YMH team with lived experience of mental ill‐health are referred to as *lived experience researchers*. Though youth partners and lived experience researchers did not hold postgraduate qualifications, they are regarded as equal experts in their lived experience. Distinctions are made in this paper based on the types of knowledge and credentials held by each member of our team. Staff researchers within the YMH research team (including external collaborators) with academic credentials are referred to as *academic researchers*. None of our academic researchers declared having lived experience of mental ill‐health.

#### 1.1.3 Integrating youth partnerships during planning

**Figure 1:**
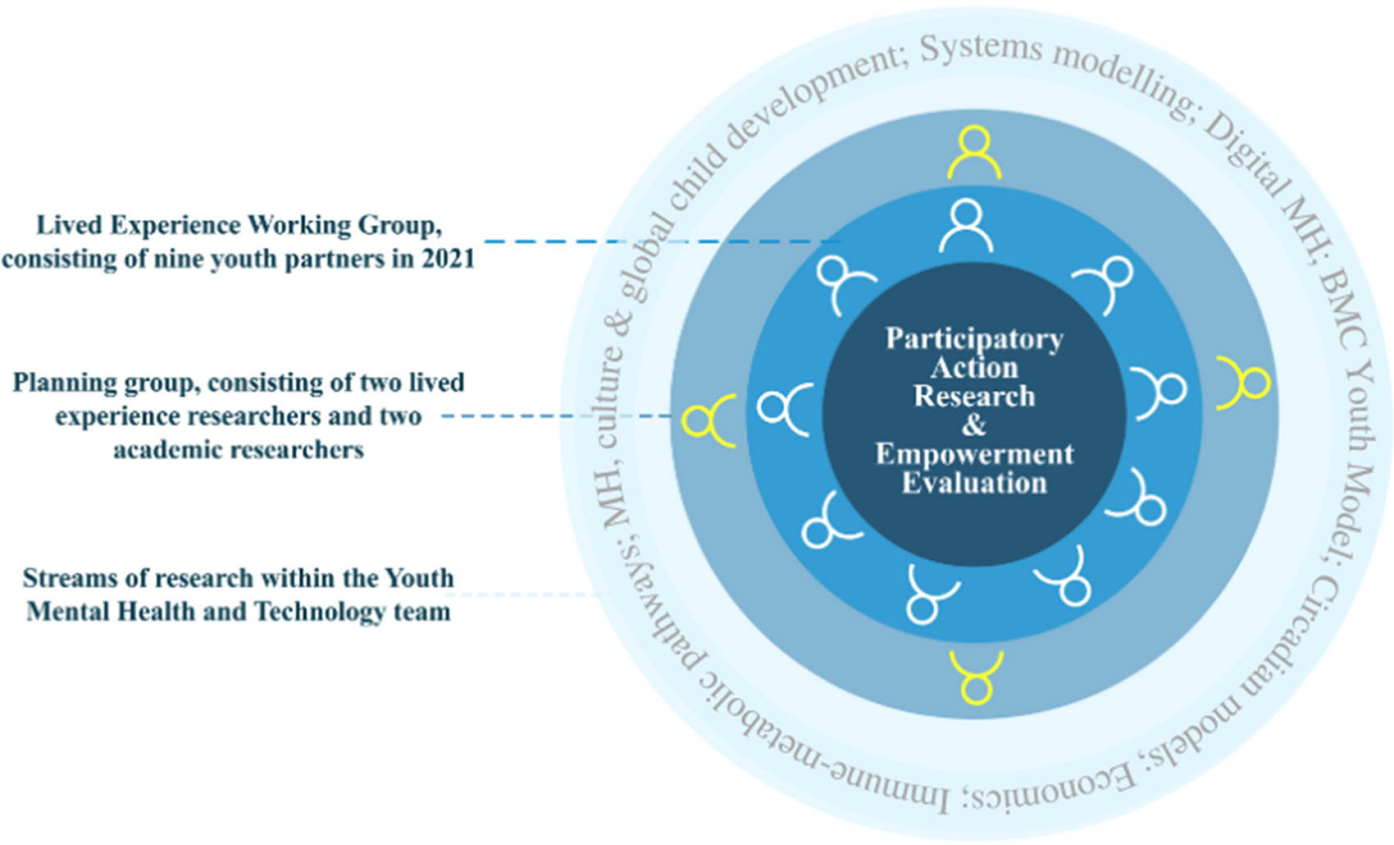
Youth Lived Experience Working Group (LEWG) model within the Brain and Mind Centre's Youth Mental Health and Technology (YMH) team. The LEWG aims to embed young people with lived experience in all aspects of the research cycle across each stream of research of the YMH team. Participatory action research and empowerment evaluation underpin the study design to enable and improve the self‐determination of young people with lived experience of mental ill‐health.

A unique lived experience partnership model was established that consisted of youth partners as part of the LEWG and a planning group (Figure 1). The planning group included two youth lived experience researchers (A. H., S. J. H.) and two academic researchers (G. Y. L., Y. J. C. S.). A. H. and S. J. H. are employed by the YMH team to ensure research projects are aligned with the values and perspectives of young people with living experience of mental ill‐health. As part of the planning group, they were involved in managing the LEWG, recruiting new members, contributing to ethics applications including the design of evaluation tools to ensure that they were acceptable to young people, and co‐authoring academic papers. G. Y. L and Y. J. C. S. were also involved in organising and running LEWG and provided academic mentorship regarding ethical research guidelines and existing recommendations from past research regarding frameworks of engagement.

#### 1.1.4 Overview of LEWG processes

The first LEWG meeting was held in April 2021, and the working group continues to meet monthly for at least three hours. Youth partners of the LEWG are reimbursed for their time in line with the National Paid Participation Policy ($52.25 AUD per hour)^27^ to foster a more equal working relationship.^28^, ^29^ Throughout the first year since establishing the LEWG, youth partners engaged with and provided monthly expert feedback to academic researchers from the YMH team, excluding external collaborators, who attended LEWG meetings to embed youth partners’ expertise in their research.

### 1.2 Objectives

Our objective was to refine the LEWG protocol based on the results of two studies. Study one consisted of a pilot evaluation of the extent to which youth partners felt empowered to contribute to research through the LEWG and to qualitatively explore how our processes could be improved. Study two assessed whether our processes and proposed improvements were acceptable and feasible from the perspective of academic researchers.

## 2 METHODS

### 2.1 Ethics and governance

The pilot evaluation study was approved by The University of Sydney's Human Research Ethics Committee (2020/786). Our study has also been approved by and reflects the concepts and priorities of our youth partners and lived experience researchers.

### 2.2 Recruitment

#### 2.2.1 Study #1

Passive sampling (e.g., researchers at an arms‐length) was adopted to recruit young people with lived and/or living experience of mental ill‐health to join the LEWG (16–30 years old) via online advertisements and referrals. Youth partners from the LEWG were invited to participate in a baseline survey. The results of this survey were then shared during two meetings. These meetings were audio‐recoded and qualitatively analysed.

#### 2.2.2 Study #2

Academic researchers (≥18 years old) from the YMH team, including external collaborators of the YMH team from various international universities were also invited to participate in a baseline survey. As snowball recruitment was adopted to recruit academic researchers, it is possible that both academic researchers (i.e., researchers without lived mental health experience) and lived experience researchers (i.e., researchers with lived mental health experience) completed the survey. Researchers who administered the survey (i.e., LEWG planning group) did not complete the survey.

To avoid any perceived coercion, all recruitment remained passive (e.g., via online advertisements) to ensure that youth partners and academic researchers had control over their decision to participate.

### 2.3 Design

This project was informed by principles of participatory action research and empowerment evaluation. Participatory action research seeks to involve stakeholders, in this case, lived experience researchers and youth partners, as both subjects and co‐researchers.^30^ This approach assumes that causal inferences made about research findings are likely to be more valid when they are informed with and by stakeholders most directly impacted by research outcomes.^30^ Empowerment evaluation approaches have a similar focus, but additionally seek to improve the self‐determination of these populations; in other words, to upskill stakeholders to make positive changes for their community through evaluation.^31^ As such, two youth lived experience researchers (A. H., S. J. H.) co‐developed the initial protocol before the establishment of the LEWG, which included designing evaluation tools and interpreting existing evidence and subsequently made joint decisions with youth partners to modify the LEWG protocol.^30^, ^31^

### 2.4 Data collection and analysis

Quantitative data was collected through online surveys administered to youth partners (study #1) and to academic researchers (study #2) via Qualtrics. Youth partners were asked to complete a baseline survey in July 2021, after the third LEWG meeting, to allow time to build trust and rapport between youth partners and the LEWG planning group. A baseline survey was circulated to all academic researchers, which included international research collaborators of the YMH team throughout February and March 2022 to understand the broader acceptability and feasibility of our protocol. Both surveys were adapted from previous surveys on lived experience inclusion in research that had been administered to either academic researchers or individuals with lived experience,^32^, ^33^, ^34^ and underwent further co‐design and cognitive testing with two lived experience researchers from the LEWG planning group to ensure survey questions could be easily understood and completed by respondents (Supplementary Material 2).^35^ Descriptive statistics were used to analyse baseline survey data via IBM SPSS Statistics 27.

Baseline survey results were summarised in a nonidentifiable and aggregate format and presented to youth partners during the August and September 2021 LEWG monthly meetings. To facilitate the discussions, G. Y. L. and S. J. H. also summarised youth lived experience engagement frameworks and guidelines,^36^, ^37^, ^38^, ^39^ and youth partners were given time to discuss in smaller groups how current guidelines could be adjusted to enhance more meaningful partnerships with young people with lived experience across the YMH team's research activities. These meetings were recorded and subsequently transcribed by G. Y. L.

To facilitate in‐depth qualitative data analysis and support the continuous iteration and improvement of the LEWG protocol, Braun and Clarke's approach to thematic analysis was used. This approach facilitated both inductive and theory‐driven data analyses, meaning themes were derived from what was explicitly in the data as well as from the knowledge that researchers contributed to the data. Transcriptions were read multiple times by the first author (G. Y. L.). Themes and categories were then derived by G. Y. L. through descriptive coding and analytical memos,^40^ as well as from existing guidelines for participatory research.^22^, ^24^, ^25^, ^41^, ^42^, ^43^ Preliminary themes were shared with co‐authors (Y. J. C. S., A. H., S. J. H., and I. B. H.) through open discussions to support an iterative process whereby diverse knowledge contributed to the organisation of data into themes and categories. Those involved during the qualitative analysis process included academic researchers (G. Y. L., Y. J. C. S., I. B. H.) and lived experience researchers (A. H., S. J. H.).

Our qualitative data analysis followed constructivist grounded theory that assumes all knowledge is constructed by the meanings that individuals bring to data analysis.^44^ Our research team had existing sensitivities that influenced the organisation of data into themes. Academic researchers were more inclined to analyse data through the lens of theoretical frameworks for participatory research. In contrast, lived experience researchers were informed by their experience of mental ill‐health and of being part of an academic team, thus grounding proposed themes in the values, needs, and expectations of our youth partners.

## 3 RESULTS

### 3.1 Study #1: Youth partners’ perspectives on the acceptability of the LEWG protocol

Baseline surveys were administered to 11 youth partners in July 2021, including two who withdrew from the LEWG. An 82% response rate was achieved (*n* = 9), including two culturally and linguistically diverse respondents, two identifying with the LGBTQIA+ community, and one Aboriginal and/or Torres Strait Islander youth partner respondent. As presented in Table 1, survey results suggested that the key motivations and goals for youth partners in the LEWG were ‘to make sure others can access better care’ (*n* = 8), ‘to help others’ (*n* = 7), and ‘to understand latest research evidence’ (*n* = 6). Key benefits of working with the YMH team included ‘research team are sensitive to my needs’ (*n* = 6), ‘feel like contributions are important to research activity’ (n = 5), and ‘contributions have been subsequently put into action’ (*n* = 5). Despite this, only a minority of youth partners reported that they ‘can see changes made as a direct result of input’ (*n* = 3) from participating in the LEWG, or ‘feel empowered to share [my] mental health journey story’ (*n* = 2). This may help to explain why only two members reported that their experience working with LEWG had been ‘positive’. Areas for improvement included ‘more diverse young people from all backgrounds’ (*n* = 5), ‘better communication before research activities’ (n = 5), ‘better communication before research activities’ (*n* = 5), and ‘better communication of final research learnings’ (*n* = 4).

**Table 1:** Baseline survey responses from youth partners.

	Youth partners (*n* = 9) who agreed with this item	
	*n*	%
**Previous experience working in mental health research**		
Yes	5	56
No	4	44
**Key motivations for participating in research through the LEWG**		
To help others	7	78
To benefit personal mental wellbeing	5	56
Good reputation of YMH team at Brain and Mind Centre	3	33
To earn study payment	3	33
Encouraged by someone to participate	3	33
Positive experience participating in previous research study	2	22
Learn more about personal mental health	1	11
Have a friend participating in the LEWG	0	0
**Personal goals for participating in the LEWG**		
To make sure others can access better care (make a change)	8	89
To understand latest research evidence	6	67
Opportunity for my voice to be heard	5	56
Networking with others	5	56
Benefit personal wellbeing	4	44
**Overall experience working with LEWG**		
Positive	2	22
Neutral	1	11
Negative	1	11
No response	5	56
**Benefits working with the YMH team**		
Research team are sensitive to my needs	6	67
Feel like contributions are important to research activity	5	56
Contributions have been subsequently put into action	5	56
Feel confident supporting someone's mental health journey	3	33
Can see changes have been made as a direct result of input	3	33
Feel empowered to share about mental health journey	2	22
None of the above	1	11
**Areas of improvement**		
More diverse young people from all backgrounds	5	56
Better communication before research activities	5	56
Better communication of final research learnings	4	44
Better organisation	2	22
None of the above	2	22
*Other: Having opportunities where young people can lead*	2	22
**Will continue participating in current research study**		
Very likely	5	56
Somewhat likely	3	33
Somewhat unlikely	0	0
Very unlikely	0	0
No response	1	11
**Would recommend participating this study to others**		
Very likely	2	22
Somewhat likely	6	67
Somewhat unlikely	0	0
Very unlikely	0	0
No response	1	11

Abbreviations: LEWG, Youth Lived Experience Working Group; YMH, Youth Mental Health and Technology.

### 3.2 Directions for change of the LEWG protocol as identified by youth partners

Survey findings of the first study in conjunction with existing youth lived experience engagement frameworks and guidelines were utilised to guide discussions during the August and September 2021 LEWG meetings about youth partners’ experiences participating in the LEWG (Supplementary Material 3).^32^, ^37^, ^38^, ^39^ Youth partners suggested several improvements, increasing their autonomy to flexibly choose and change their level of involvement, in line with Arunkumar et al. and Kirwan et al.'s recommendations.^24^, ^25^, ^26^

#### 3.2.1 Implement clear processes for both youth partners and academic researchers

Whilst LEWG processes (such as the format and frequency of meetings, study design, and recruitment of participants) were initially co‐designed with lived experience researchers, more practical details, including how information was communicated were left to the discretion of academic researchers. This led to inconsistencies in how academic researchers included youth partners in their research. For instance, one academic researcher focused on presenting their research to youth partners with little opportunity for collaboration and open discussion to deliver feedback. LEWG members reported being “scared” that this academic would believe they had engaged in “ethical consultation” and believed they could “continue on with their work” when youth partners themselves believed that the academic researcher had “lectured” them instead. As a lived experience researcher expressed, “The purpose of this working group is not for [academic researchers] to give you a lecture. That was never the memo”. Accordingly, youth partners identified the need to have more capacity to choose the meetings they attended and to ensure that the information presented was more supportive and respectful of young people.

Subsequently, the LEWG planning group co‐developed a 12‐month schedule of working group meetings which included a list of research topics to ensure that youth partners’ priorities and interests were reflected. The LEWG Terms of Reference were also updated based on youth partner feedback (Supplementary Material 4), to provide further clarification regarding the purpose of the working group, and meeting structures, as well as to clearly articulate the LEWG's aims for a bi‐directional relationship whereby both youth partners and academic researchers can equally benefit. The updated Terms of Reference also emphasise the opportunities for youth partners to have the autonomy to produce their own research dissemination outputs, “…like podcasts and just doing more awareness, advocacy, and all that sort of stuff”, which was expressed by young people as a priority for more effective partnerships.

**Figure 2:**
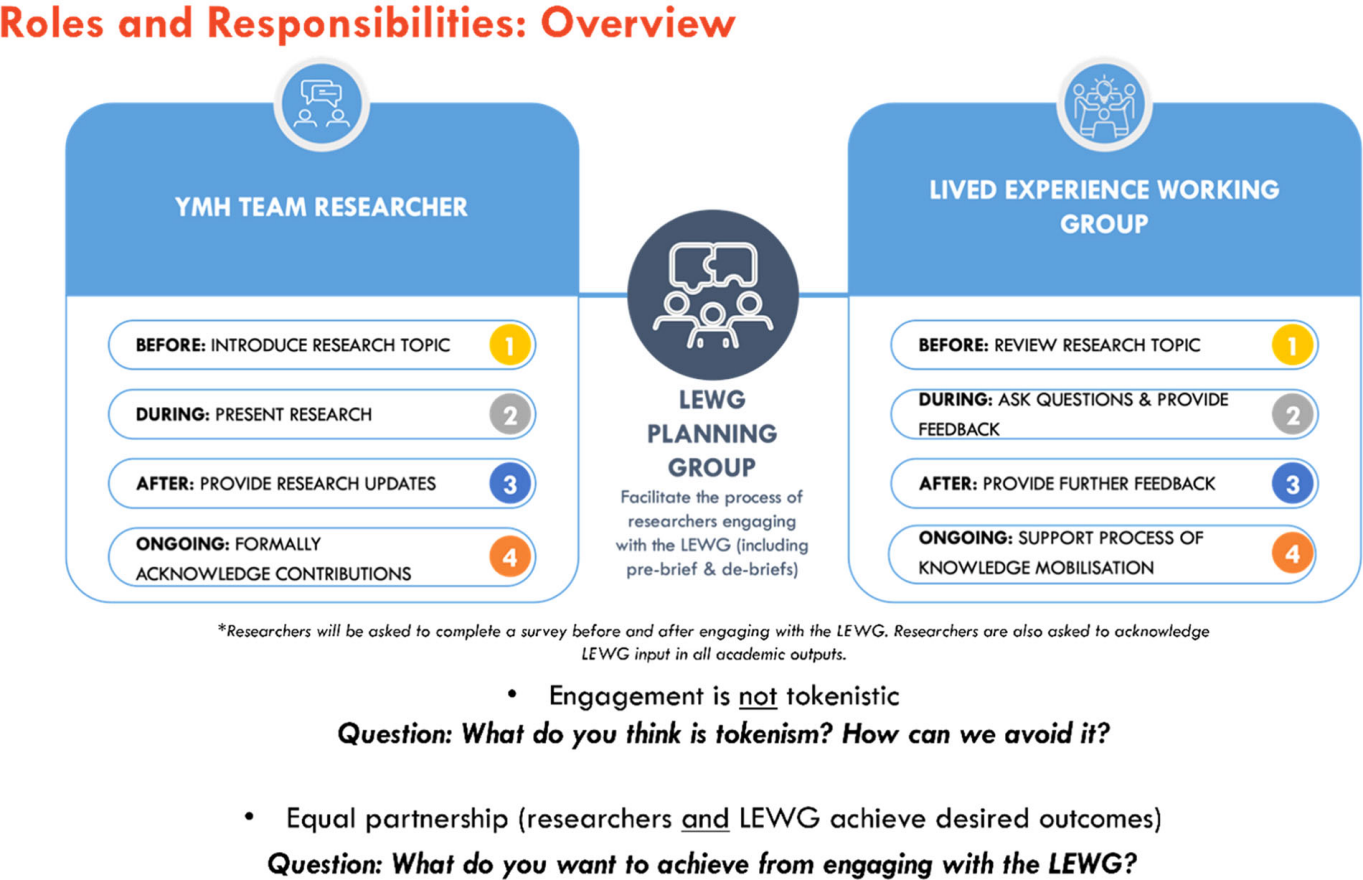
Example of a PowerPoint slide utilised in the pre‐briefing meetings with the Youth Lived Experience Working Group (LEWG) planning group and academic researchers. The pre‐briefing meeting aims to ensure that academic researchers receive important information and training on how to effectively partner with youth partners in their research.

Additionally, more formal pre‐briefing and de‐briefing sessions for both youth partners and academic researchers were identified by youth partners as an effective mitigation strategy. The aim of the *pre‐briefing* process (i.e., before academic researchers engage with youth partners via LEWG meetings) is to provide the opportunity for the LEWG planning group to meet with the academic researcher(s) to ensure researchers received appropriate information and training on how to effectively partner with young people with lived experience in their research. The meetings focused on open discussions and questions asked by the planning group to academic researchers (e.g., what does tokenism look like in research, and how will researchers avoid taking such an approach?). Figure 2 shows an example of a PowerPoint slide utilised in the prebriefing meetings with the LEWG planning group and academic researchers.

The pre‐briefing process also ensures academic researchers prepare an agenda and a one‐page summary of their research topic to circulate to youth partners at least one week before the LEWG meeting. To respond to feedback received by youth partners, both the agenda and summary documents are reviewed by the LEWG planning group before circulation to ensure that language is accessible. As expressed by one youth partner, “the language used by researchers needs to be heavily considered … [to be] less clinical, more conversational, empathetic, and informed”.

Reviewing the agenda and one‐page summary also ensures that youth partners receive an appropriate ‘content warning’ statement. This was identified as important by youth partners as demonstrated by the following comment, “…what I would like in a pre‐brief is a heads up on controversial triggering topics that might come up”. As part of the pre‐briefing process, the first 30 min of each LEWG meeting is spent with youth partners and the LEWG planning group—where trust had already been built—to review the one‐page researcher summary to jointly discuss potential feedback points and questions to the researcher(s).

The aim of the *de‐briefing* process (i.e., after academic researchers engaged with youth partners through the LEWG) is to ensure that there is an appropriate feedback mechanism implemented. Youth partners suggested spending the last 30 min of each LEWG meeting without the academic researcher(s) to ensure a respectful environment to give feedback (e.g., what worked well, areas of improvement, etc). A youth partner shared their reflections on what it meant for them to engage in a de‐brief without the academic researcher(s) as “…we were just able to be honest, and I really appreciated that. So if we could somehow find a compromise … of hearing from them [academic researcher] directly and having them able to answer our questions directly but then also having the space without having to walk on eggshells”.

Pre‐brief and debrief processes are further detailed in our Standing Operating Procedure, SOP (Supplementary Material 1) which includes information on how youth partner feedback is anonymously provided to academic researchers following the LEWG meeting by the planning group. This ensures that academic researchers are equally included in a process of reflection to learn and improve their research outputs, “…challenging researchers, not by making them feel like they're doing everything wrong, but actually making them motivated to see the opportunities in working with the LEWG”.

#### 3.2.2 Provide training opportunities for youth partners to develop research skills

A principle of good participatory research identified by Kirwan et al. is providing sufficient training.^26^ Similar sentiments were expressed by youth partners, who noted differing levels of research knowledge and capabilities amongst group members. One youth partner expressed that “it'd be good to have some sort of crash course [on the different phases of research], and then that way, whatever input that we do have we can also tailor it to become more applicable for the [academic] researchers”. Youth partners thus engaged in a short training session on the different phases of research delivered by the LEWG planning group in October 2021 (Supplementary Material 5). This training included opportunities for youth partners to ask questions and learn key academic terms, as well as to better understand the process of designing, conducting, and disseminating research (e.g., timelines involved in applying for research funding, receiving research ethics approvals, etc). Following training, pre‐briefing sessions with youth partners also included information on the current phase of the nominated research topic, in response to youth partners’ suggestions to include “… a timeline that's indicative of what is happening in each stage and where we are every time we have a meeting [with an academic researcher]”.

#### 3.2.3 Provide regular updates on how youth partner contributions led to research outcomes

Baseline survey results suggested that better communication of research learnings to youth partners was required, with only three youth partners able to ‘see changes that have been made as a direct result of input’. As noted by a youth partner, regular updates allowed LEWG members to see how their contributions led to research outcomes and improve accountability, “I'm also not entirely comfortable with spit‐balling ideas, and then like them taking off, and me not knowing what's going on… So it's not just about have you done it but it's about whether you've done it well”. Follow‐up points at 6‐ and 12‐months post academic researchers’ initial interaction with youth partners were included to formalise the provision of updates to youth partners in the revised LEWG protocol, as outlined in the SOP (Supplementary Material 1).

This pilot study has prompted necessary changes to the YMH team's LEWG protocol, to support participatory processes whereby young people with lived experience are empowered to make meaningful contributions and support improvements in mental health research. Box 1 details, from the perspective of a youth partner, their reflections on the LEWG.

Box 1. **Reflections from a youth partner on the LEWG**

**Table:** 

*‘*In my experience, the hardest thing about beginning a career in lived experience advocacy at a young age is having the courage to advocate for what is best for us. As young people, decisions about what is best for us are rarely our own—from what social events we attend to what meals we eat—with parents or carers having unique opinions of ‘best’ regarding screen time or play time, balanced against study and extra‐curricular commitments. To suddenly shift roles and advocate for ourselves in the formal processes of research can be remarkably daunting, especially for those who may not have been educated on all the options available for mental health care.
As discussed in this paper, young people can find themselves particularly marginalised in academic research settings. Although consumers are increasingly granted participation rights in individual treatment, and as lived experience advisors in research and policy development, power imbalances which favour psychiatric ‘expertise’ and marginalise the knowledge of consumers still exist. I hope co‐design practices will evolve in the future, allowing lived experience terminology and theory to be equally regarded as health professional and academic knowledge. This should hopefully be matched with adequate resources and infrastructure for designated peer‐led organisations and spaces, reflecting authentic consumer values at their core.
Though the bureaucratic system of academia will always present challenges, engaging in a co‐design process where our feedback was validated and implemented seamlessly drastically improved the processes of our working group. Having witnessed this process in action gives me renewed hope in bridging the gap between the highly regarded practices of academic research and the unique knowledge of lived experience. Though many gaps in mental health care still exist in Australia, being involved in this research initiative is a crucial step forward to recognise barriers and more importantly, to collectively – with academic researchers and youth partners equally – find ways forward to standardise genuine inclusion of youth lived experience voices in mental health research.'

### 3.3 Study #2: Exploring academic researchers' perspectives to improve the feasibility of the LEWG protocol

Baseline surveys were also administered to academic researchers between February and March 2022 about their experiences including young people with lived experience of mental ill‐health in their research. After removing duplicate responses submitted under the same IP address, 89 unique surveys were submitted by academic researchers, of which only 42 were included for analysis. 47 surveys were excluded for analysis as surveys were significantly incomplete, with eight respondents only completing survey questions regarding their work (i.e., area of research focus, etc.), 16 giving their online consent but not completing any further questions, two not giving their consent, and 21 respondents submitting a blank survey. Most academic researchers whose responses were included for analysis were based in Australia (90%, *n* = 38), with some respondents from Canada, the United States of America, and the United Kingdom (10%, *n* = 4). As presented in Table 2, over half of the academic researchers had previous experience working with young people with lived experience in their research (60%, *n* = 25). Of those who have worked with lived experience participants, 56% (*n* = 14) had funding available to include youth partners in their research. Academic researchers reported varying levels of including young people with lived experience in their research, with most having worked with young people with lived experience through a steering or advisory group (56%, *n* = 14). In contrast, no researchers reported working with young people with lived experience to seek research funding.

**Table 2:** Baseline survey responses from academic researchers from the Youth Mental Health and Technology team, including national and international research collaborators.

	Academic researchers who agreed with this item (*N* = 42, unless otherwise specified)	
	*n*	%
**Previous experience working with youth partners in research (** ***N*** **= 42)**		
Yes	25	60
No	17	40
**Funding availability for inclusion of youth partners in research (** ***N*** **= 25)**		
Yes	14	56
No	9	36
Unsure	1	4
*Other: Funding available but not for young people*	1	4
**Research stage(s) youth partners are/were included in research (** ***N*** **=** **25)**		
Managing the research project (e.g., steering/advisory group)	14	56
Designing the research project	13	52
Conducting the research	12	48
Dissemination (e.g., co‐publishing)	8	32
Identifying the research topic	7	28
Evaluation	7	28
Analysing and interpreting findings	6	24
Acting on the findings	4	16
Seeking funding	0	0
**Extent youth partners are/were included in research (*N*** **=** **25)**		
Provide(d) consultation on specific project components	18	72
Are/were partners on projects designed and developed by researchers	11	44
Are/were included, but their contributions are/were minimal	8	32
Lead/led projects and initiate(d) project action	6	24
Informed but do/did not contribute to its design/operationalisation	5	20
Equally share(d) decision‐making responsibilities	3	12
**How researchers can improve experiences of youth partners (*N* = 42)**		
Inclusion of youth partners from the design stage of research planning	30	71
Clear communication how contributions led to outcomes	30	71
Inclusion of young partners from diverse backgrounds	27	64
Better organisation of research activities	21	50
None of the above	2	5
No response	5	12

**Figure 3:**
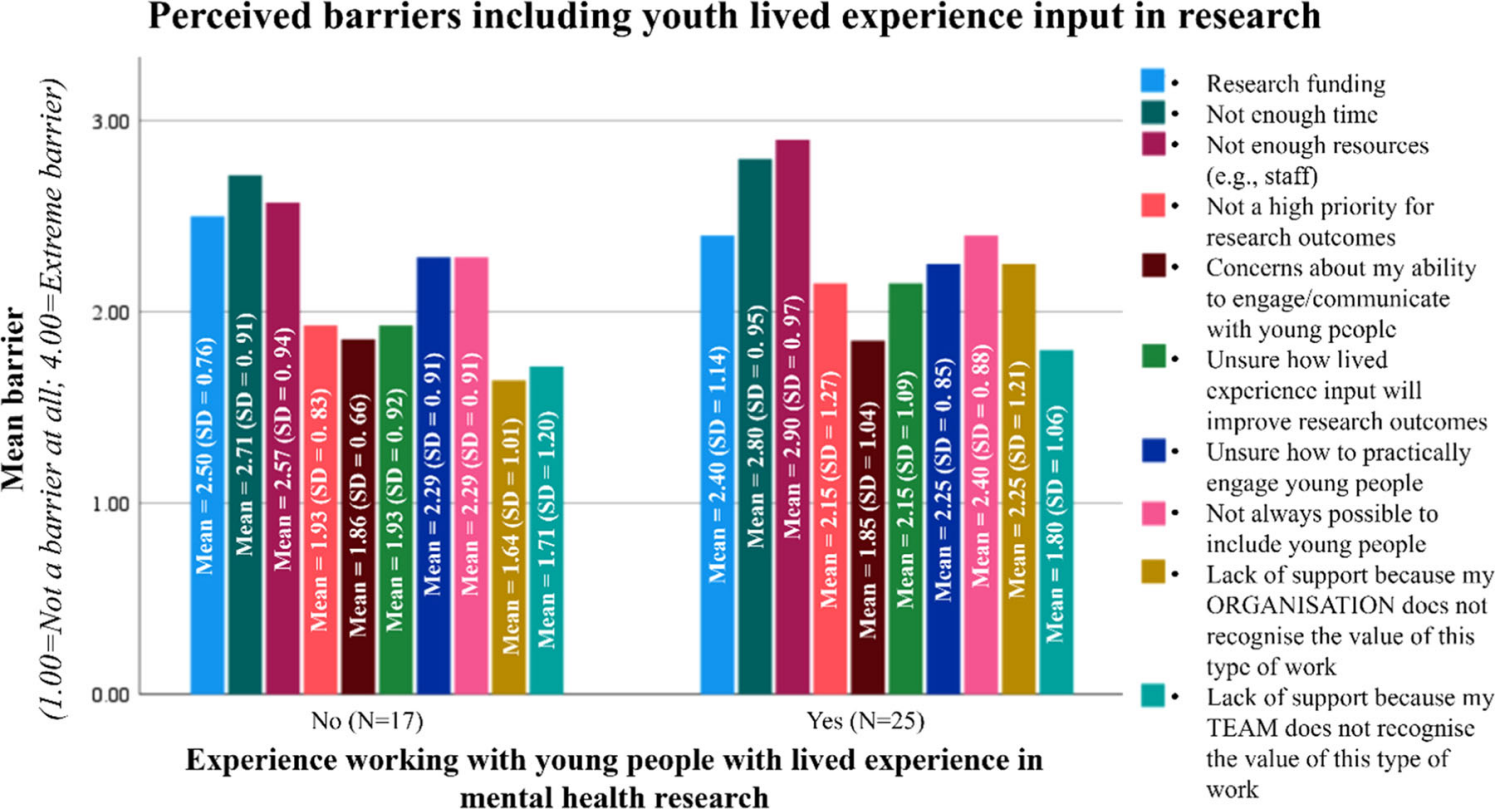
Perceived barriers identified from the perspective of academic researchers. Means and SDs reported as data were derived from Likert‐scale survey responses.

Most academic research respondents consulted young people on specific project components (72%, *n* = 18), and only 12% (*n* = 3) reported that young people had (or currently have) equal decision‐making. Interestingly, with the exception of ‘research funding’, ‘concerns about the ability to engage/communicate with young people with lived experience’, and ‘unsure how to practically engage young people with lived experience’, academic researchers who have worked with young people reported higher statistical means to barriers including young people with lived experience in their research, compared to academic researchers without experience working with young people (Figure 3).

Despite this, 71% (*n* = 30) of academic researchers agreed that young people should be included in the design stage of research planning, and 71% (*n* = 30) agreed that researchers should provide clear communication to young people about how their contributions led to research outcomes, and 64% (*n* = 27) agreed partnerships with young people from diverse backgrounds should be formed, suggesting most academic researchers value the meaningful inclusion of young people with lived experience in research even in the face of the barriers that may exist.

## 4 DISCUSSION

This paper describes a pilot evaluation of a LEWG that was established to provide input on all projects conducted by the YMH research team and describes changes that were made to the LEWG protocol as a result of this evaluation. In summary, youth partners identified the need for more transparency from both sides; researchers needed to be clearer about the influence of LEWG on their work, and LEWG members wanted to be able to provide feedback to researchers when issues arose and to have clearer guidelines and training on the role and functions of the group. Youth partners also wanted more autonomy, by producing original research dissemination outputs such as podcasts, webinars, and research papers, and more representation of diverse cultural groups. These changes were strongly supported by survey results from academic researchers, who agreed that processes such as clearer communication about how young people have contributed were valuable and achievable. Researchers also identified barriers that exist in academic settings preventing genuine partnerships with young people with lived experience. For example, most researchers cited lack of funding and time as key reasons why they could not include youth partners in their research, which may explain why no academic researchers reported working with young people with lived experience to seek research funding. As such, the updated protocol more explicitly states that the role of the planning group is to support and offload some of the administrative burdens of engaging with the LEWG from researchers, for instance by communicating with members, facilitating meetings, and providing resources.

This work contributes to the existing literature by describing a protocol for engaging a youth working group to advise on research projects, whilst also providing insightful feedback from youth partners and academic researchers on the acceptability and feasibility on this LEWG protocol. There is a dearth of long‐term evaluative work in this field, partly due to a lack of transparency from research teams regarding their protocols and because of limited opportunities for long‐term collaborations, particularly with vulnerable populations.^9^, ^11^, ^12^ Although the findings presented in this paper are preliminary, they provide important learnings regarding the complexity of creating research processes that are fully inclusive and supportive of young people. In line with existing frameworks that have advocated for a flexible and inclusive approach to participatory research,^22^, ^24^, ^25^ we deliberately sought to empower our lived experience researchers and youth partners to design LEWG processes and procedures. Yet, due to this flexible approach, we missed opportunities to learn from previous protocols. For instance, Hawke et al.'s practical guidelines were heavily relied on to implement improvements to our LEWG protocol, such as the introduction of prebriefs and debrief, and more formal processes for academic researchers to regularly update youth partners on how their contributions led to research outcomes.^36^ As such, whilst there is unlikely to be a ‘one size fits all approach’ to participatory research design, gold‐standard frameworks are needed to ensure that research procedures are as inclusive and respectful as possible. Overall, the YMH LEWG provides a rare opportunity to assess how young people can be empowered to continue shaping their involvement in research projects over time, in partnership with academic researchers, so that both groups derive the greatest value from this work and so that the contributions of lived experience expertise are not tokenistic.^45^

Despite these valuable contributions, it is important to acknowledge several limitations of our work including the number of young people and academic researchers who participated in online surveys. Given that our working group has nine members, we had limited opportunities to gain feedback on our LEWG protocol from young people with lived experience of mental ill‐health more broadly. This demonstrates the importance of adopting qualitative methodologies in future evaluations, that can provide more in‐depth and nuanced knowledge about LEWG processes.

A further limitation was that 40% of academic researchers who completed the surveys had no previous experience working with young people with lived experience in their research. Their response was included in the analysis to ensure that the objective of the pilot study, which was to improve the acceptability and feasibility of the LEWG protocol based on the perspectives of youth partners and academic researchers, could be achieved. The exploration of academic researchers’ perspectives—including those with and without experience partnering with young people with lived experience in their research—thus enabled a more holistic understanding of what may facilitate and limit partnerships with young people with lived experience in mental health research.

Our pilot study uncovered important learnings to support more robust partnerships with young people with lived experience and academic researchers in the context of the LEWG. Ongoing long‐term work will be vital to ensure that the narrative in mental health research continues to shift towards the direction that authentically values and supports non‐tokenistic partnerships with young people with lived and living experiences in mental health research. Future research will explore and report the longitudinal impacts of the LEWG on the YMH team's research projects and outputs.

## 5 CONCLUSION

Embedding the voices of young people with lived and/or living experiences of mental ill‐health in all stages of health research can help to address the translation gap between research and practice, can empower young people, and can foster the next generation of lived experience researchers. Yet, there is currently a lack of transparency regarding participatory research procedures, limiting the evaluation of these processes and knowledge about gold‐standard participatory approaches. The current paper outlines the baseline results of a pilot evaluation of our working group and details key changes that have been adopted to enhance the LEWG's participatory processes. This paper also contributes more broadly to a growing international area of research and sheds some initial insights into the facilitators, motivators, and barriers to youth lived experience inclusion in mental health research from the perspectives of youth partners and academic researchers, working towards a more equitable researcher‐youth lived experience partnership model.

## AUTHOR CONTRIBUTIONS

Though not all authors have lived experience of mental ill‐health, we recognise the strengths and knowledge that each author has contributed. All authors listed in this paper are committed to empowering youth lived experience voices, and the expertise of young people with lived and living experience has taken precedence throughout the paper (Alexis Hutcheon, Samuel J. Hockey, Rachael Laidler, Claudia Perry, Tara Lindsay‐Smith, Annabel Ramsay, Skye Choi, Dakota Feirer, Andrew W. Shim, Jessica Cottle, Anith Mukherjee, Joshua New, and Rebecca Yu). Grace Yeeun Lee designed the study, collected evaluation data, and analysed the data independently with supervision by Ian Bernard Hickie. She co‐wrote the paper with Sarah McKenna, who was not involved in designing the study, nor in the collection and analysis of data, but supported the write‐up of the manuscript draft—namely the Introduction and Discussion. Rachael Laidler wrote a reflective opinion piece that is included as Box 1 in the Results. Grace Yeeun Lee, Yun Ju C. Song, Alexis Hutcheon, and Samuel J. Hockey managed the coordination of the LEWG during 2021 and 2022. Grace Yeeun Lee co‐developed the baseline surveys with Alexis Hutcheon, Samuel J. Hockey, and Louise Freebairn. Sarah McKenna and Samuel J. Hockey will manage the future coordination of the LEWG, including the ongoing evaluation study, from 2023. Yun Ju C. Song, Elizabeth Mary Scott, and Ian Bernard Hickie facilitated the acquisition of financial support leading to this publication. All authors contributed intellectually to the drafts and approved the final manuscript.

## CONFLICTS OF INTEREST STATEMENT

J. O. is both Head of Systems Modelling, Simulation & Data Science at The University of Sydney's Brain and Mind Centre and Managing Director of Computer Simulation & Advanced Research Technologies (CSART). E. M. S. is the Discipline Leader of Adult Mental Health, the School of Medicine, University of Notre Dame, an Associate Professor at The University of Sydney and a Consultant Psychiatrist at Mind Plasticity. She has received honoraria for educational seminars related to the clinical management of depressive disorders supported by Servier and Eli‐Lilly Pharmaceuticals. She has participated in a national advisory board for the antidepressant compound Pristiq, manufactured by Pfizer. She was the National Coordinator of an antidepressant trial sponsored by Servier. L. F. is an affiliate at the Brain and Mind Centre, University of Sydney, and is employed at ACT Health as Director of Knowledge Translation and Health Outcomes, Epidemiology Section, ACT Health. I. B. H. is supported by an NHMRC L3 Investigator Grant (GNT2016346) and is the Co‐Director, and Health and Policy at The University of Sydney's Brain and Mind Centre (BMC). The BMC operates an early‐intervention youth service at Camperdown under contract to Headspace. He is the Chief Scientific Advisor to, and a 3.2% equity shareholder in, InnoWell Pty Ltd. InnoWell was formed by The University of Sydney (45% equity) and PwC (Australia; 45% equity) to deliver the $30M Australian Government‐funded Project Synergy (2017–2020; a 3‐year Program for the transformation of mental health services) and to lead transformation of mental health services internationally through the use of innovative technologies. The remaining authors declare no conflict of interest.

## Supporting information

Supporting Information.

Supporting Information.

Supporting Information.

Supporting information.

Supporting Information.

Supporting Information.

Supporting Information.

## Data Availability

The data sets used and/or analysed during the current study are available from the corresponding author upon reasonable request.
