# Supplementary material for: Strengthening mental health research outcomes through genuine partnerships with young people with lived or living experience: A pilot evaluation study
Source: Health Expect. 2023 May 17;26(4):1703–15. doi: 10.1111/hex.13777 (PMC10349217; doi:10.1111/hex.13777)
Supplement: Supplementary file 1 — Supporting Information. [file HEX-26--s007.pptx]

## Slide 1
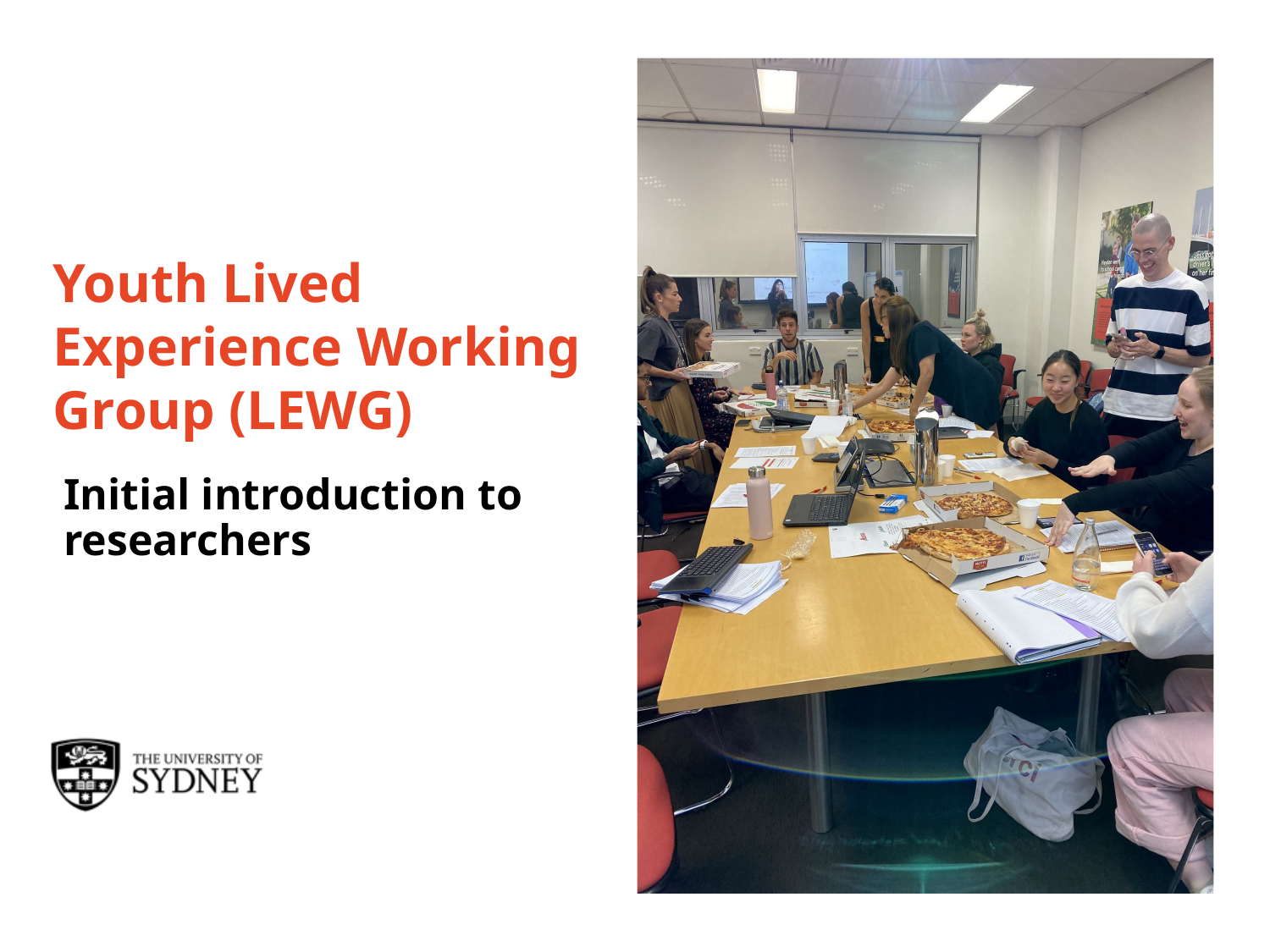

# Youth Lived Experience Working Group (LEWG)
Initial introduction to researchers

## Slide 2
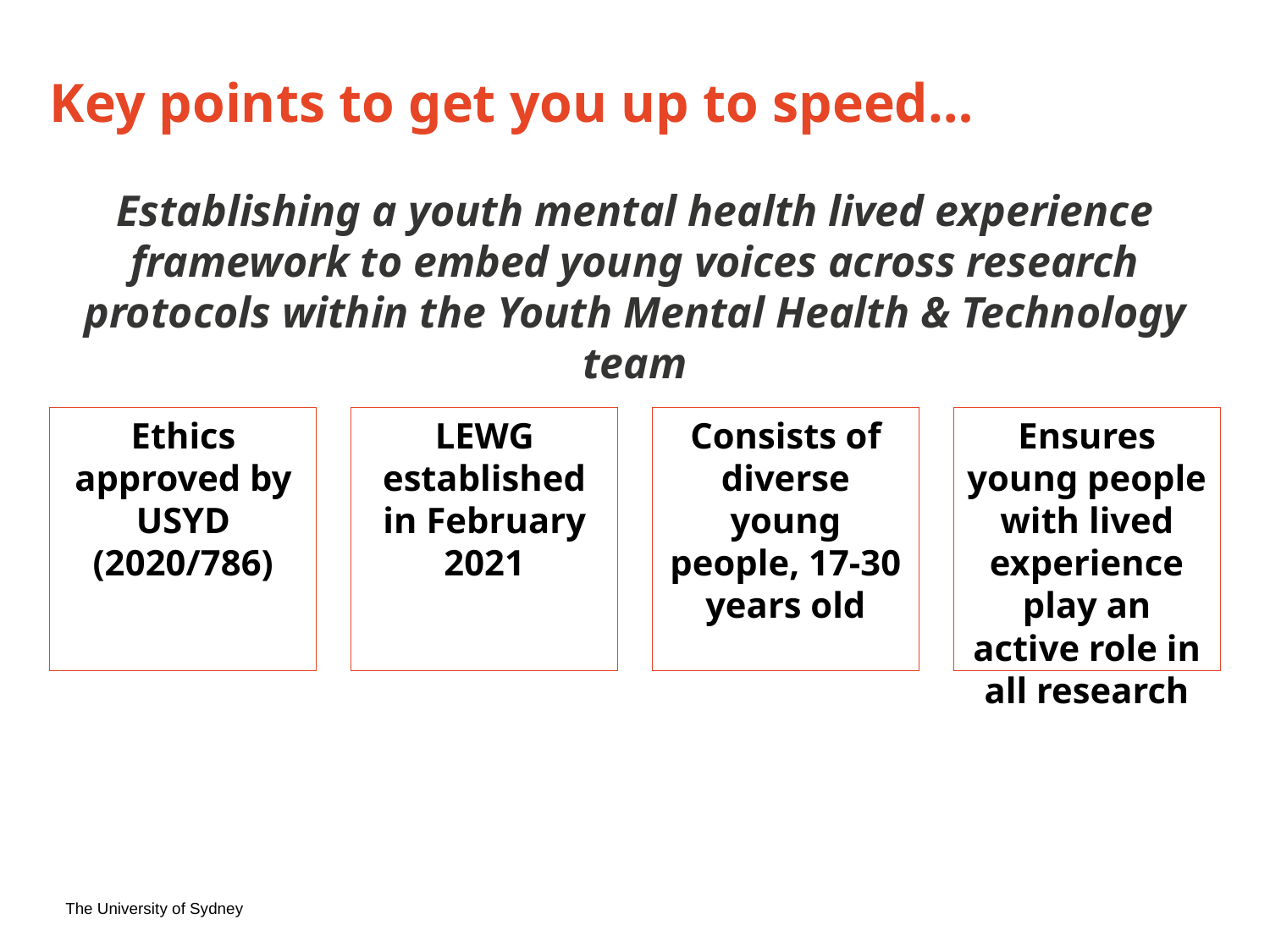

# Key points to get you up to speed…
Establishing a youth mental health lived experience framework to embed young voices across research protocols within the Youth Mental Health & Technology team
Ethics approved by USYD (2020/786)
LEWG established in February 2021
Consists of diverse young people, 17-30 years old
Ensures young people with lived experience play an active role in all research

## Slide 3
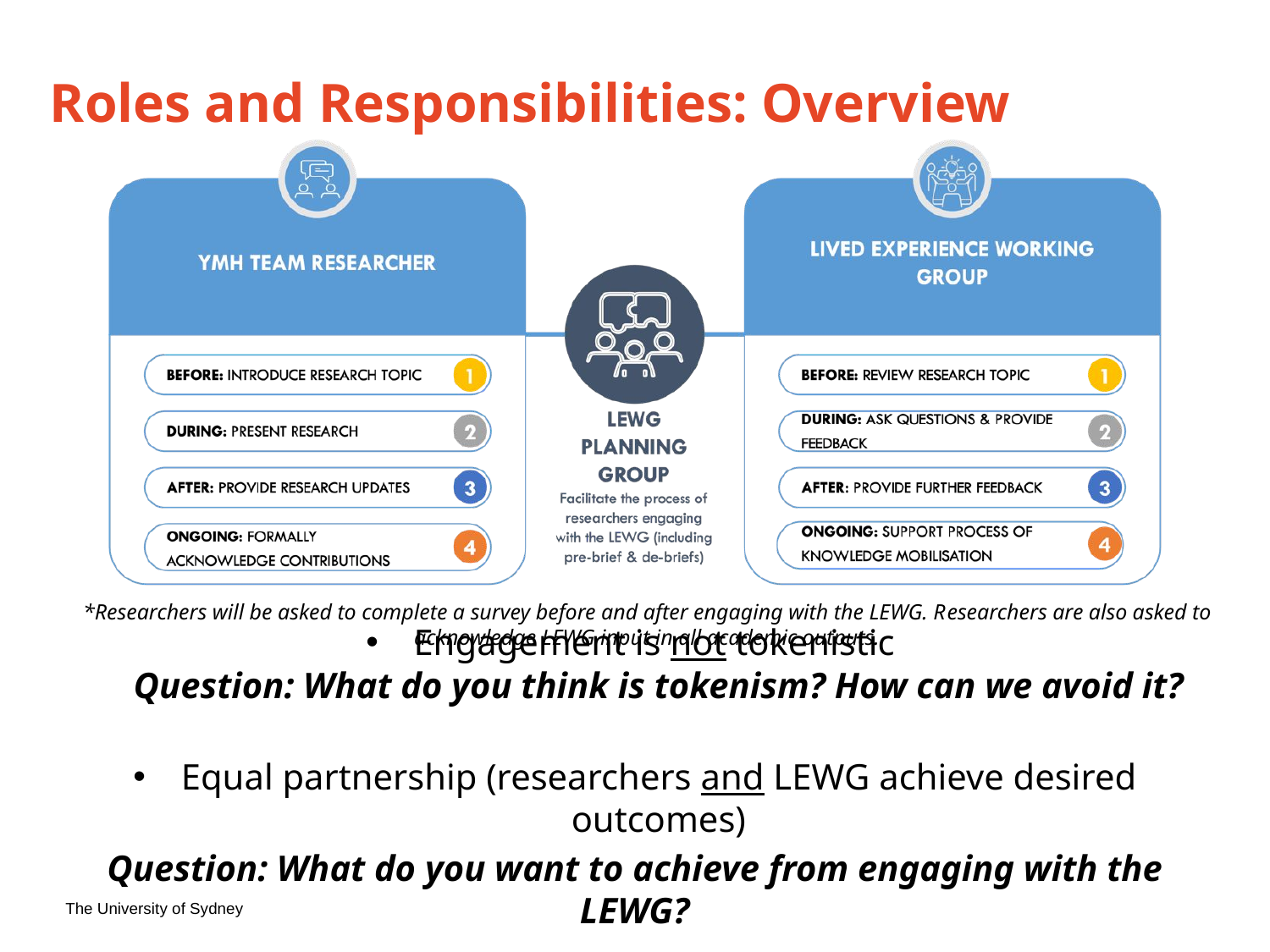

# Roles and Responsibilities: Overview
*Researchers will be asked to complete a survey before and after engaging with the LEWG. Researchers are also asked to acknowledge LEWG input in all academic outputs.
Engagement is not tokenistic
Question: What do you think is tokenism? How can we avoid it?
Equal partnership (researchers and LEWG achieve desired outcomes)
Question: What do you want to achieve from engaging with the LEWG?

## Slide 4
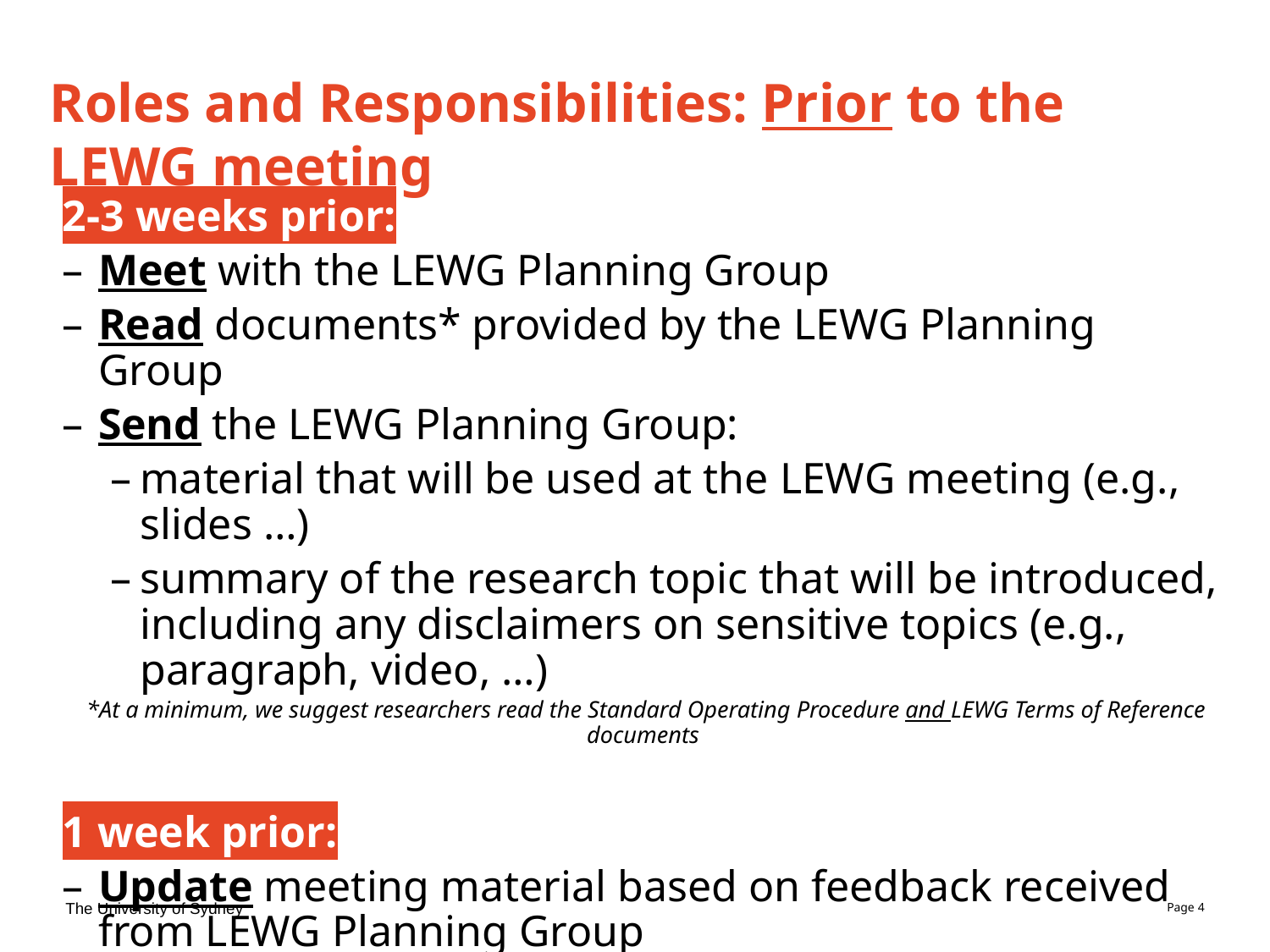

# Roles and Responsibilities: Prior to the LEWG meeting
2-3 weeks prior:
Meet with the LEWG Planning Group
Read documents* provided by the LEWG Planning Group
Send the LEWG Planning Group:
material that will be used at the LEWG meeting (e.g., slides …)
summary of the research topic that will be introduced, including any disclaimers on sensitive topics (e.g., paragraph, video, …)
*At a minimum, we suggest researchers read the Standard Operating Procedure and LEWG Terms of Reference documents
1 week prior:
Update meeting material based on feedback received from LEWG Planning Group
Complete a survey

## Slide 5
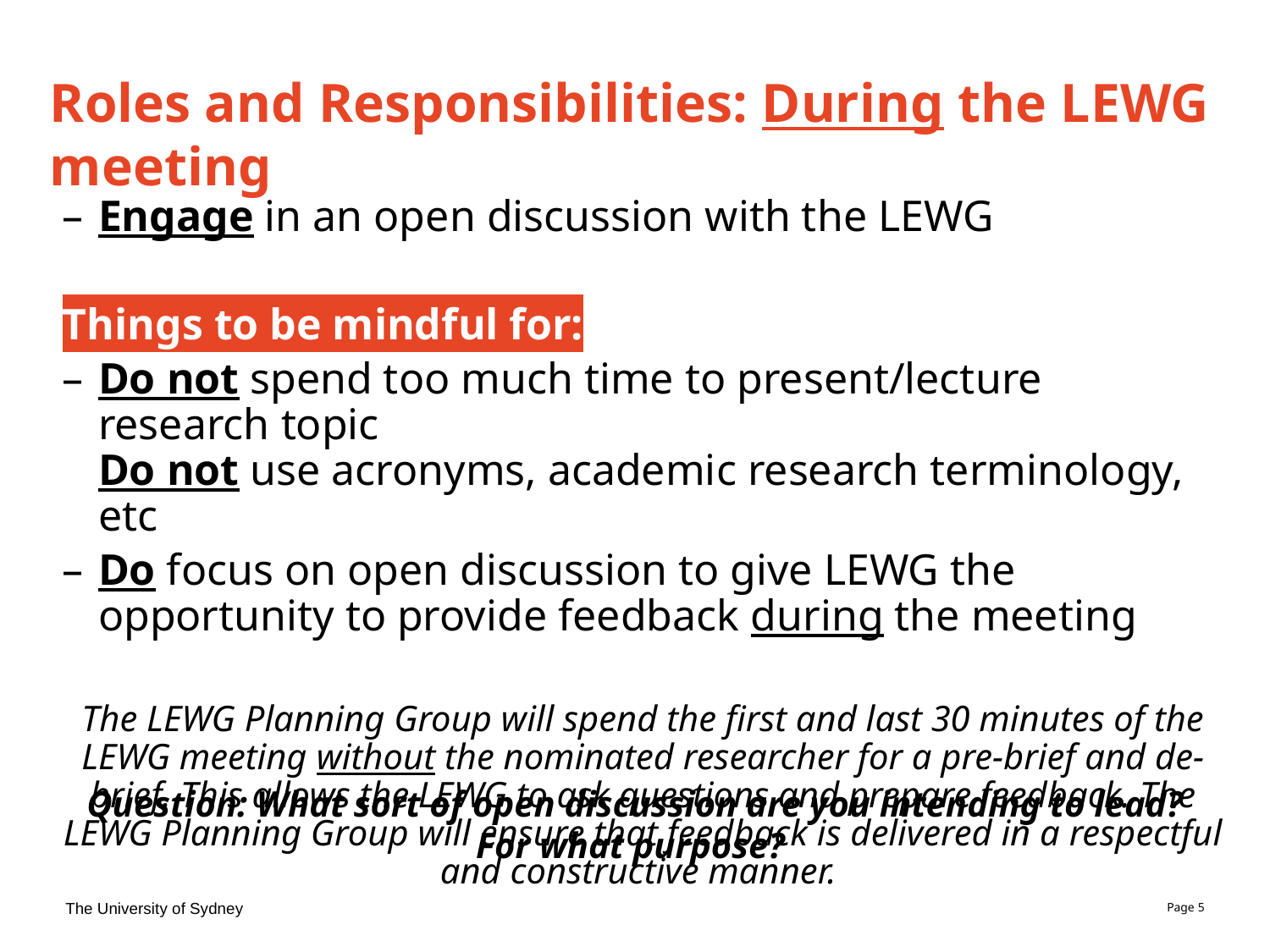

# Roles and Responsibilities: During the LEWG meeting
Engage in an open discussion with the LEWG
Things to be mindful for:
Do not spend too much time to present/lecture research topic
Do not use acronyms, academic research terminology, etc
Do focus on open discussion to give LEWG the opportunity to provide feedback during the meeting
The LEWG Planning Group will spend the first and last 30 minutes of the LEWG meeting without the nominated researcher for a pre-brief and de-brief. This allows the LEWG to ask questions and prepare feedback. The LEWG Planning Group will ensure that feedback is delivered in a respectful and constructive manner.
Question: What sort of open discussion are you intending to lead? For what purpose?

## Slide 6
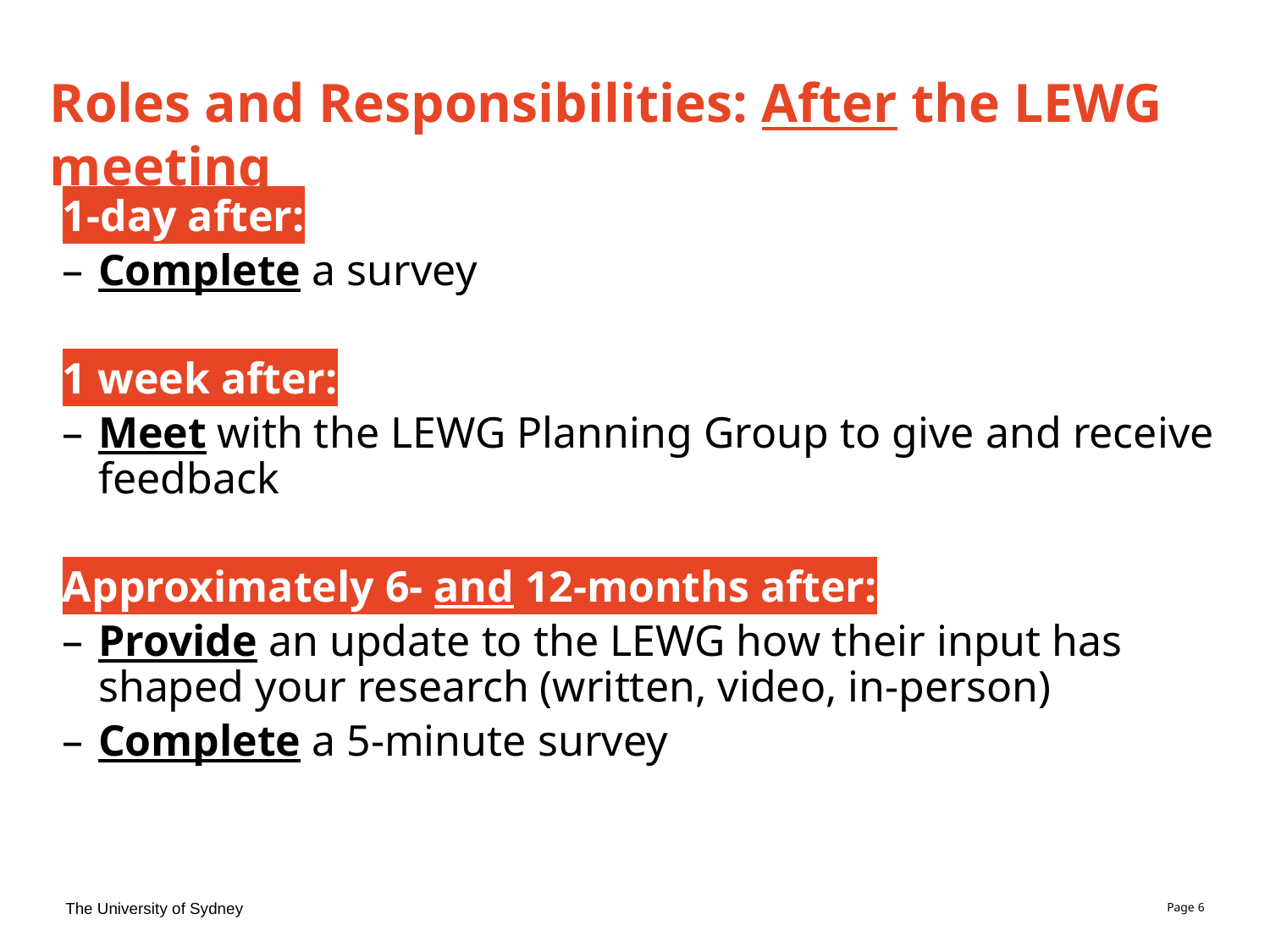

# Roles and Responsibilities: After the LEWG meeting
1-day after:
Complete a survey
1 week after:
Meet with the LEWG Planning Group to give and receive feedback
Approximately 6- and 12-months after:
Provide an update to the LEWG how their input has shaped your research (written, video, in-person)
Complete a 5-minute survey

## Slide 7
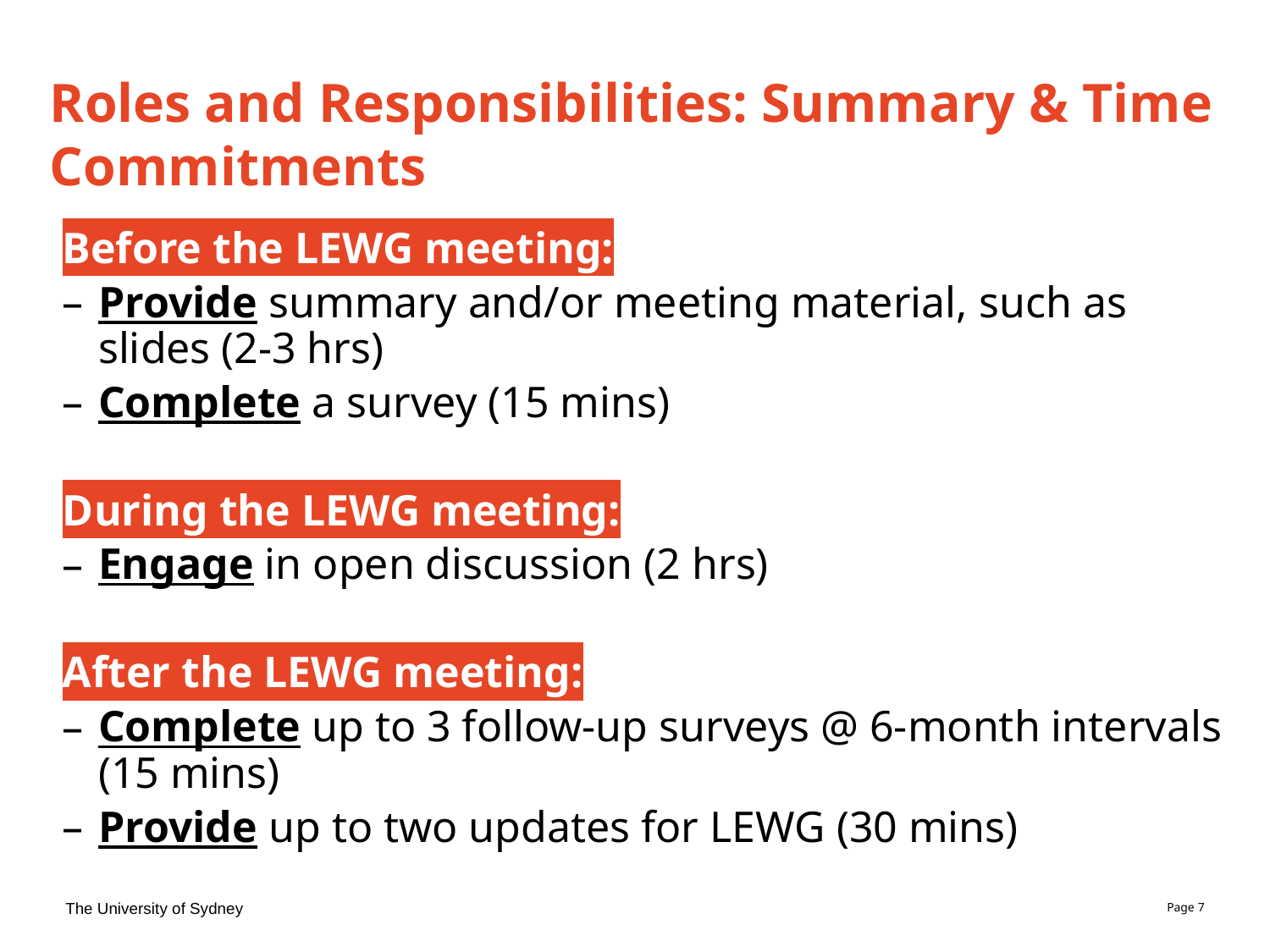

# Roles and Responsibilities: Summary & Time Commitments
Before the LEWG meeting:
Provide summary and/or meeting material, such as slides (2-3 hrs)
Complete a survey (15 mins)
During the LEWG meeting:
Engage in open discussion (2 hrs)
After the LEWG meeting:
Complete up to 3 follow-up surveys @ 6-month intervals (15 mins)
Provide up to two updates for LEWG (30 mins)

## Slide 8
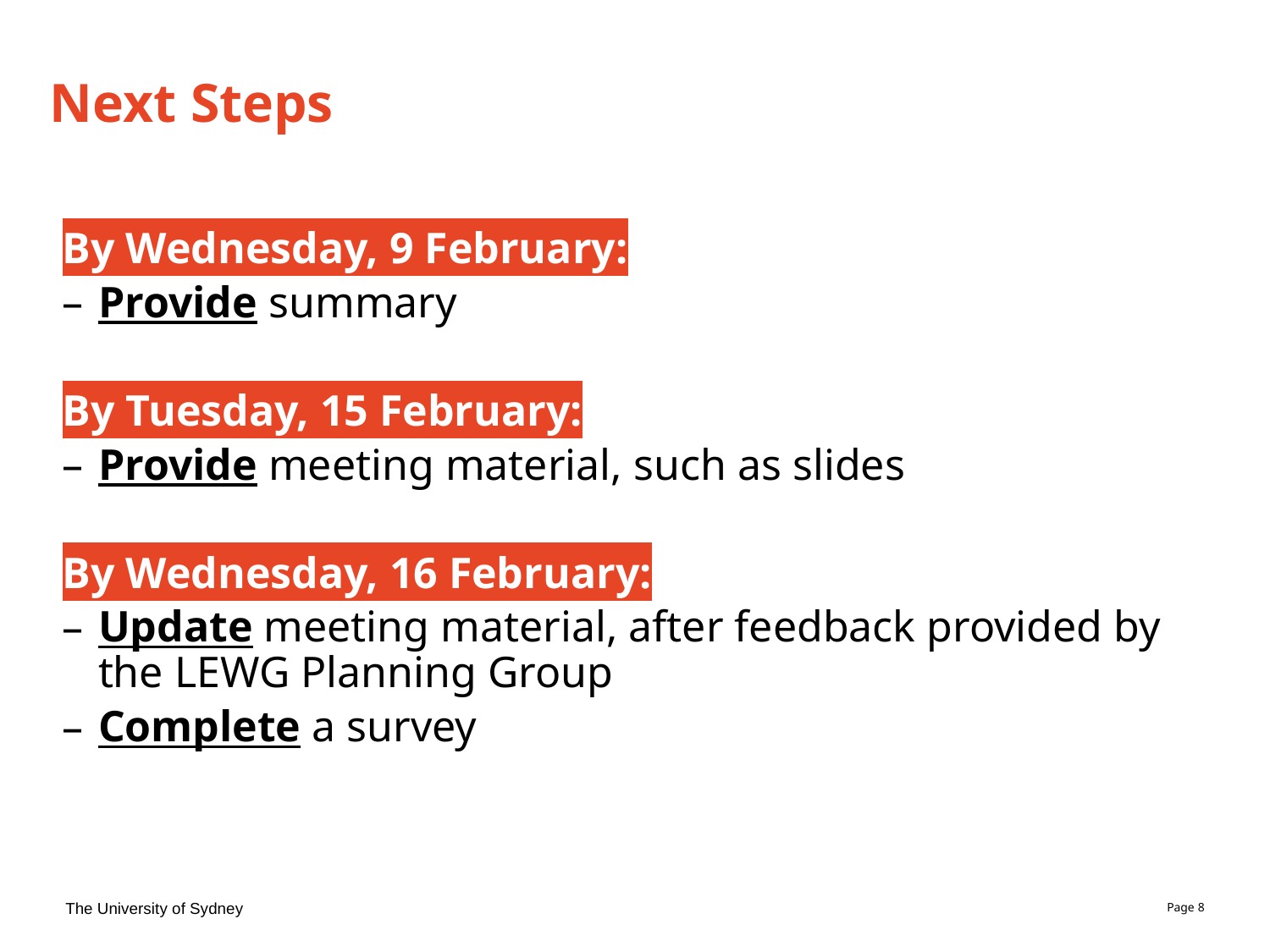

# Next Steps
By Wednesday, 9 February:
Provide summary
By Tuesday, 15 February:
Provide meeting material, such as slides
By Wednesday, 16 February:
Update meeting material, after feedback provided by the LEWG Planning Group
Complete a survey

## Slide 9
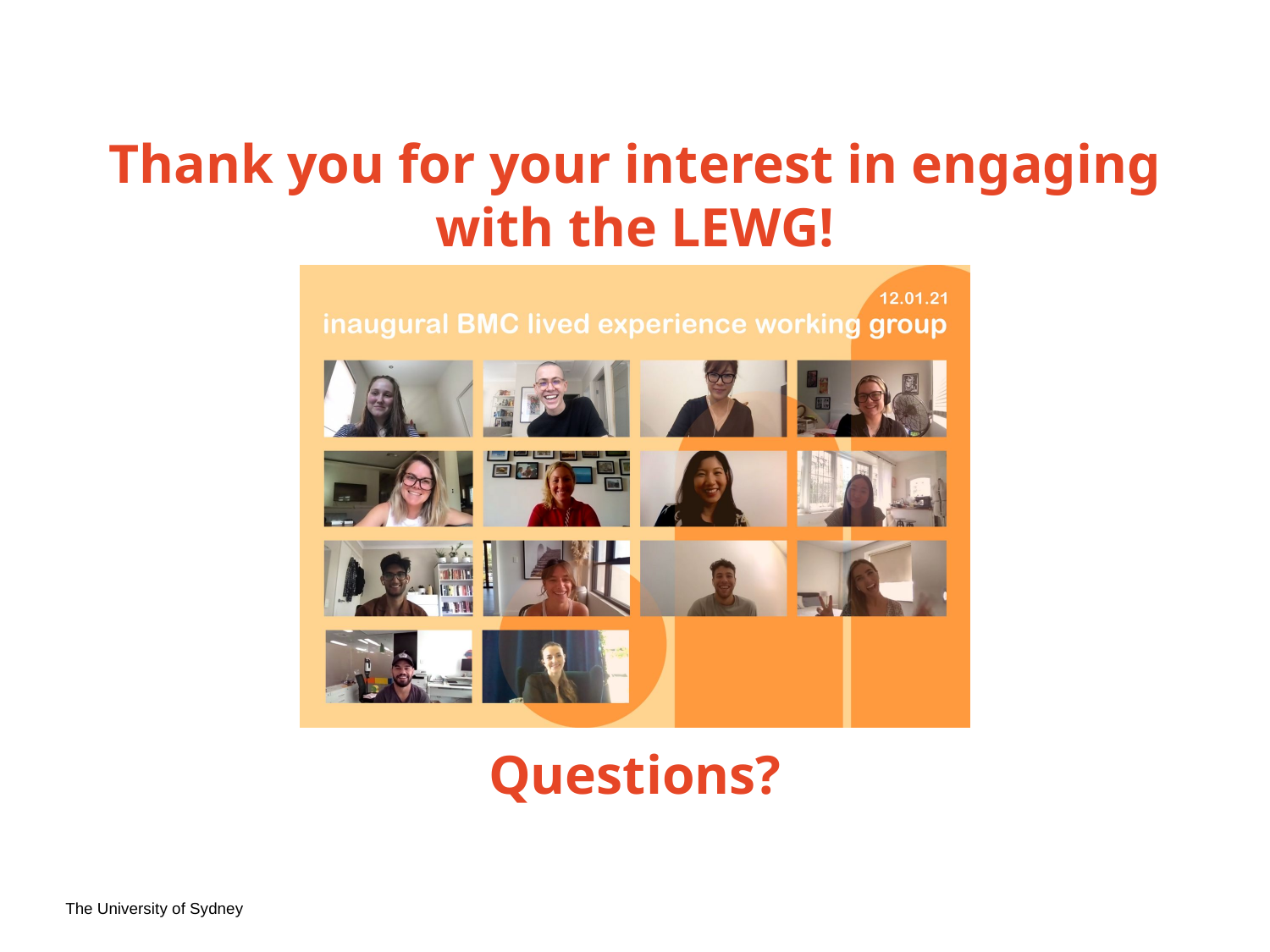

Thank you for your interest in engaging with the LEWG!
# Questions?
