# Supplementary material for: Strengthening mental health research outcomes through genuine partnerships with young people with lived or living experience: A pilot evaluation study
Source: Health Expect. 2023 May 17;26(4):1703–15. doi: 10.1111/hex.13777 (PMC10349217; doi:10.1111/hex.13777)
Supplement: Supplementary file 2 — Supporting Information. [file HEX-26--s001.docx]

**Standard Operation Procedure: Engaging and maintaining ongoing relationships with the Lived Experience Working Group (roles and responsibilities)**

***Establishing a youth mental health lived experience framework to embed young voices across research protocols within the Youth Mental Health & Technology team (Brain and Mind Centre)***

**Protocol Number:** 2020/786

**Sponsor:** The University of Sydney

1. **Introduction**

The Lived Experience Working Group (LEWG) was established in February 2021 to ensure all research led by the Brain and Mind Centre’s Youth Mental Health (YMH) and Technology team is conducted in consultation with young people with lived (or living) experience of mental illness. The LEWG members are viewed as experts in their experience, and thus are considered equal research partners.

The LEWG consists of ten culturally and linguistically diverse young people aged 17-30 years old. This Standard Operating Procedure (SOP) document aims to ensure that YMH researcher engagement with the LEWG is productive and importantly, respectfully done in a non-tokenistic way. As such, this document outlines the steps involved to inform YMH team researchers on how to positively engage and maintain ongoing relationships with the LEWG.

This SOP reflects feedback received by the LEWG.

1. **Roles and responsibilities**

The LEWG planning group consists of four members:

1. Samuel Hockey
2. Alexis Hutcheon
3. Grace Lee
4. Christine Song

The LEWG planning group plays an important role to facilitate the process of ensuring that nominated YMH researchers positively engage with the LEWG – prior to, on the day of, and ongoingly after the LEWG meeting. Figure 1 provides an overview of roles and responsibilities of the nominated YMH researcher, the LEWG planning group, and the LEWG. This is detailed in Sections 4 – 7.


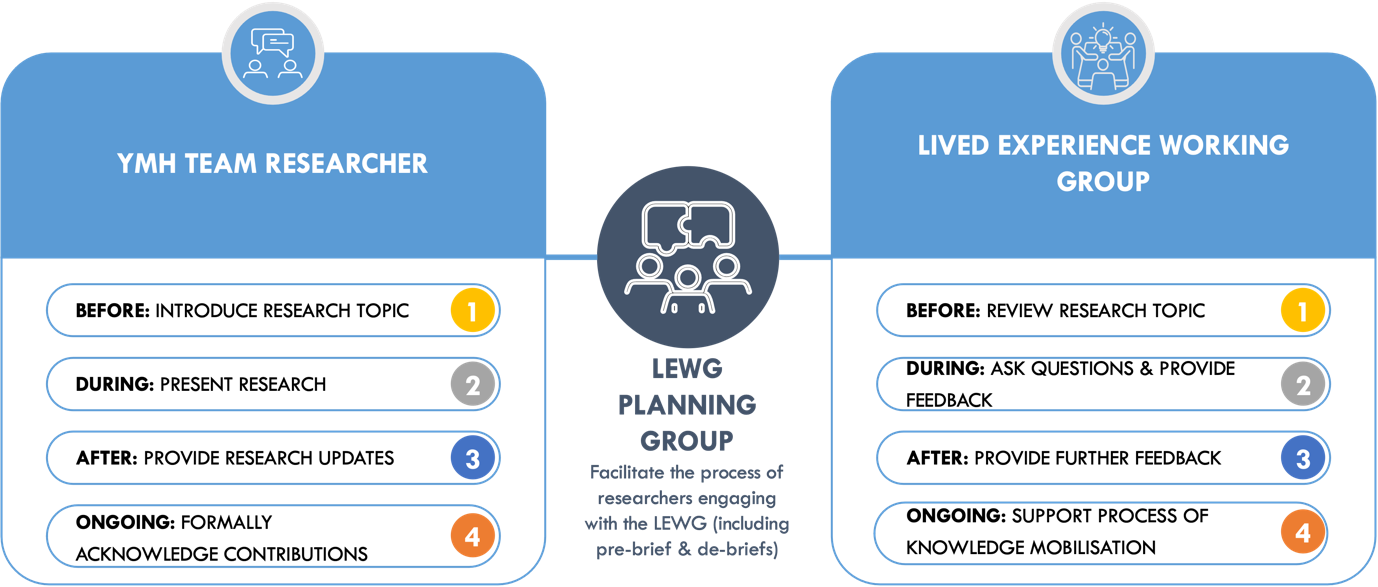


**Figure 1.** Overview of the roles and responsibilities of the nominated YMH team researcher, LEWG planning group and the LEWG.

1. **Recruiting YMH researchers**

The LEWG planning group will facilitate the recruitment of researchers to engage with the LEWG to ensure a balance of researchers participating on a needs’ basis (i.e., YMH researchers identify a need to engage lived experience participants in their research) as well as presenting the LEWG with topics that are of interest to the group.

The LEWG planning group will send an email (Bcc’ed to remove any personal participant information) via the email address at the beginning of each calendar year. The email will include information about the baseline researcher survey, as well as an invitation for researchers to contact the LEWG planning group if they are interested in engaging with the LEWG.

Once a list of YMH researchers are identified, the LEWG planning group will facilitate the process of scheduling – and importantly, informing the LEWG on any updates – to ensure that there is continuous communication and room for feedback to improve processes.

Once a YMH researcher is confirmed, the researcher, LEWG planning group and the LEWG will engage in the steps outlined in Sections 4 – 7 below.

1. **Prior to the Lived Experience Working Group meeting**

| **YMH Team Researcher** | **LEWG Planning Group** | **LEWG** |
| --- | --- | --- |
| 2-3 weeks prior | | |
| - Read documents provided by the LEWG planning group - Circulate meeting material (e.g., slides) to the LEWG planning group at least one week prior to the LEWG meeting - Provide a summary of the research topic and include any disclaimers on sensitive topics | - Meet with the YMH researcher and provide an overview of research aims - Schedule a meeting one week prior to the LEWG meeting to finalise material that will be presented (e.g., slides) - Circulate documents:   - *SOP – Engaging with lived experience participants*   - LEWG Terms of Reference | - N/A |
| 1-week prior | | |
| - Update meeting material based on feedback received from LEWG planning group | - Planning group to provide LEWG members a pre-brief^[[1]](#footnote-1)^ (which includes an agenda, as well as an introduction of the research topic which includes disclaimers of potentially sensitive topics) | - LEWG members to review the pre-brief to prepare questions and feedback |

1. **On the day of the Lived Experience Working Group meeting**

| **YMH Team Researcher** | **LEWG Planning Group** | **LEWG** |
| --- | --- | --- |
| First 30-minutes (pre-brief^1^) | | |
| - N/A | - Planning group to re-introduce the LEWG session without the YMH researcher | - LEWG members to ask questions and prepare feedback |
| During the LEWG session | | |
| - Nominated YMH researcher to engage in an open discussion with the LEWG* | - Planning group to take qualitative notes (e.g., LEWG feedback) - Planning group to facilitate discussion (if necessary) to ensure that time is maximised on open discussion between the YMH researcher and the LEWG | - LEWG members to provide feedback |
| Last 30 minutes (de-brief) | | |
| - N/A | - Planning group to facilitate a de-brief with the LEWG without the YMH researcher | - LEWG to engage in reflection, share honest feedback and discuss solutions openly** |

**Researchers should be mindful to not spend too much time to present their research, but rather, focus on open discussion with the LEWG.*
***The LEWG planning group will ensure that feedback points brought up during the de-brief is delivered in a respectful and constructive manner.*

1. **After the Lived Experience Working Group meeting**

| **YMH Team Researcher** | **LEWG Planning Group** | **LEWG** |
| --- | --- | --- |
| 1-day after | | |
| - Participate in the Qualtrics survey | - Send the YMH researcher with a link to the Qualtrics survey. The survey should be sent to the nominated researcher via the [*ymhtech.admin@sydney.edu.au*](mailto:ymhtech.admin@sydney.edu.au) email address. | - N/A |
| 1-week after | | |
| - Meet with the planning group to give feedback | - Meet with the nominated researcher to go over feedback received from the LEWG - Come up with a plan with the researcher on how the LEWG will be informed of the impact of their contributions - Provide an update to the LEWG on how the YMH researcher plans to incorporate feedback | - Reviews update on how YMH researcher will incorporate LEWG feedback (have the opportunity to ask questions) |
| Approximately* 6-months after | | |
| - ***Before the LEWG meeting:*** Researcher to provide an update on how LEWG input has shaped research (provide via written and/or video form) - ***During the LEWG meeting:*** Researcher invited to attend a LEWG meeting to receive further feedback (at least 30-minutes) - ***After the LEWG meeting***: Participate in the Qualtrics survey | - ***Before the LEWG meeting:*** Provide the YMH researcher’s update (and ensure that this is part of the pre-brief pack going to the LEWG, at least 1-week prior to the next scheduled LEWG meeting) - ***During the LEWG meeting:*** Take qualitative notes - ***After the LEWG meeting***: Send researcher a follow-up survey | - ***Before the LEWG meeting:*** LEWG to review update on Slack, and prepare questions/feedback - ***During the LEWG meeting:*** LEWG to provide further feedback and ask questions during the LEWG session |
| Approximately* 12-months after | | |
| - ***Before the LEWG meeting:*** Researcher to provide an update on how LEWG input has shaped research (provide via written and/or video form) - ***During the LEWG meeting:*** Researcher to attend a LEWG meeting to receive further feedback (at least 30-minutes) - ***After the LEWG meeting***: Participate in the Qualtrics survey | - ***Before the LEWG meeting:*** Provide the YMH researcher’s update (and ensure that this is part of the pre-brief pack going to the LEWG, at least 1-week prior to the next scheduled LEWG meeting) - ***During the LEWG meeting:*** Take qualitative notes - ***After the LEWG meeting***: Send researcher a follow-up survey | - ***Before the LEWG meeting:*** LEWG to review update on Slack, and prepare questions/feedback - ***During the LEWG meeting:*** LEWG to provide further feedback and ask questions during the LEWG session |

**This is an approximate to allow the LEWG planning group to keep track of timelines. Flexibility is critical to take into consideration changes to researcher’s timelines due to various barriers (such as ethics approval, etc).*

1. **Ongoingly engaging with the Lived Experience Working Group**

To ensure that the LEWG is continuously providing input to research conducted by the YMH team, researchers are encouraged to ongoingly engage with the LEWG.

At a minimum, YMH researchers are asked to acknowledge the LEWG in all academic outputs, including but not limited to academic papers, webinars, blog posts and podcasts.

1. **Summary of requirements and time commitments**

| **YMH Team Researcher** | **LEWG Planning Group** | **LEWG** |
| --- | --- | --- |
| Before the LEWG meeting | | |
| - Provide summary and/or meeting material such as slides *(2-3 hours)* - Engage in baseline survey *(15 minutes)* | - Provide oversight to YMH researcher, and give update to LEWG *(2-3 hours)* | - Review pre-brief material *(30 minutes – 1 hour)* |
| During the LEWG meeting | | |
| - Engage in open discussion *(2 hours)* | - Facilitate pre-briefs and de-briefs, and write qualitative notes *(3 hours)* | - Engage in pre-brief and de-brief, and provide feedback *(3 hours)* |
| After the LEWG meeting | | |
| - Complete up to 3 follow-up surveys *(15 minutes each)* - Provide up to two summaries for LEWG *(30 minutes each*) | - Facilitate pre-briefs and de-briefs, and write qualitative notes *(30 minutes)* | - Engage in pre-brief and de-brief, and provide feedback *(30 minutes)* |

1. **Pre-brief** refers to information provided to the LEWG before any engagement with a researcher. [↑](#footnote-ref-1)
