# Supplementary material for: Strengthening mental health research outcomes through genuine partnerships with young people with lived or living experience: A pilot evaluation study
Source: Health Expect. 2023 May 17;26(4):1703–15. doi: 10.1111/hex.13777 (PMC10349217; doi:10.1111/hex.13777)
Supplement: Supplementary file 3 — Supporting Information. [file HEX-26--s003.docx]

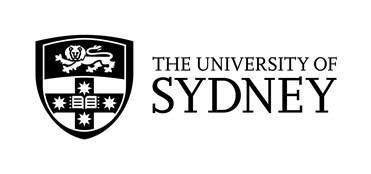


**Youth Partner Baseline Survey**

*Thank you for taking the time to fill out this survey. This research study aims to include the voices of young people with lived experience in mental health within all stages of current and future research studies. This is led by the Youth Mental Health (YMH) & Technology team. Specifically, this study aims to work collaboratively with young people, aged 12 to 30 years old, with lived experience, to improve research and ultimately, improve the way we deliver mental health care to young people.*

*This survey focuses on learning more about your experiences and thoughts on what you hope to achieve from your involvement with the YMH team. We wish to also learn more about your opinion on what changes need to be made to improve future research activities, so that lived experience can play an active role in the YMH team’s research.*

**Personal details**

1. What is your current age?

- 12-16 years old
- 17-20 years old
- 21-25 years old
- 26-30 years old

1. Do you identify as any of the following? *Please select all that apply:*

- Aboriginal and/or Torres Strait Islander descent
- Culturally and Linguistically Diverse background
- Lesbian, gay, bisexual, transgender, gender diverse, intersex and queer (LGBTIQ+)
- Belonging to a religious and/or spiritual group
- None of the above
- Other: Click or tap here to enter text.

**Reasons for participation**

1. Which of the following are your main reasons for participating in this research study? (please select all that apply)

| To help others. |  |
| --- | --- |
| Because someone I know encouraged me to do so.   (*If ticked* – Who encouraged you to participate? Click or tap here to enter text.) |  |
| Because of a positive experience I had participating in another research study. |  |
| To find out more about my own mental health. |  |
| To earn study payment. |  |
| Because of the good reputation of the Brain & Mind Centre. |  |
| I have a friend participating in the study. |  |
| To benefit my overall mental wellbeing. |  |
| Other: Click or tap here to enter text. |  |

**Experience in past research studies**

1. Have you participated in past research studies before?

- Yes 🡪 Go to Q5
- No 🡪 Go to Q8
- Unsure

1. Have you ever participated in research run by the Brain and Mind Centre?

- Yes 🡪 Go to Q6
- No 🡪 Go to Q8
- Unsure

1. Have you ever participated in research run by the YMH team?

- Yes
- No
- Unsure

1. How was your overall experience (e.g. positive, negative, etc)? Please explain. Click or tap here to enter text.

**Expectations & experience in the current research study**

1. Which research activity have you participated in with the YMH team (please select all that apply)?

- Lived Experience Working Group
  - *If ticked –* Which of the following Working Group meetings have you participated in?
    - *List options here*
- Webinar
  - *If ticked –* Which of the following Webinars have you participated in?
    - *List options here*
- Online Survey
  - *If ticked –* Which of the following surveys have you participated in?
    - *List options here*
- Other (such as, but not limited to, workshops, Advisory Group meetings, writing Opinion Pieces with researchers, etc): Click or tap here to enter text.
  - *If ticked -* How many times did you participate in this research activity? Click or tap here to enter text.

1. What are you hoping to gain from participating in this research study? *Please select all that apply:*

- Opportunity for my voice to be heard.
- To make sure that all young people can receive better mental health care.
- To gain a better understanding on what the latest research is saying about youth mental health care.
- Engage with people in the mental health field that I would have otherwise not spoken to.
- To benefit my overall mental wellbeing.
- Other: Click or tap here to enter text.

1. What does genuine inclusion of young people look like in research studies? *Please select all that apply:*

- Respect in the way that young people are treated and spoken to.
- A clear communication process where I feel safe to ask questions and provide feedback.
- Being paid for my time.
- Having enough time to discuss important topics (and not rushing discussions).
- Having diverse participation of young people from all backgrounds (gender, age, sexuality, cultural background, where people live, income/occupation, etc).
- Other: Click or tap here to enter text.

1. In your opinion, why is genuine inclusion of young people important in research studies? Click or tap here to enter text.
2. In your opinion, which of the methods used by the researchers were effective in making you feel **engaged (interested)** in the research topic?

- Presentation (e.g. PowerPoint).
- Webinar.
- Workshops.
- Writing my thoughts and opinions on post-it notes.
- Group discussions.
- Having group meals.
- Going through survey questions.
- Introducing me to different researchers in the YMH team.
- Emails.
- Videos.
- Text messages.
- None of the above.
- Other: Click or tap here to enter text.

1. In your opinion, which of the methods used by the researchers were effective in making you feel **included (contributed)** and/or **empowered (confidence)** to the research topic?

- Presentation (e.g. PowerPoint).
- Webinar.
- Workshops.
- Writing my thoughts and opinions on post-it notes.
- Group discussions.
- Having group meals.
- Going through survey questions.
- Introducing me to different researchers in the YMH team.
- Emails.
- Videos.
- Text messages.
- None of the above.
- Other: Click or tap here to enter text.

1. What are some of the benefits of working with the YMH team? *Please select all that apply:*

- The research team are sensitive to my needs.
- I feel empowered to share my story about my mental health journey.
- I feel safe to share my story about my mental health journey.
- I feel like my contributions are important to the research activity.
- What I contribute has been subsequently put into action.
- I feel confident in supporting someone in their own mental health journey.
- I can see that changes have been made as a direct result of my input.
- None of the above.
- Other: Click or tap here to enter text.

1. What are some things that would improve your experience working with the YMH team at the Brain and Mind Centre?

- Better organisation of research activities.
- Better communication before research activities.
- Less time commitment required from me.
- Better communication of final research learnings.
- More diversity of young people from all backgrounds (gender, age, sexuality, cultural background, where people live, income/occupation, etc).
- None of the above
- Other: Click or tap here to enter text.

1. Thinking about when you participated in the current research study with the YMH team, how much you agree or disagree with the following statements?

| **Statement** | **Strongly Disagree** | **Disagree** | **Neutral** | **Agree** | **Strongly Agree** |
| --- | --- | --- | --- | --- | --- |
| Before providing my consent, I understood what my participation would look like. |  |  |  |  |  |
| The decision to participate was mine. |  |  |  |  |  |
| I feel that I could withdraw at any time. |  |  |  |  |  |
| I understand the risk(s) involved with participating. |  |  |  |  |  |
| I understand the possible benefit(s). |  |  |  |  |  |
| The research staff are happy to answer questions or hear my concerns. |  |  |  |  |  |
| I feel the research staff are professional. |  |  |  |  |  |
| I feel the research staff are knowledgeable. |  |  |  |  |  |
| I am able to participate at a time that works for me. |  |  |  |  |  |
| I appreciate the flexibility of participating. |  |  |  |  |  |
| The variety of tasks helps to keep me engaged. |  |  |  |  |  |
| My overall experience is positive. |  |  |  |  |  |

**Future Participation**

1. Please indicate how much you are in agreement with the following statements in relation to future participation in research studies?

|  | **Very likely** | **Somewhat likely** | **Somewhat unlikely** | **Very unlikely** |
| --- | --- | --- | --- | --- |
| How likely are you to continue participating in the current research study? |  |  |  |  |
| How likely are you to recommend participating in this research study to others? |  |  |  |  |
| How likely are you to participate in future research studies conducted by the YMH team? |  |  |  |  |
| How likely are you to participate in future research studies conducted at other research organisations, excluding the Brain and Mind Centre? |  |  |  |  |
| Has participating in research with the YMH team changed your opinions on participating in future research studies? |  |  |  |  |

1. Is there anything else you would like to comment on to help us improve your overall experience participating in this research study or any future research studies conducted at the Brain and Mind Centre? Click or tap here to enter text.
