# Supplementary material for: Strengthening mental health research outcomes through genuine partnerships with young people with lived or living experience: A pilot evaluation study
Source: Health Expect. 2023 May 17;26(4):1703–15. doi: 10.1111/hex.13777 (PMC10349217; doi:10.1111/hex.13777)
Supplement: Supplementary file 4 — Supporting information. [file HEX-26--s004.docx]

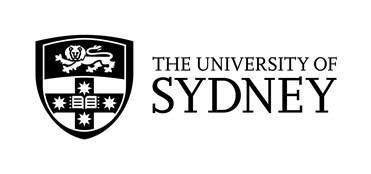


**Academic Researcher Survey**

*Thank you for taking the time to fill out this survey. This research study aims to embed the voices of young people with lived mental health experience within current and future research studies led by the Youth Mental Health & Technology team. Specifically, this study aims to work collaboratively with young people, aged 12 to 30 years old, with lived experience to improve research design, development, implementation and evaluation, to ultimately, improve the way mental health care is delivered to young people.*

*This survey focuses on learning more about* *how we can improve future efforts and make sure that your research outcomes can maximise on lived experience input.*

**Personal details**

1. What is your current age?

- 18-24 years old
- 25-34 years old
- 35-44 years old
- 45-54 years old
- 55-64 years old
- 65 years or older

1. Do you identify as any of the following? *Please select all that apply:*

- Aboriginal and/or Torres Strait Islander descent
- Culturally and Linguistically Diverse background
- Lesbian, gay, bisexual, transgender, gender diverse, intersex and queer (LGBTIQ+)
- Belonging to a religious and/or spiritual group
- None of the above
- Other: Click or tap here to enter text.

1. What is the highest level of education you have completed?

- Bachelor’s degree or less
- Master’s degree
- PhD or equivalent
- Other: Click or tap here to enter text.

1. What is your primary position at the Brain and Mind Centre?

- Professional research support (such as Research Assistant/Officer/Manager)
- Honour’s student
- Higher degree research (Master’s or PhD) student or equivalent
- Postdoctoral Research Associate or equivalent (Level A)
- Postdoctoral Research Fellow or equivalent (Level B)
- Senior Postdoctoral Research Fellow or equivalent (Level C)
- Associate Professor or equivalent (Level D)
- Professor or equivalent (Level E)
- Other: Click or tap here to enter text.

1. How long have you been working in youth mental health research? Click or tap here to enter text.
2. How did you get into the field of youth mental health research? Click or tap here to enter text.
3. What is your area of youth mental health research primarily concerned with? *Please select all that apply:*

- Clinical research (including clinical trials)
- Co-design of interventions
- Implementation of interventions
- Evaluation
- Heath policy
- Other: Click or tap here to enter text.

**Lived experience input in research**

1. Have you had previous experience working with young people with lived experience in your research?

- Yes
- No
- Unsure
- Other: Click or tap here to enter text.

1. *[If ticked ‘Yes’ or ‘Unsure’ for Q8]* Are you currently engaged with lived experience participants in your research?

- Yes
- No
- Unsure
- Other: Click or tap here to enter text.

1. *[ If ticked ‘Yes’ or ‘Unsure’ for Q8]* Is (or was) funding available for your involvement working with young people with lived experience?

- Yes

*If ticked:* What is (or was) the source of funding? Click or tap here to enter text.

- No
- Unsure
- Other: Click or tap here to enter text.

1. *[If ticked ‘Yes’ or ‘Unsure’ for Q8]* Does your supervisor and/or line manager undertake work with young people with lived experience?

- Yes
- No
- Unsure
- Other: Click or tap here to enter text.

1. *[If ticked ‘Yes’ or ‘Unsure’ for Q8]* How often are young people with lived experience involved in your research?

- Never
- Rarely (e.g., once a year)
- Sometimes (e.g., every 6 months)
- Often (e.g., every 3 months)
- Always (e.g., every month)
- Other: Click or tap here to enter text.

1. *[If ticked ‘Yes’ or ‘Unsure’ for Q8]* At which research stage(s) have you included young people with lived experience in your research? *Please select all that apply:*

- Identifying the research topic
- Seeking funding
- Designing the research project (such as input into recruitment strategies)
- Managing the research project (such as a steering/advisory group)
- Conducting the research (such as collection of data)
- Analysing and interpreting findings
- Dissemination (such as co-publishing or co-presenting at conferences)
- Evaluation
- Acting on the findings
- Other: Click or tap here to enter text.

1. *[If ticked ‘No’ for Q8]* At which research stage(s) do you think it would be beneficial to include young people with lived experience in your research? *Please select all that apply:*

- Identifying the research topic
- Seeking funding
- Designing the research project (such as input into recruitment strategies)
- Managing the research project (such as a steering/advisory group)
- Conducting the research (such as collection of data)
- Analysing and interpreting findings
- Dissemination (such as co-publishing or co-presenting at conferences)
- Evaluation
- Acting on the findings
- Other: Click or tap here to enter text.

1. *[If ticked ‘Yes’ or ‘Unsure’ for Q8]* What is the extent young people with lived experience are currently (or have previously) engaged in your research? *Please select all that apply*:

- Young people are/were engaged, but their contributions are/were minimal
- Young people are/were informed about the project, but do/did not contribute to its design/operationalisation
- Young people provide(d) consultation on specific project components
- Young people are/were partners on projects designed and developed by researchers
- Young people lead/led projects and initiate(d) project action
- Young people and researchers equally share(d) decision-making responsibilities
- Other: Click or tap here to enter text.

1. Which of the following are your (or would be your) main reasons for seeking lived experience input in your research? *Please select all that apply:*

- To improve the chances that my study will succeed (such as successful implementation of study design).
- Because someone encouraged me to seek lived experience input.
- *If ticked*: Who encouraged you to seek lived experience input (e.g., colleague, supervisor, etc)? Click or tap here to enter text.
- To collect qualitative data.
- To publish academic papers.
- To validate the quantitative data that I am collecting by adding lived experience perspectives.
- To advocate better mental health care/outcomes for young people through my research.
- To ensure research outcomes, policy and/or practice can be informed by lived experience input.
- Other: Click or tap here to enter text.

**Current perspectives**

1. How confident do you feel engaging with young people with lived experience in your research?

| 1 (not confident at all) |  |  |  |  |  |  |  |  | 10 (extremely confident) |
| --- | --- | --- | --- | --- | --- | --- | --- | --- | --- |

1. How much do you agree with the following statement: Lived experience input has a direct positive impact on research outcomes.

| 1 (completely disagree) |  |  |  |  |  |  |  |  | 10 (completely agree) |
| --- | --- | --- | --- | --- | --- | --- | --- | --- | --- |

1. In your opinion, how valuable is it to engage with young people with lived experience in your research?

| 1 (not valuable at all) |  |  |  |  |  |  |  |  | 10 (extremely valuable) |
| --- | --- | --- | --- | --- | --- | --- | --- | --- | --- |

1. How do you think inclusion of lived experience will (or could potentially) add value to your research? If there are none, please answer ‘N/A.’ Click or tap here to enter text.
2. How important are the following factors to ensure the genuine inclusion of young people with lived experience in research studies?

|  | **Not at all important** | **Low importance** | **Slightly important** | **Neutral** | **Moderately important** | **Very important** | **Extremely important** |
| --- | --- | --- | --- | --- | --- | --- | --- |
| Respect in the way that young people are treated and spoken to. |  |  |  |  |  |  |  |
| A clear communication process where young people feel safe to ask questions and provide feedback. |  |  |  |  |  |  |  |
| Paying young people for their time in participating. |  |  |  |  |  |  |  |
| Allocating enough time to discuss important topics and not rushing discussions. |  |  |  |  |  |  |  |
| Having diverse participation of young people from all backgrounds (gender, age, sexuality, cultural background, where people live, income/occupation, etc). |  |  |  |  |  |  |  |
| Other: Click or tap here to enter text. |  |  |  |  |  |  |  |

1. In your opinion, which of the methods have you found to be effective in **engaging** young people with lived experience in your research study? *Please select all that apply:*

- Presentation (e.g. PowerPoint).
- Webinar.
- Interactive activities (such as getting young people to write their thoughts and opinions on post-it notes) that capture the thoughts and experiences of young people about a specific topic.
- Workshops.
- Group discussions.
- Having group meals.
- Online surveys.
- Networking opportunities.
- None of the above.
- Other: Click or tap here to enter text.

**Future planning**

1. How can researchers be encouraged to work with lived experience participants?

- Having recognised research outputs for time spent collaborating with lived experience participants (e.g. altmetrics, publications, presentations, etc).
- Having opportunities to engage with lived experience participants to facilitate dissemination of research to a wider audience (e.g. webinar, podcast).
- Research funding to support activities that include lived experience participants.
- Professional development opportunities to learn how to appropriately work with lived experience participants.
- Better communication from my research team on opportunities to work with lived experience participants.
- Less time commitment required from researchers.
- None of the above – working with lived experience is not important for my research
- Other: Click or tap here to enter text.

1. To what extent do you think the following are barriers to include lived experience input in your research?

|  | **Not a barrier at all** | **Somewhat of a barrier (e.g., there are challenges but lived experience inclusion is still possible)** | **Neither a barrier nor a facilitator** | **Extreme barrier (e.g., prevents the inclusion of lived experience)** |
| --- | --- | --- | --- | --- |
| Research funding. |  |  |  |  |
| Not enough time. |  |  |  |  |
| Not enough resources (including human resources). |  |  |  |  |
| Not a high priority for my research outcomes. |  |  |  |  |
| Concerns about my ability to engage or communicate effectively with young people with lived experience. |  |  |  |  |
| Not always clear how lived experience input will improve my research outcomes. |  |  |  |  |
| Not always clear how to engage young people with lived experience on a practical level. |  |  |  |  |
| Not always possible to include young people with lived experience. |  |  |  |  |
| Lack of support because my ORGANISATION does not recognise the value of this type of work. |  |  |  |  |
| Lack of support because my TEAM does not recognise the value of this type of work. |  |  |  |  |
| Other (please specify): |  |  |  |  |

1. What are some ways researchers can improve the experiences of young people with lived experience when engaged in research?

- Inclusion of lived experience input from the design stage of research planning (e.g. when submitting grant applications).
- *If ticked:* Is this realistic? Please explain. Click or tap here to enter text.
- Clear communication to young people about how their contributions have led to research outcomes.
- *If ticked:* Is this realistic? Please explain. Click or tap here to enter text.
- Better organisation (e.g. administratively) of research activities.
- *If ticked:* Is this realistic? Please explain. Click or tap here to enter text.
- Inclusion of young people from diverse backgrounds (socioeconomic, cultural, gender, sexuality, etc).
- *If ticked:* Is this realistic? Please explain. Click or tap here to enter text.
- None of the above
- Other: Click or tap here to enter text.

1. Is there anything else you would like to comment on how research infrastructure (such as more administrative support, positive workplace culture, etc) can improve to support researchers in engaging with lived experience participants? Click or tap here to enter text.Click or tap here to enter text.

*Thank you so much for completing this survey.*
