# Supplementary material for: Strengthening mental health research outcomes through genuine partnerships with young people with lived or living experience: A pilot evaluation study
Source: Health Expect. 2023 May 17;26(4):1703–15. doi: 10.1111/hex.13777 (PMC10349217; doi:10.1111/hex.13777)
Supplement: Supplementary file 5 — Supporting Information. [file HEX-26--s005.pptx]

## Slide 1
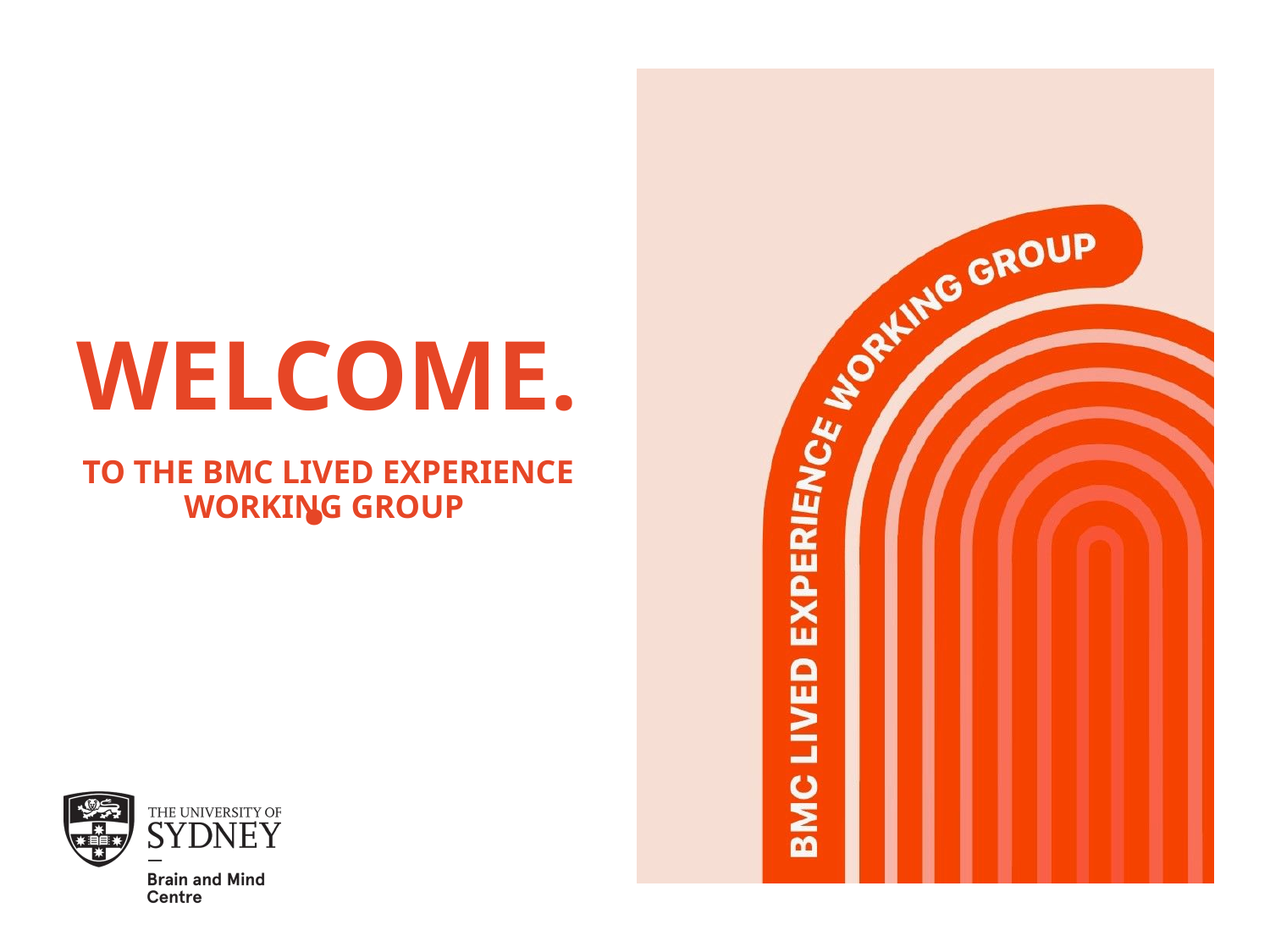

# WELCOME..
TO THE BMC LIVED EXPERIENCE WORKING GROUP

## Slide 2
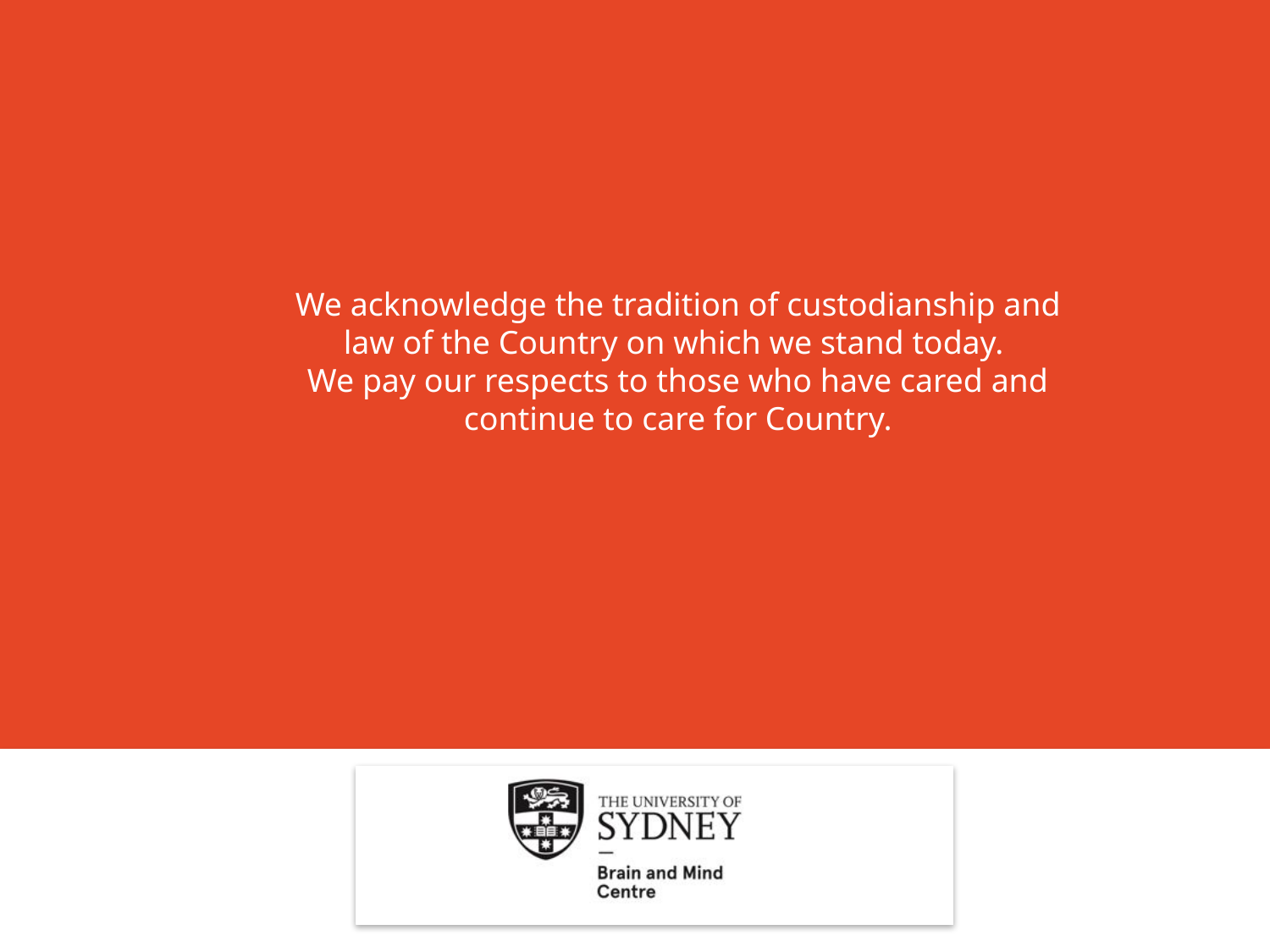

## Slide 3
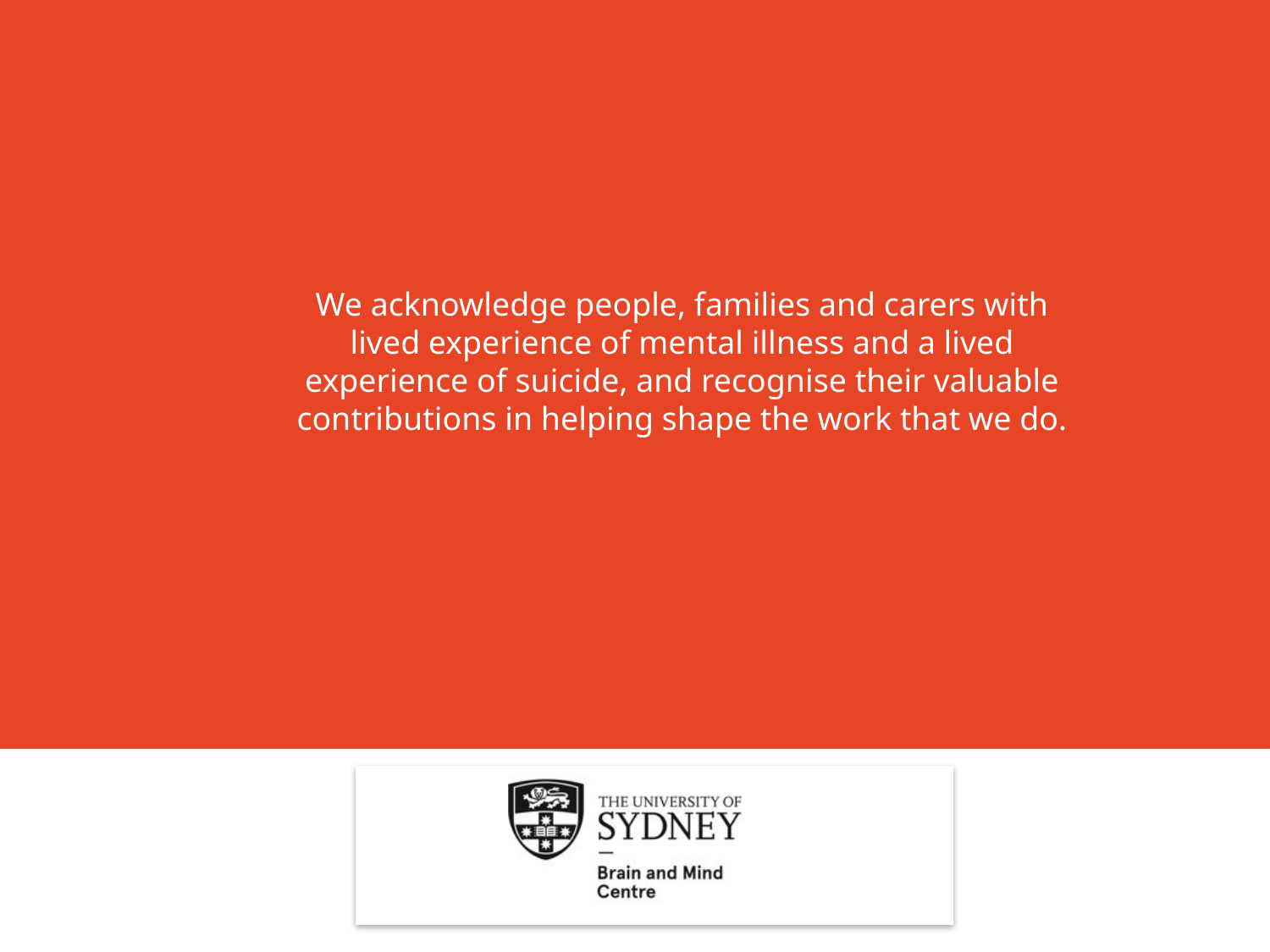

## Slide 4
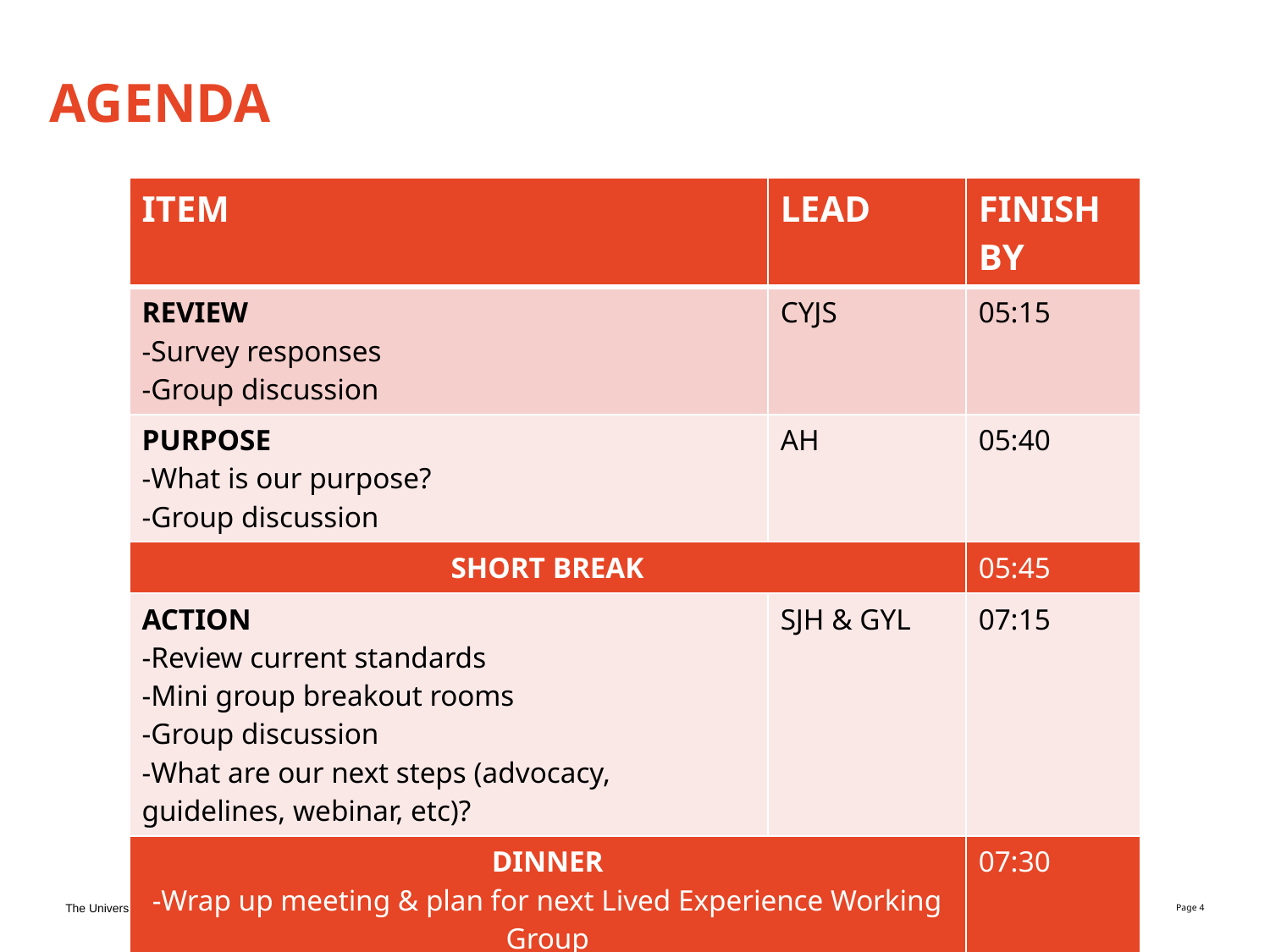

# AGENDA
| ITEM | LEAD | FINISH BY |
| --- | --- | --- |
| REVIEW -Survey responses -Group discussion | CYJS | 05:15 |
| PURPOSE -What is our purpose? -Group discussion | AH | 05:40 |
| SHORT BREAK | | 05:45 |
| ACTION -Review current standards -Mini group breakout rooms -Group discussion -What are our next steps (advocacy, guidelines, webinar, etc)? | SJH & GYL | 07:15 |
| DINNER -Wrap up meeting & plan for next Lived Experience Working Group | | 07:30 |

## Slide 5
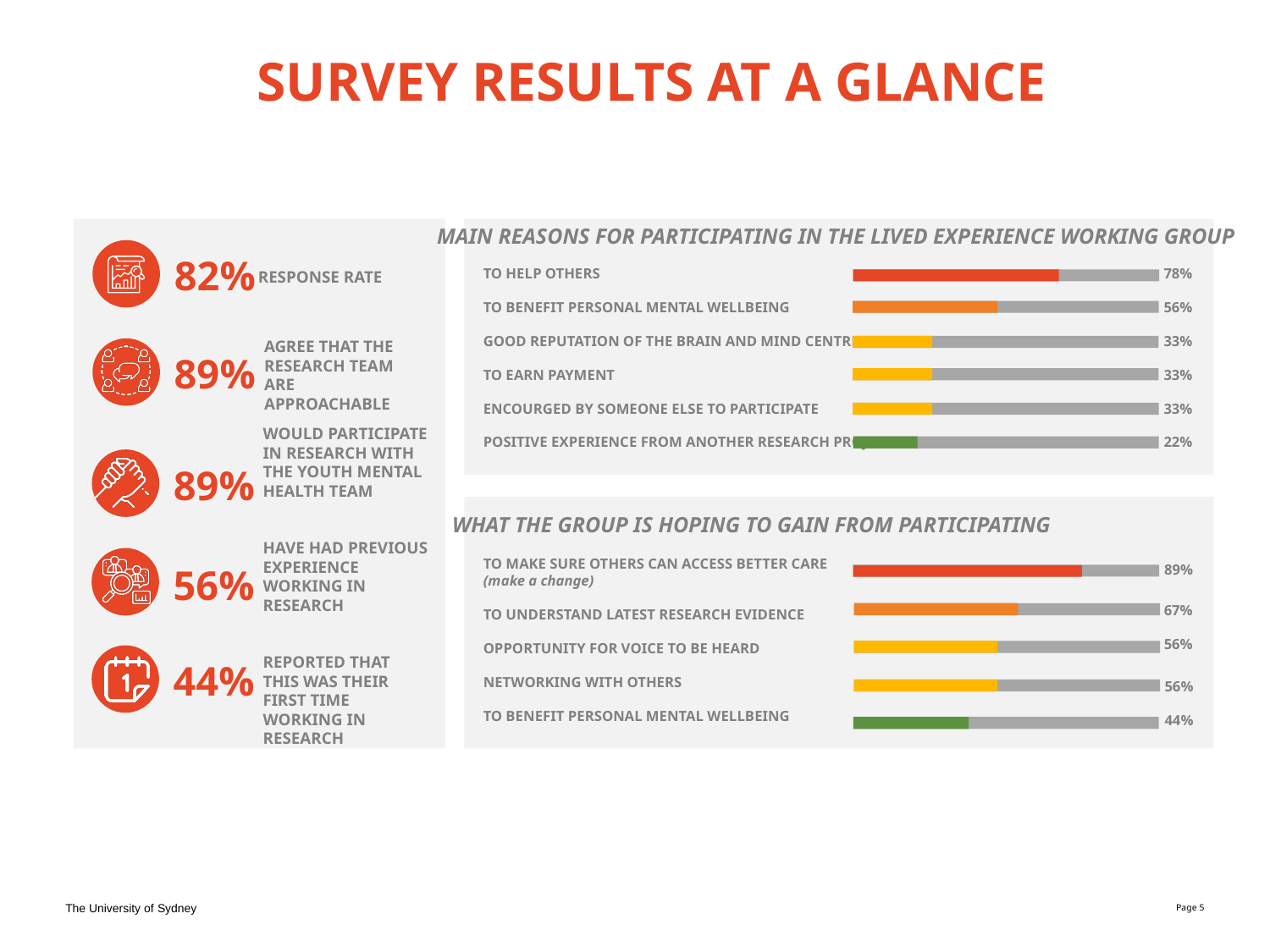

SURVEY RESULTS AT A GLANCE
MAIN REASONS FOR PARTICIPATING IN THE LIVED EXPERIENCE WORKING GROUP
82%
TO HELP OTHERS
TO BENEFIT PERSONAL MENTAL WELLBEING
GOOD REPUTATION OF THE BRAIN AND MIND CENTRE
TO EARN PAYMENT
ENCOURGED BY SOMEONE ELSE TO PARTICIPATE
POSITIVE EXPERIENCE FROM ANOTHER RESEARCH PROJECT
78%
56%
33%
33%
33%
22%
RESPONSE RATE
AGREE THAT THE RESEARCH TEAM ARE APPROACHABLE
89%
WOULD PARTICIPATE IN RESEARCH WITH THE YOUTH MENTAL HEALTH TEAM
HAVE HAD PREVIOUS EXPERIENCE WORKING IN RESEARCH
REPORTED THAT THIS WAS THEIR FIRST TIME WORKING IN RESEARCH
89%
WHAT THE GROUP IS HOPING TO GAIN FROM PARTICIPATING
COMMENTS
FILES
TO MAKE SURE OTHERS CAN ACCESS BETTER CARE
(make a change)
TO UNDERSTAND LATEST RESEARCH EVIDENCE
OPPORTUNITY FOR VOICE TO BE HEARD
NETWORKING WITH OTHERS
TO BENEFIT PERSONAL MENTAL WELLBEING
56%
44%
89%
67%
56%
56%
44%

## Slide 6
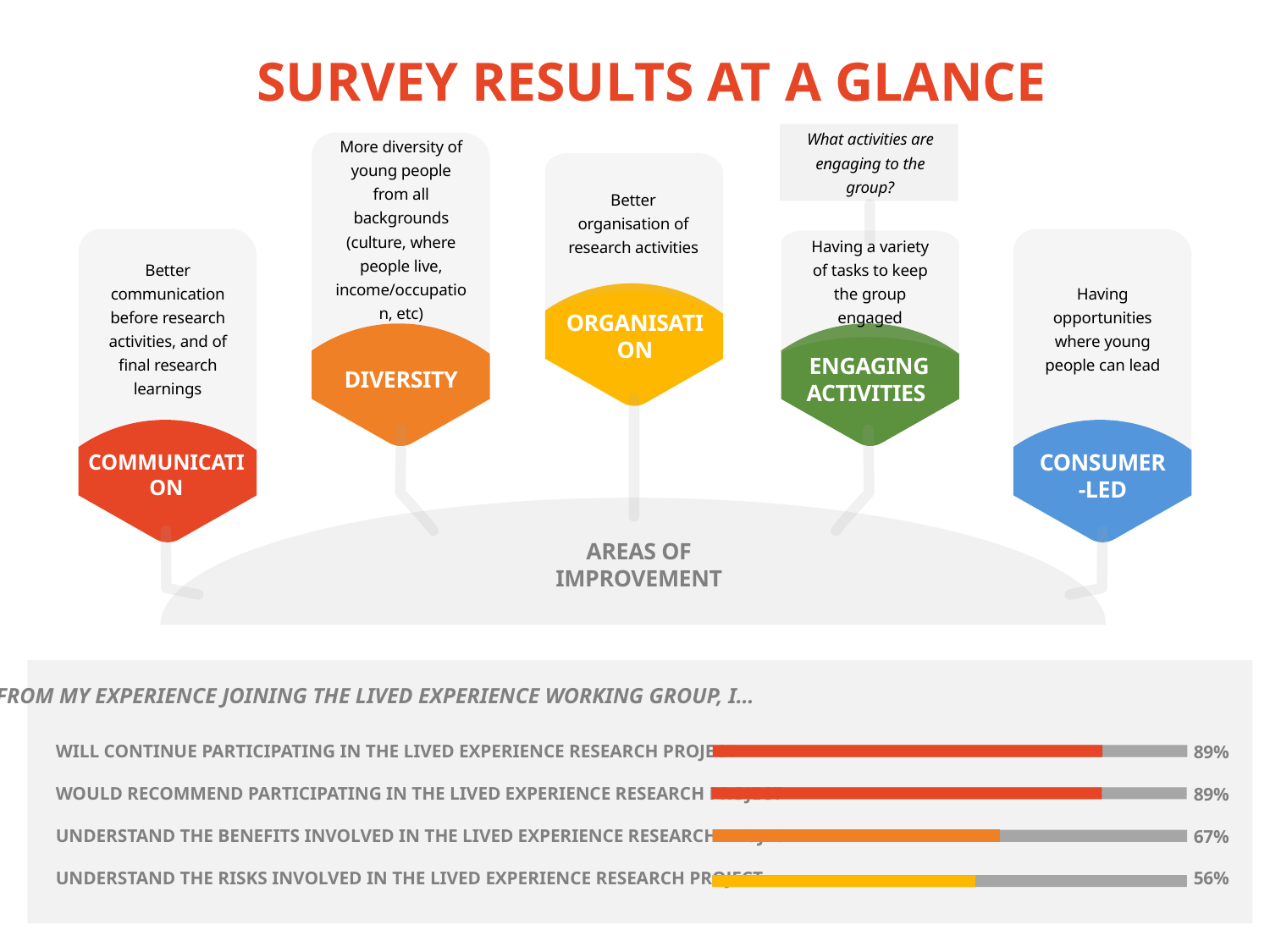

SURVEY RESULTS AT A GLANCE
What activities are engaging to the group?
More diversity of young people from all backgrounds (culture, where people live, income/occupation, etc)
Better organisation of research activities
Having a variety of tasks to keep the group engaged
Better communication before research activities, and of final research learnings
Having opportunities where young people can lead
ORGANISATION
ENGAGING ACTIVITIES
DIVERSITY
CONSUMER-LED
COMMUNICATION
AREAS OF IMPROVEMENT
FROM MY EXPERIENCE JOINING THE LIVED EXPERIENCE WORKING GROUP, I…
WILL CONTINUE PARTICIPATING IN THE LIVED EXPERIENCE RESEARCH PROJECT
WOULD RECOMMEND PARTICIPATING IN THE LIVED EXPERIENCE RESEARCH PROJECT
UNDERSTAND THE BENEFITS INVOLVED IN THE LIVED EXPERIENCE RESEARCH PROJECT
UNDERSTAND THE RISKS INVOLVED IN THE LIVED EXPERIENCE RESEARCH PROJECT
89%
89%
67%
56%

## Slide 7
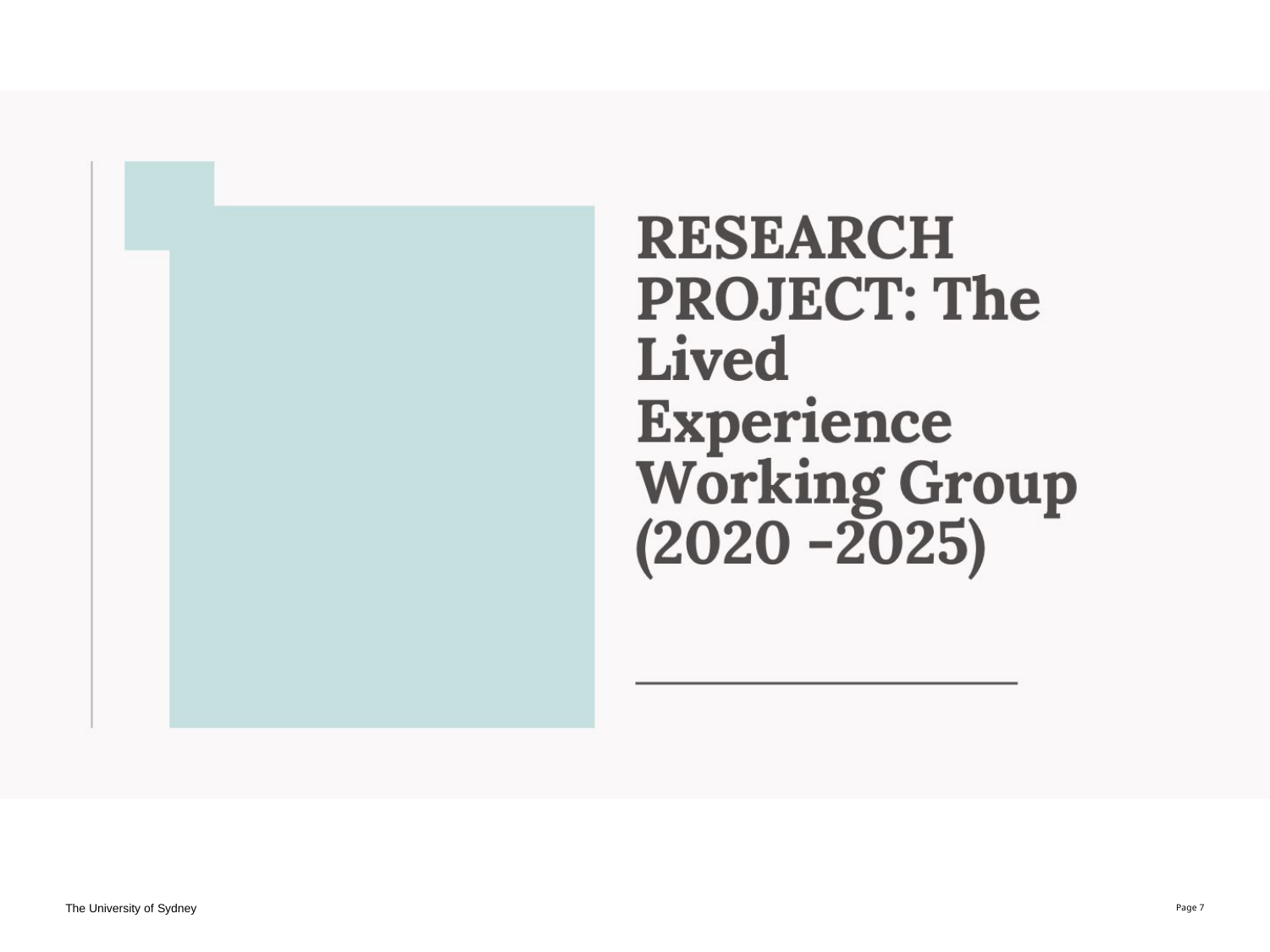

## Slide 8
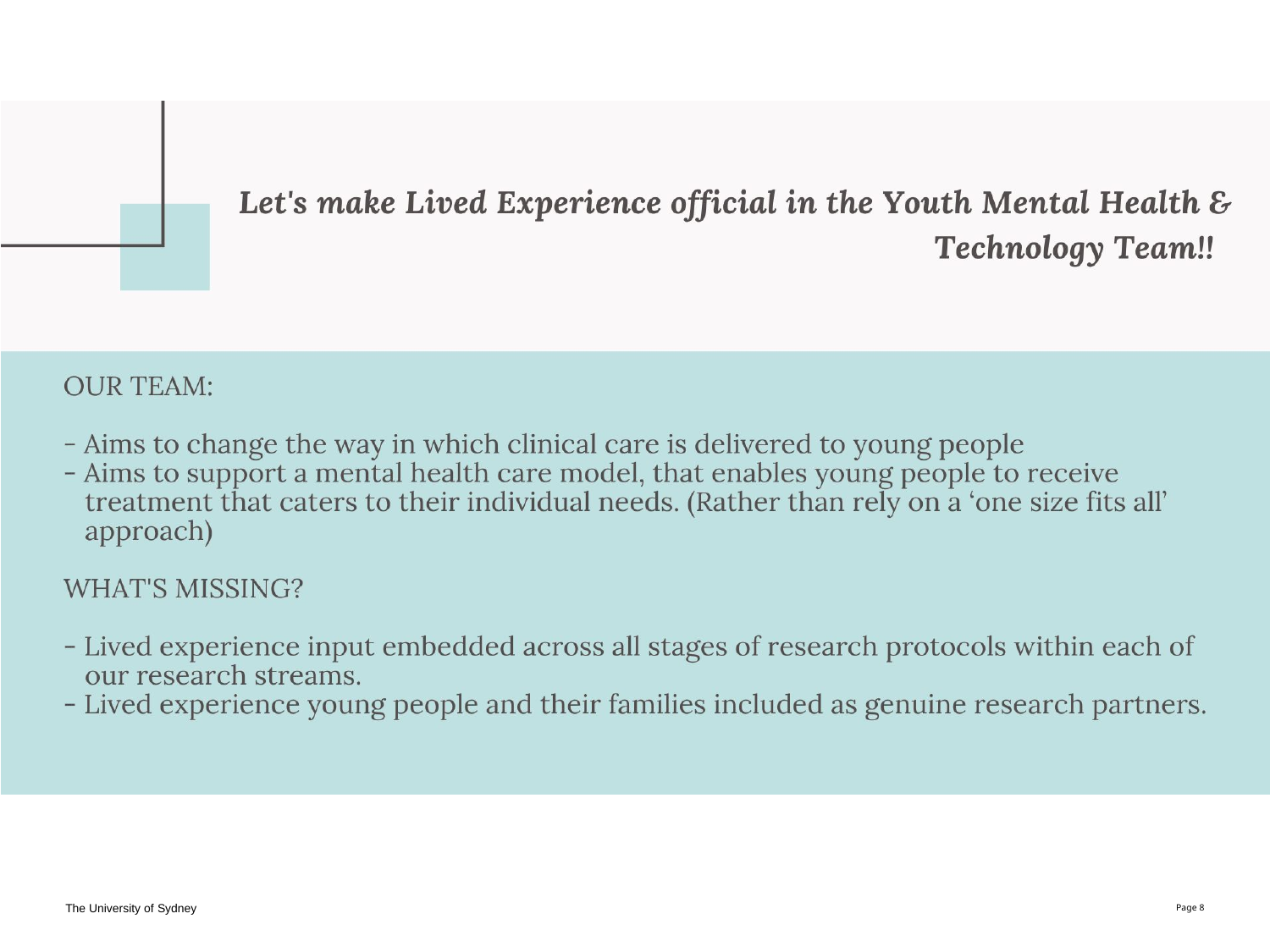

## Slide 9
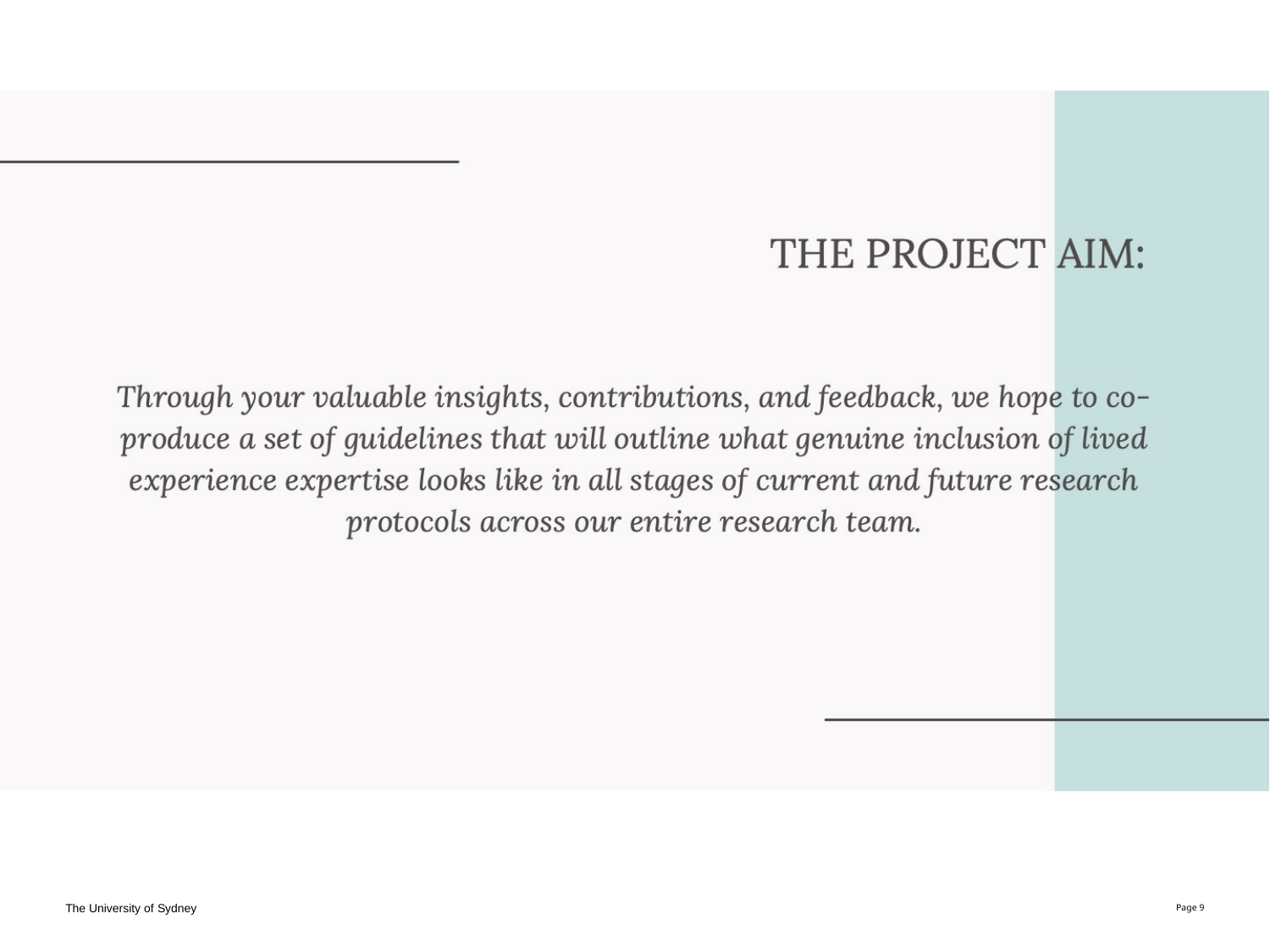

## Slide 10
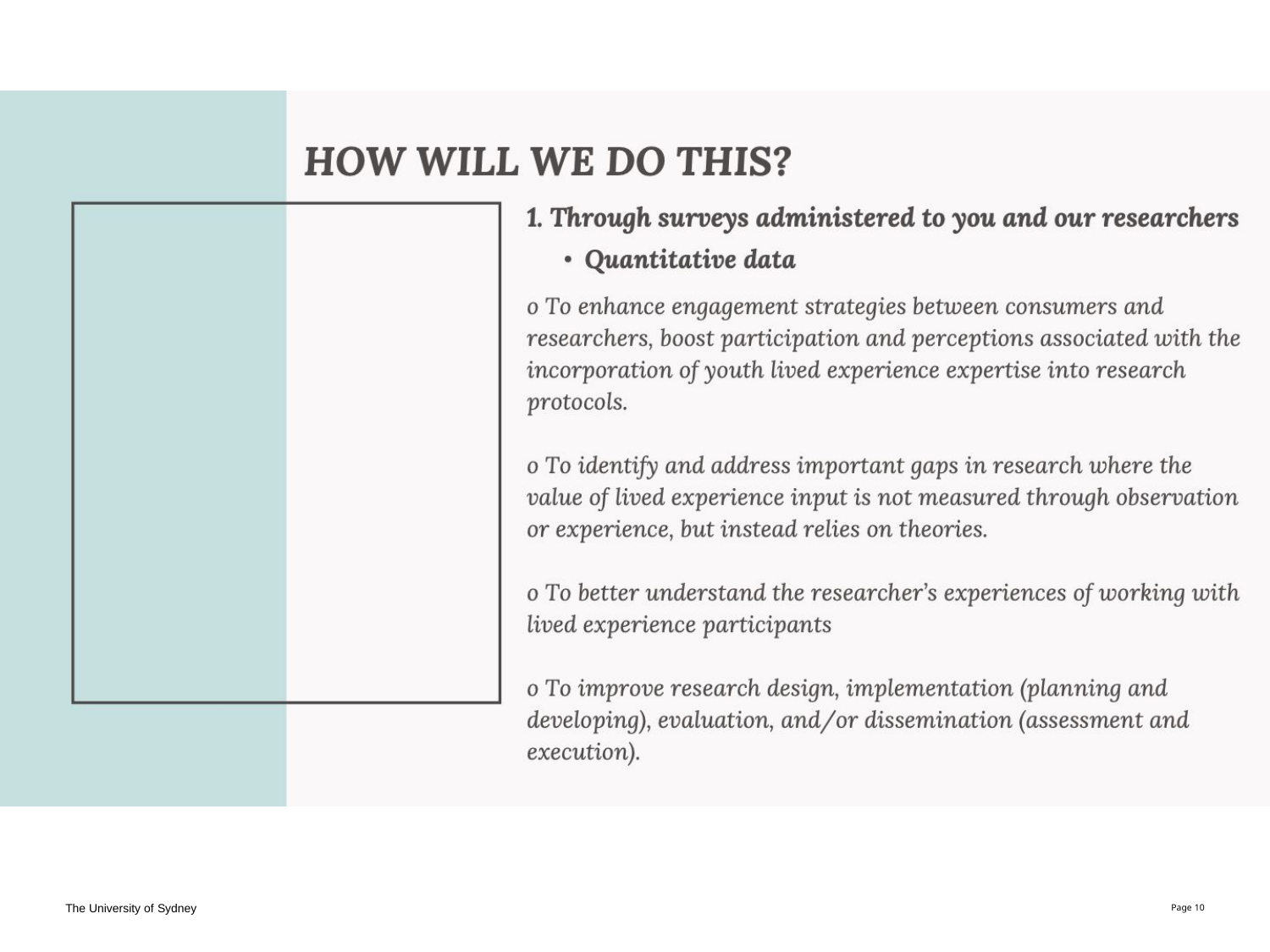

## Slide 11
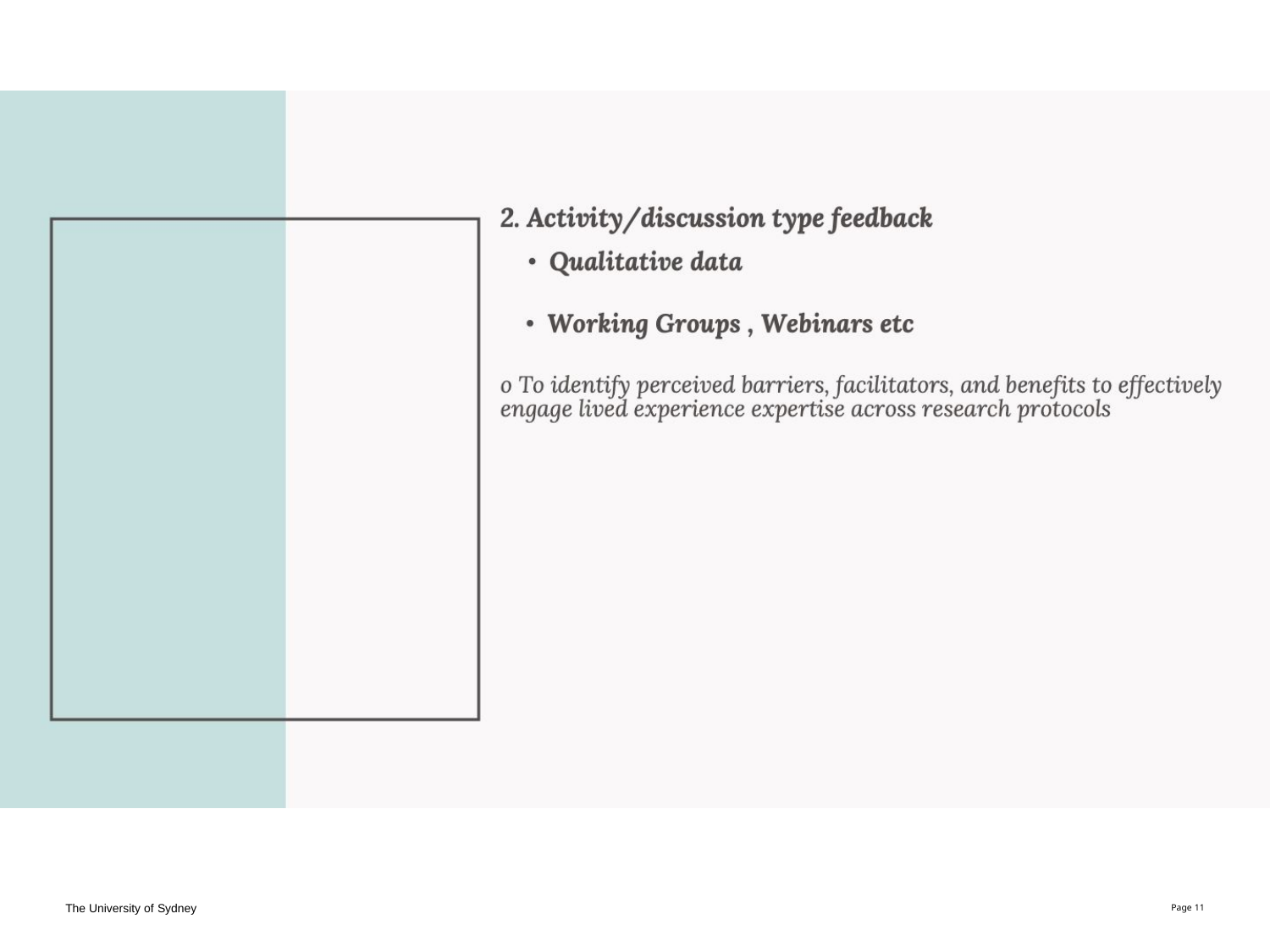

## Slide 12
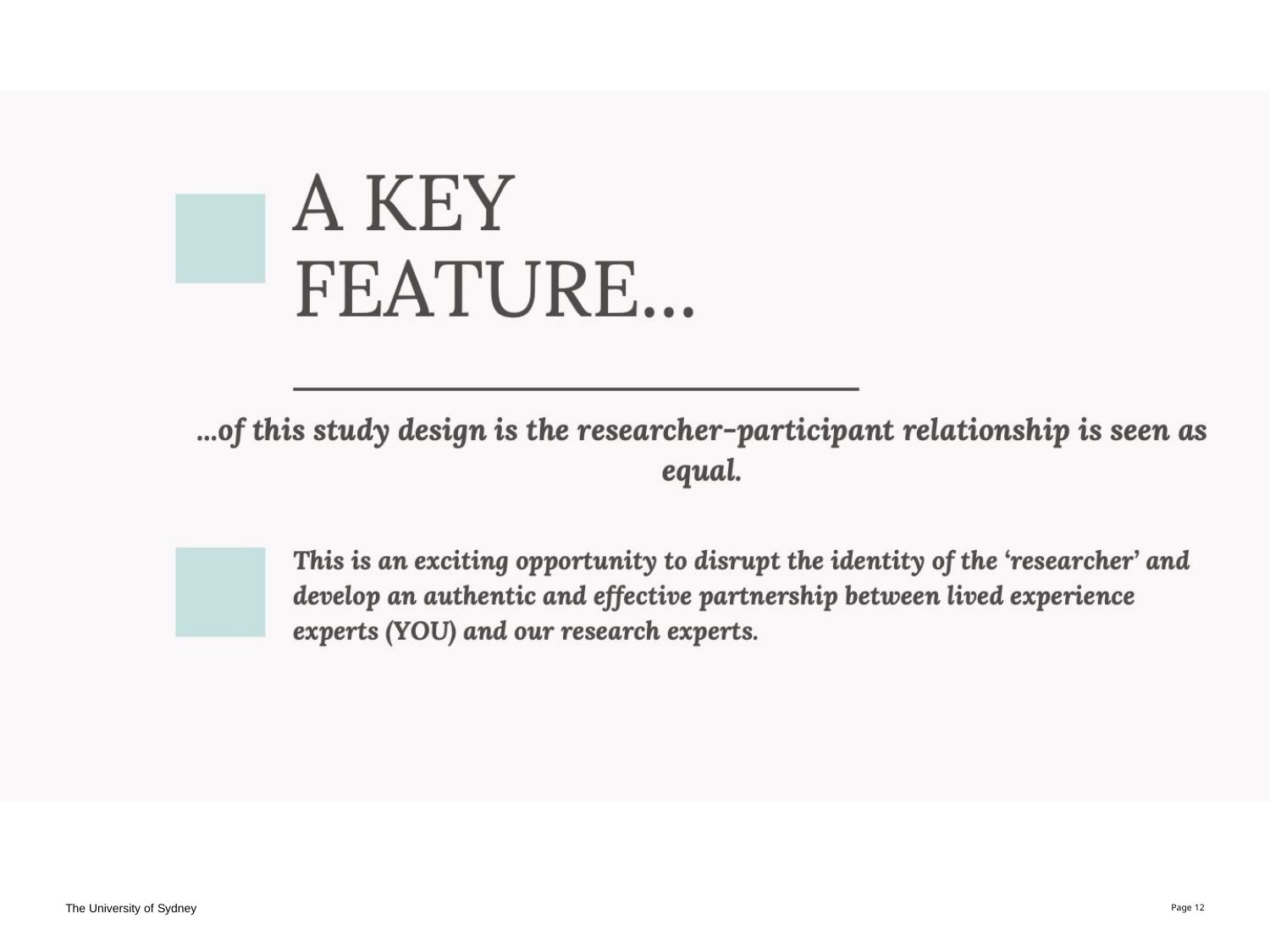

## Slide 13
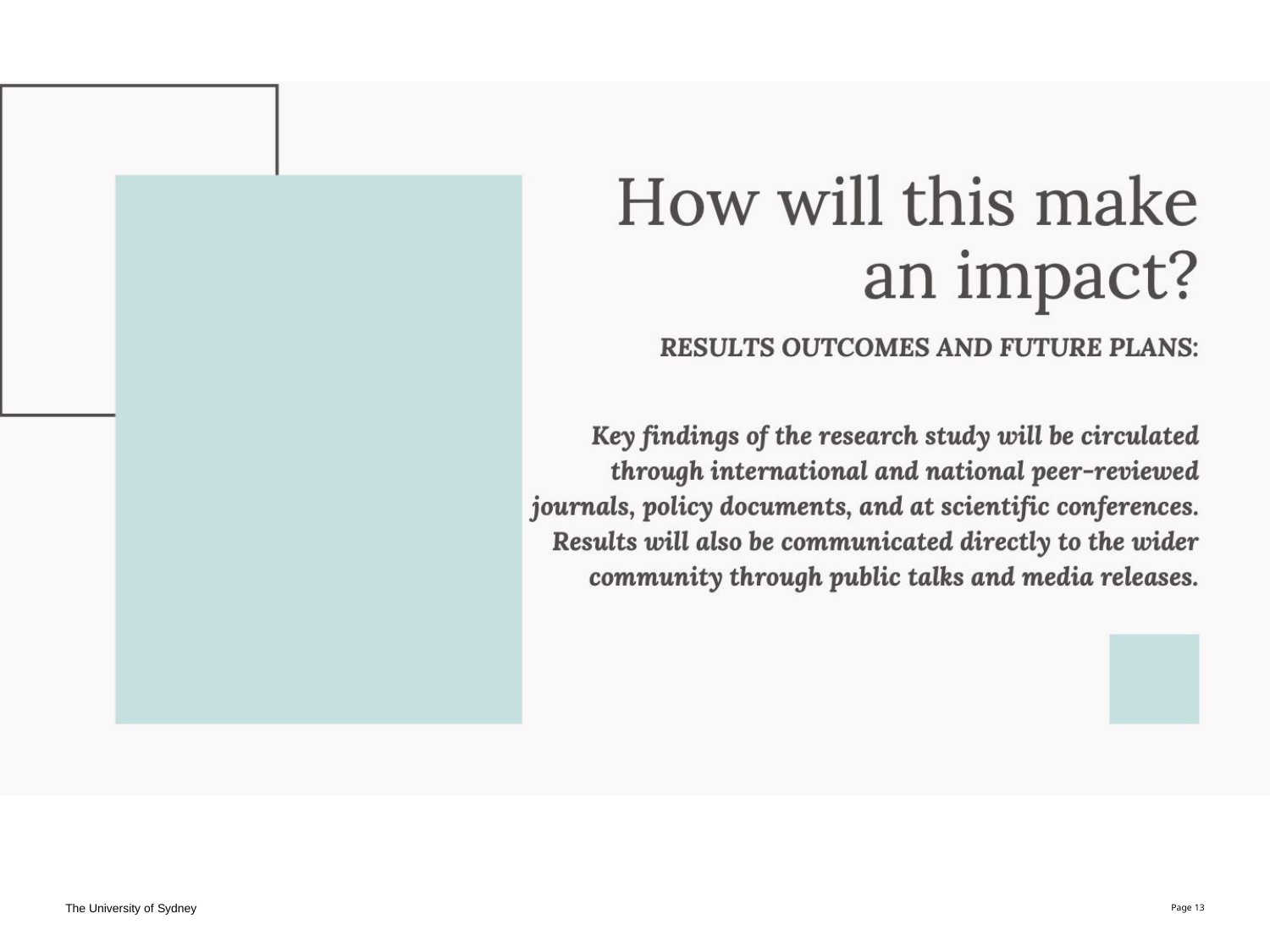

## Slide 14
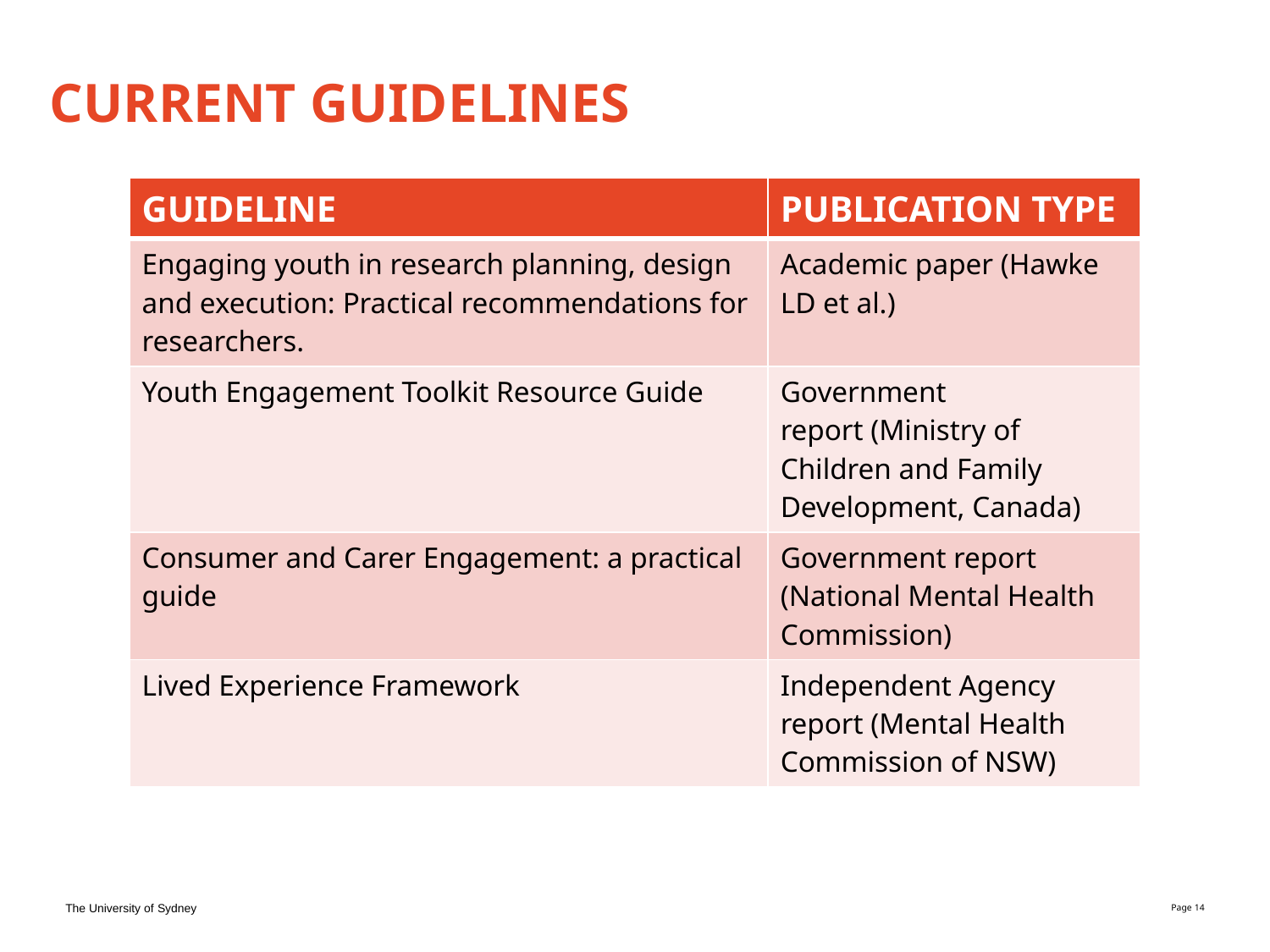

# CURRENT GUIDELINES
| GUIDELINE | PUBLICATION TYPE |
| --- | --- |
| Engaging youth in research planning, design and execution: Practical recommendations for researchers. | Academic paper (Hawke LD et al.) |
| Youth Engagement Toolkit Resource Guide | Government report (Ministry of Children and Family Development, Canada) |
| Consumer and Carer Engagement: a practical guide | Government report (National Mental Health Commission) |
| Lived Experience Framework | Independent Agency report (Mental Health Commission of NSW) |

## Slide 15
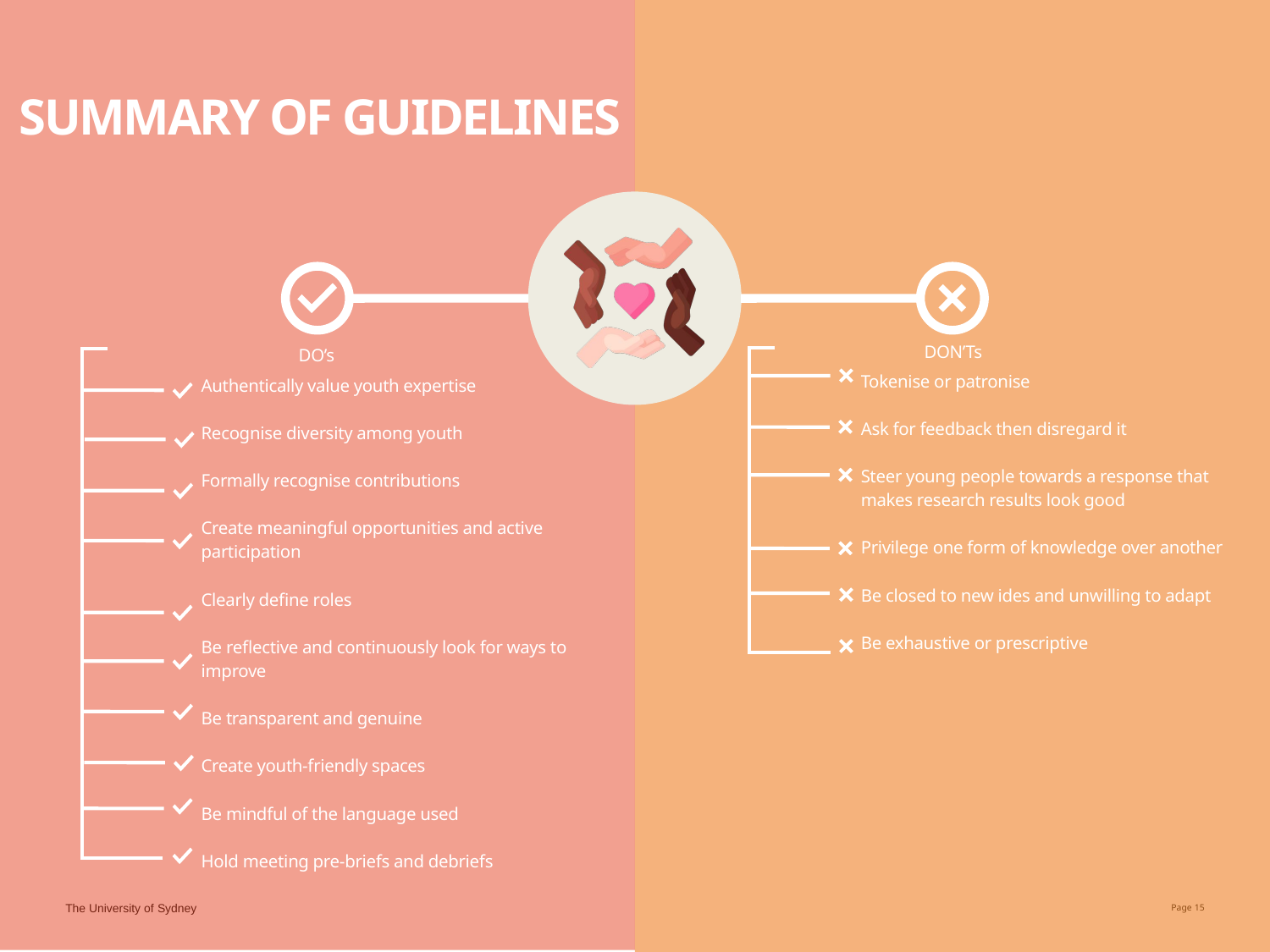

SUMMARY OF GUIDELINES
DON’Ts
DO’s
Tokenise or patronise
Ask for feedback then disregard it
Steer young people towards a response that makes research results look good
Privilege one form of knowledge over another
Be closed to new ides and unwilling to adapt
Be exhaustive or prescriptive
Authentically value youth expertise
Recognise diversity among youth
Formally recognise contributions
Create meaningful opportunities and active participation
Clearly define roles
Be reflective and continuously look for ways to improve
Be transparent and genuine
Create youth-friendly spaces
Be mindful of the language used
Hold meeting pre-briefs and debriefs

## Slide 16
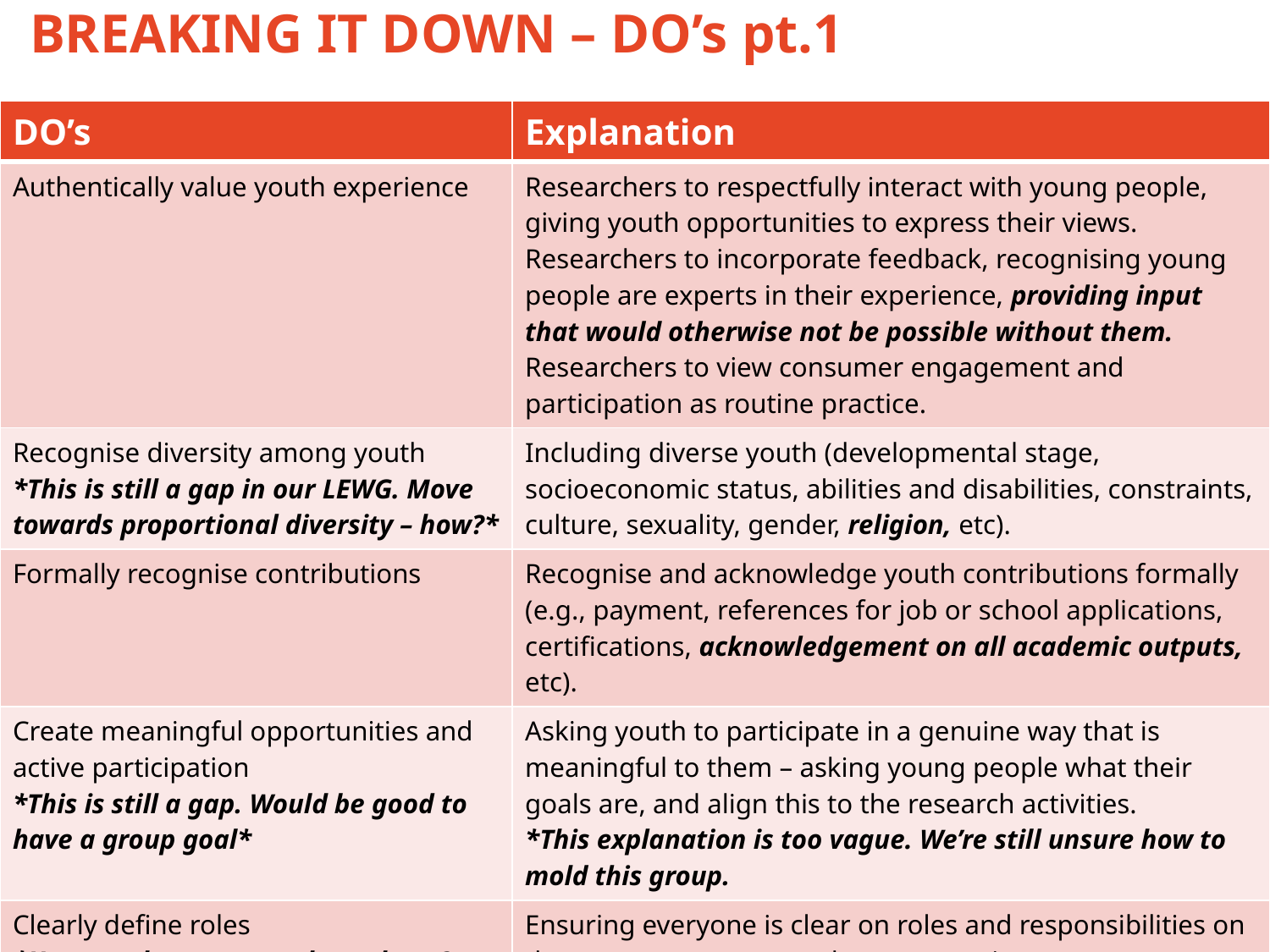

# BREAKING IT DOWN – DO’s pt.1
| DO’s | Explanation |
| --- | --- |
| Authentically value youth experience | Researchers to respectfully interact with young people, giving youth opportunities to express their views. Researchers to incorporate feedback, recognising young people are experts in their experience, providing input that would otherwise not be possible without them. Researchers to view consumer engagement and participation as routine practice. |
| Recognise diversity among youth \*This is still a gap in our LEWG. Move towards proportional diversity – how?\* | Including diverse youth (developmental stage, socioeconomic status, abilities and disabilities, constraints, culture, sexuality, gender, religion, etc). |
| Formally recognise contributions | Recognise and acknowledge youth contributions formally (e.g., payment, references for job or school applications, certifications, acknowledgement on all academic outputs, etc). |
| Create meaningful opportunities and active participation \*This is still a gap. Would be good to have a group goal\* | Asking youth to participate in a genuine way that is meaningful to them – asking young people what their goals are, and align this to the research activities. \*This explanation is too vague. We’re still unsure how to mold this group. |
| Clearly define roles \*How much autonomy do we have? What are we trying to achieve? | Ensuring everyone is clear on roles and responsibilities on the team, to prevent unclear expectations. \*Not defining roles brings a sense of pointlessness. |
| Be reflective and look for opportunities to improve | Adopt shared, safe, empathetic, inclusive and curious evaluation approaches, committing to continual improvement. |

## Slide 17
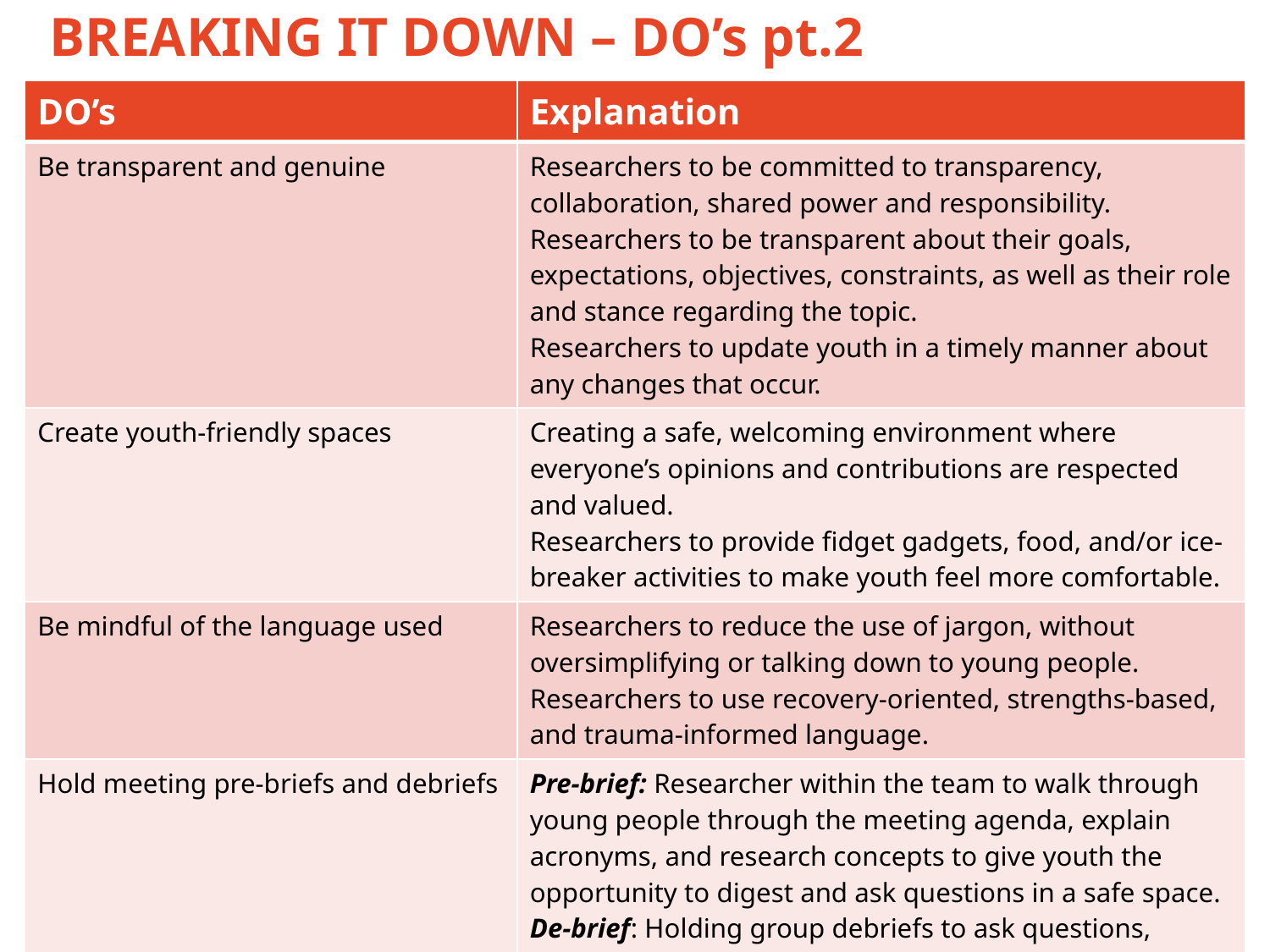

# BREAKING IT DOWN – DO’s pt.2
| DO’s | Explanation |
| --- | --- |
| Be transparent and genuine | Researchers to be committed to transparency, collaboration, shared power and responsibility. Researchers to be transparent about their goals, expectations, objectives, constraints, as well as their role and stance regarding the topic. Researchers to update youth in a timely manner about any changes that occur. |
| Create youth-friendly spaces | Creating a safe, welcoming environment where everyone’s opinions and contributions are respected and valued. Researchers to provide fidget gadgets, food, and/or ice-breaker activities to make youth feel more comfortable. |
| Be mindful of the language used | Researchers to reduce the use of jargon, without oversimplifying or talking down to young people. Researchers to use recovery-oriented, strengths-based, and trauma-informed language. |
| Hold meeting pre-briefs and debriefs | Pre-brief: Researcher within the team to walk through young people through the meeting agenda, explain acronyms, and research concepts to give youth the opportunity to digest and ask questions in a safe space. De-brief: Holding group debriefs to ask questions, clarify issues, reflect on contributions, and plan for future meetings. |

## Slide 18
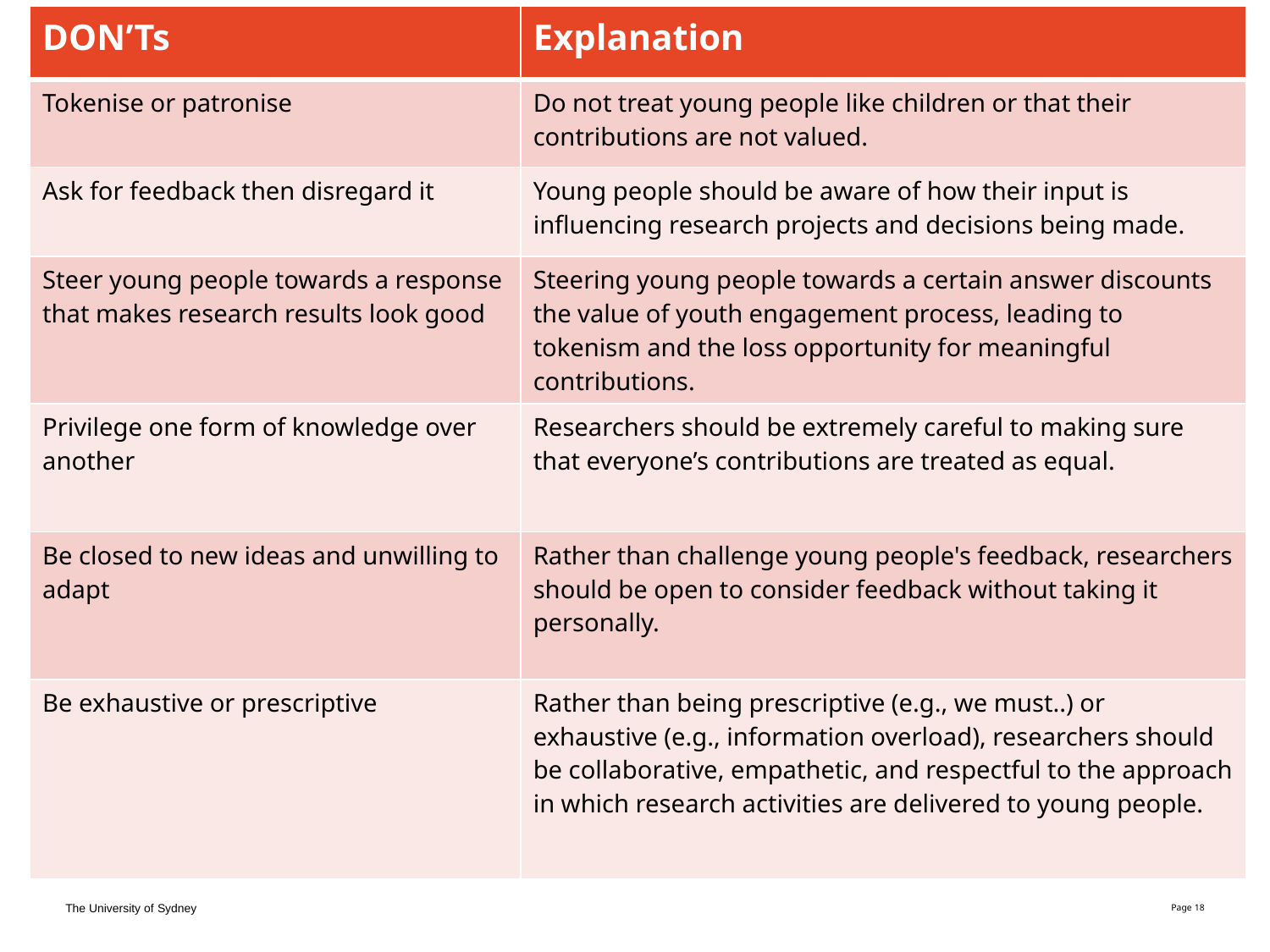

| DON’Ts | Explanation |
| --- | --- |
| Tokenise or patronise | Do not treat young people like children or that their contributions are not valued. |
| Ask for feedback then disregard it | Young people should be aware of how their input is influencing research projects and decisions being made. |
| Steer young people towards a response that makes research results look good | Steering young people towards a certain answer discounts the value of youth engagement process, leading to tokenism and the loss opportunity for meaningful contributions. |
| Privilege one form of knowledge over another | Researchers should be extremely careful to making sure that everyone’s contributions are treated as equal. |
| Be closed to new ideas and unwilling to adapt | Rather than challenge young people's feedback, researchers should be open to consider feedback without taking it personally. |
| Be exhaustive or prescriptive | Rather than being prescriptive (e.g., we must..) or exhaustive (e.g., information overload), researchers should be collaborative, empathetic, and respectful to the approach in which research activities are delivered to young people. |
# BREAKING IT DOWN – DON’Ts

## Slide 19
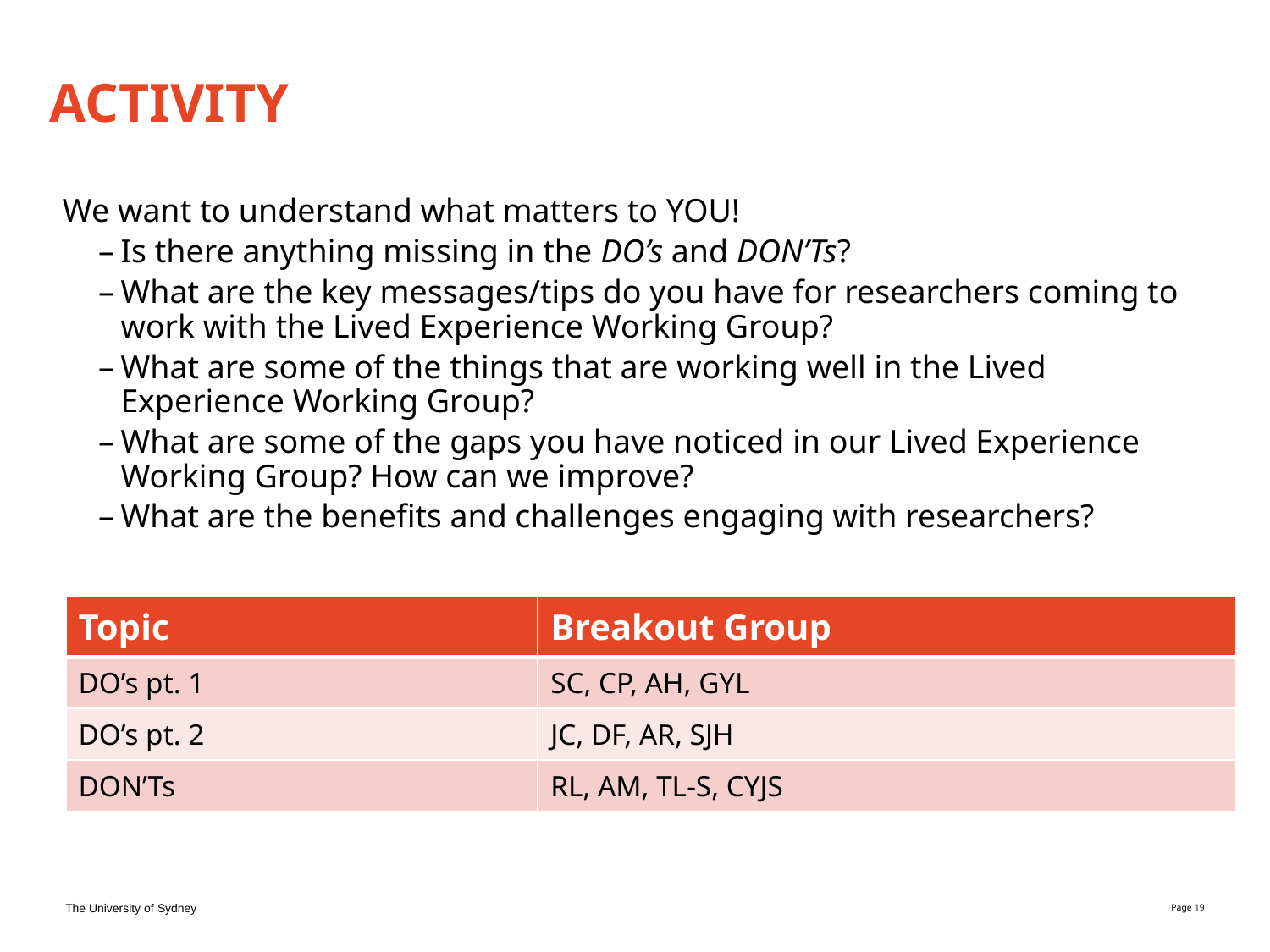

# ACTIVITY
We want to understand what matters to YOU!
Is there anything missing in the DO’s and DON’Ts?
What are the key messages/tips do you have for researchers coming to work with the Lived Experience Working Group?
What are some of the things that are working well in the Lived Experience Working Group?
What are some of the gaps you have noticed in our Lived Experience Working Group? How can we improve?
What are the benefits and challenges engaging with researchers?
| Topic | Breakout Group |
| --- | --- |
| DO’s pt. 1 | SC, CP, AH, GYL |
| DO’s pt. 2 | JC, DF, AR, SJH |
| DON’Ts | RL, AM, TL-S, CYJS |

## Slide 20
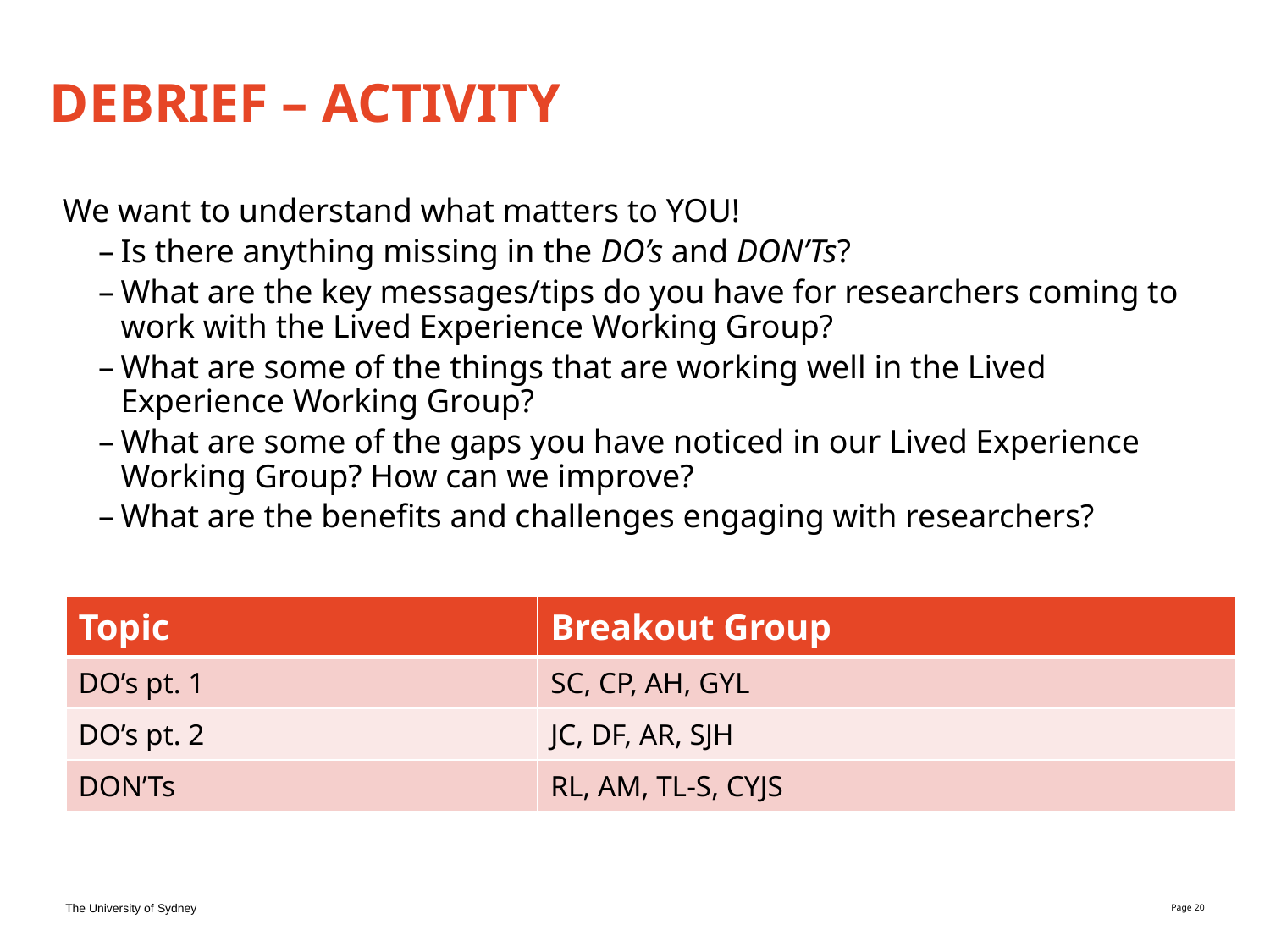

# DEBRIEF – ACTIVITY
We want to understand what matters to YOU!
Is there anything missing in the DO’s and DON’Ts?
What are the key messages/tips do you have for researchers coming to work with the Lived Experience Working Group?
What are some of the things that are working well in the Lived Experience Working Group?
What are some of the gaps you have noticed in our Lived Experience Working Group? How can we improve?
What are the benefits and challenges engaging with researchers?
| Topic | Breakout Group |
| --- | --- |
| DO’s pt. 1 | SC, CP, AH, GYL |
| DO’s pt. 2 | JC, DF, AR, SJH |
| DON’Ts | RL, AM, TL-S, CYJS |

## Slide 21
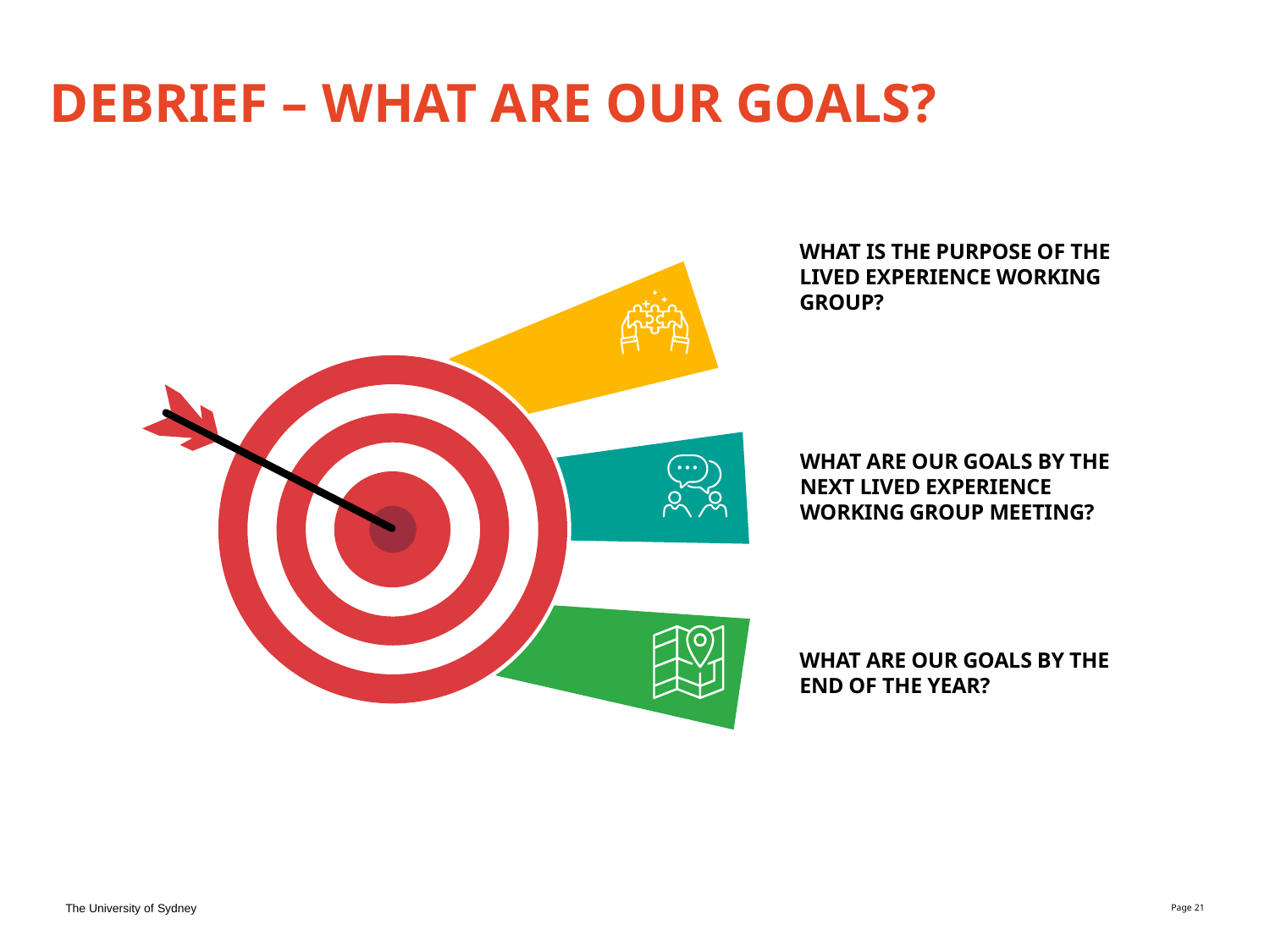

# DEBRIEF – WHAT ARE OUR GOALS?
WHAT IS THE PURPOSE OF THE LIVED EXPERIENCE WORKING GROUP?
WHAT ARE OUR GOALS BY THE NEXT LIVED EXPERIENCE WORKING GROUP MEETING?
WHAT ARE OUR GOALS BY THE END OF THE YEAR?

## Slide 22
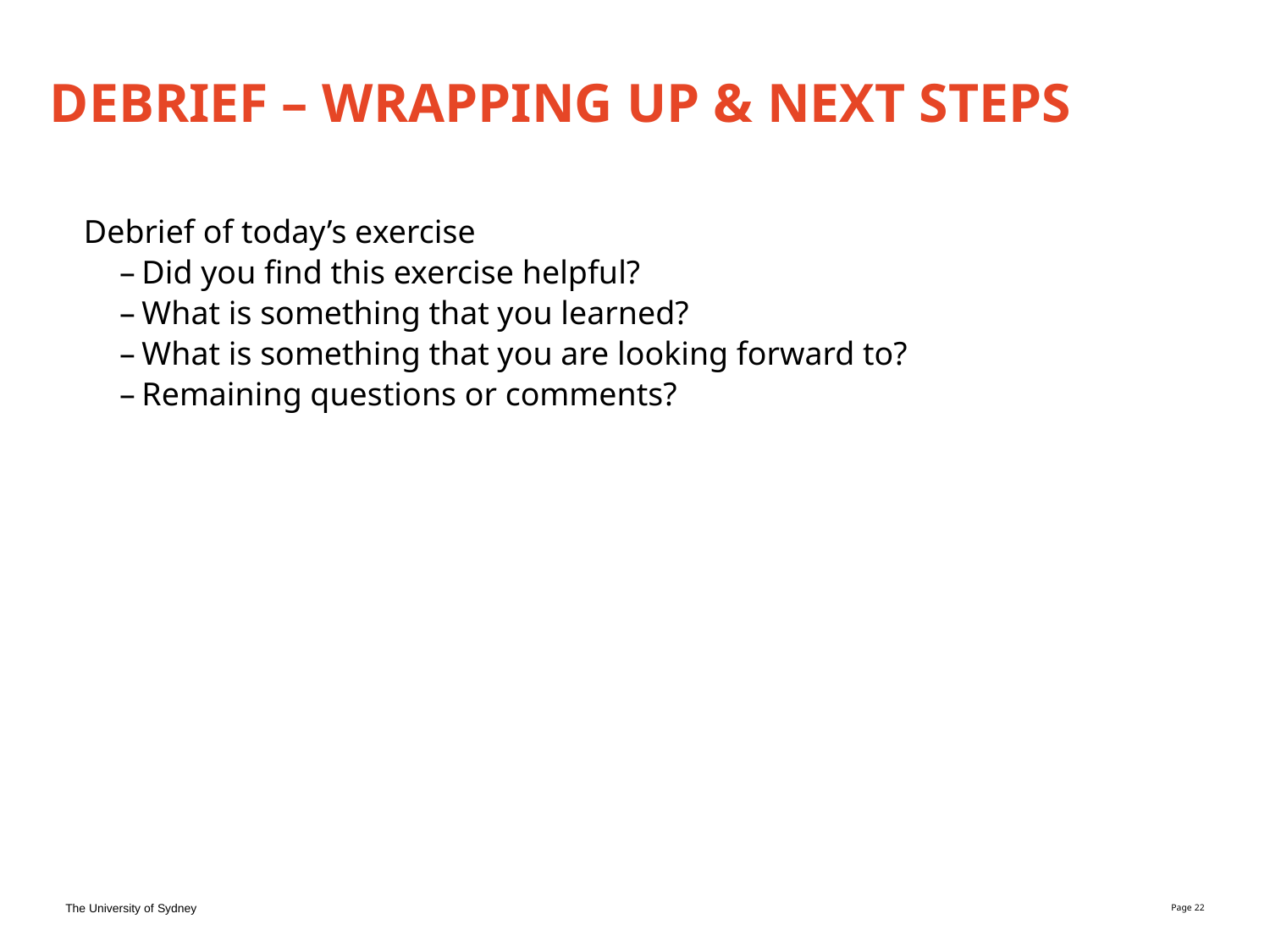

# DEBRIEF – WRAPPING UP & NEXT STEPS
Debrief of today’s exercise
Did you find this exercise helpful?
What is something that you learned?
What is something that you are looking forward to?
Remaining questions or comments?

## Slide 23
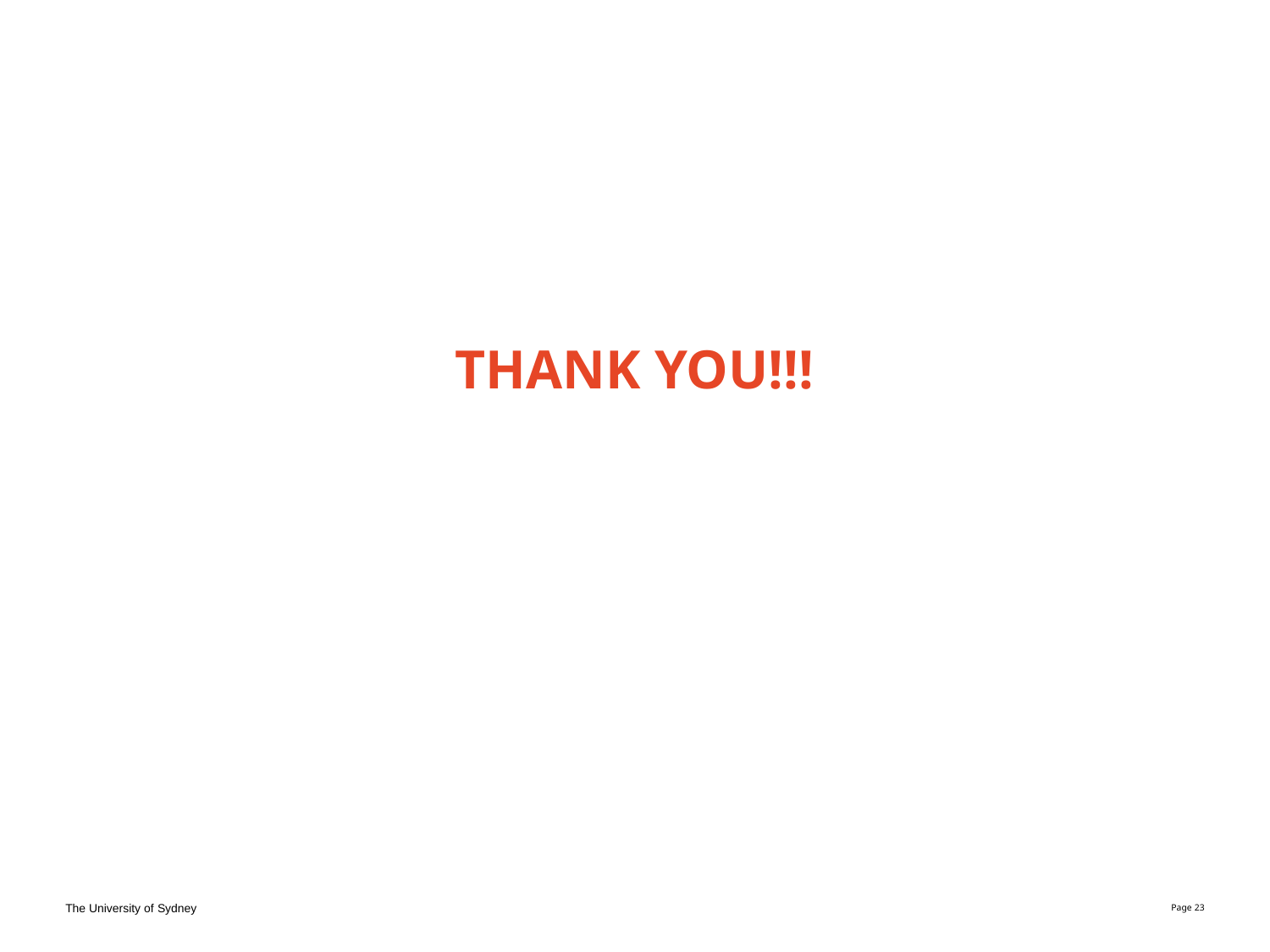

# THANK YOU!!!
