# Supplementary material for: Strengthening mental health research outcomes through genuine partnerships with young people with lived or living experience: A pilot evaluation study
Source: Health Expect. 2023 May 17;26(4):1703–15. doi: 10.1111/hex.13777 (PMC10349217; doi:10.1111/hex.13777)
Supplement: Supplementary file 6 — Supporting Information. [file HEX-26--s002.docx]

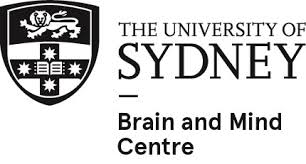


**YOUTH MENTAL HEALTH & TECHNOLOGY TEAM:**

**LIVED EXPERIENCE WORKING GROUP**

**Terms of Reference**

January 2022

***Acknowledgement of Country.*** *We would like to show our respect and acknowledge the Traditional Custodians of the Land, of Elders past, present and future, on which this meeting takes place.*

***Acknowledgement of Lived Experience.*** *We acknowledge and value those touched directly or indirectly by mental ill health and suicidality, who have experienced the health system and contribute their valuable inputs so that we may learn and grow to provide services and products that meet their needs.*

**Purpose**

The Lived Experience Working Group will embed lived experience input on the design, development, implementation and evaluation on current and future research protocols specific to the Youth Mental Health & Technology team at the University of Sydney’s Brain and Mind Centre.

There are currently six broad streams of research within the Youth Mental Health & Technology team:

1. Systems modelling, simulation and data science
2. Digital technology
3. Brain and Mind Centre Youth Model
4. Circadian models
5. Immune-metabolic pathways
6. Mental wealth

## **Functions**

The Lived Experience Working Group will:

1. Provide lived experience expertise.
2. Be a key link between and across Youth Mental Health & Technology team research and extended youth mental health lived experience communities; and
3. Provide strategic and operational advice to ensure all Youth Mental Health & Technology team’s research streams will deliver relevant and informed content.

Topics of discussion will be guided by the needs of the Youth Mental Health & Technology team that require further input, as flagged by the Working Group and/or relevant researchers.

## **Working Group Membership**

The Working Group has representation from young people (aged 16 – 30 years old) with a lived mental health experience. Membership also covers diverse representation of gender, sexuality, culture (Culturally And Linguistically Diverse, Aboriginal and Torres Strait Islander), education, work, and mental health experience.

**Meetings**

The Working Group will meet of a Wednesday evening on a monthly basis and will be guided by two facilitators – one of whom will be a young person with a lived mental health experience, and one whom will be a mental health professional whose role will be to respond to any concerns of distress as a result of the subject matter. Ad-hoc meetings may be requested if required, subject to follow-up actions identified during Working Group meetings.

Meetings will be held at The University of Sydney’s Brain and Mind Centre, with the option to participate through videoconferencing.

**Communication**

The Working Group will utilise email and/or *Slack*. No research data will be collected via these channels, but rather, will be used as a communication tool between scheduled Working Group meetings. For instance, the Working Group may utilise *Slack* to provide further feedback and ask questions. On the other hand, the research team who is responsible for the administrative planning of the Working Group – henceforth referred as the ‘Planning Group’ – may utilise *Slack* to provide information on upcoming events (such as interesting webinars), circulate online surveys, provide pre-briefs, etc. Pre-briefs are referred to as information/pre-information provided prior to the actual Working Group meeting. At a minimum, pre-briefs will include:

- An agenda for the upcoming meeting
- An introduction of the research topic which includes disclaimers of potentially sensitive topics
- *(When relevant):* An update from the Youth Mental Health & Technology team researcher(s) on how the Working Group’s contributions have impacted their research
- Information on reimbursement (see below)

In addition to *Slack* and email, pre-briefs will be provided on the day of the Working Group meeting. Specifically, the first 30 minutes of each meeting will be dedicated to reviewing the meeting material to allow the Working Group to ask questions and provide feedback in a safe and familiar setting.

When possible, responses from the Working Group and Planning Group is requested within three business days.

**Recruitment**

Recruitment will be facilitated by the Planning Group, with input from the Working Group members when possible. To maintain cohesiveness and build ongoing trust, Working Group membership is capped to ten young people.

Working Group members can nominate new members to ensure diversity within the group; however, this will be facilitated by the Planning Group with input from the rest of the Working Group.

When necessary (e.g., Youth Mental Health & Technology researcher would like to conduct a co-design workshop with the Working Group), a second tier of lived experience participation may be formed whereby members of the Working Group can invite their extended networks to contribute.

**Confidentiality and Privacy**

Members may have contact with confidential or personal information. If so, members are required to maintain the security of any confidential or personal information and not access, use or remove any information, unless the member is authorised to do so. Members who disclose trade secrets or confidential business information – such as unpublished data – will be asked to resign from the group.

Likewise, all researchers who engage with the Working Group will be required to consent to confidentiality, recognising that Working Group members may share personal experiences regarding their mental health.

**Research Policy**

All the research streams of the Youth Mental Health & Technology team, including those which the Lived Experience Working Group provide recommendations to, follow the University of Sydney’s Research Code of Conduct 2019. To read follow this [link](https://www.sydney.edu.au/policies/showdoc.aspx?recnum=PDOC2013/321&RendNum=0).

**Indigenous Strategy**

In alignment with the University of Sydney’s Indigenous strategy which outlines our commitment to creating higher education and leadership opportunities for Aboriginal and Torres Strait Islander peoples and valuing Indigenous culture (to read follow this [link](https://www.sydney.edu.au/about-us/vision-and-values/our-aboriginal-and-torres-strait-islander-community.html)). We will endeavour to ensure research outcomes are culturally competent and are in alignment with the National Health and Medical Research Council, *Ethical conduct in research with Aboriginal and Torres Strait Islander Peoples and communities: Guidelines for researchers and stakeholders* (2018), Commonwealth of Australia: Canberra.

**Disability Inclusion strategy**

The Lived Experience Working Group will comply with the University of Sydney’s Disability Inclusion action plan, which outlines the commitment to ensure inclusion of individuals with any form of disability. This encourages a culture whereby all participating members of the Working Group can be challenged to achieve their full potential and capabilities (to read follow this [link](https://www.sydney.edu.au/content/dam/corporate/documents/about-us/values-and-visions/disability-inclusion-action-plan-2019-24/disability-inclusion-action-plan-2019-24.pdf)).

**Advocacy**

The Lived Experience Working Group will work in partnership with researchers of the Brain and Mind Centre’s Youth Mental Health & Technology team to advocate for a better mental health care for all young Australians. Advocacy may be demonstrated through various activities, such as but not limited to, webinars, podcasts and policy reports.

**Quorum**

Meetings will only proceed if 50% +1 of the Working Group are available to attend.

**Participation Reimbursement**

To formally recognise the valuable, special contributions made, The Youth Mental Health & Technology team at the University of Sydney’s Brain and Mind Centre will reimburse members who have a lived experience of mental ill health, or who are a family member or support person of someone with mental ill health. Reimbursement will be made when individuals:

- are invited by the Youth Mental Health & Technology team to make a significant contribution to its work;
- are not funded by another organisation, group or council to represent it in engagements with the Youth Mental Health & Technology team; and/or
- express that they would like to volunteer (i.e., chooses not to be reimbursed).

**Participants will be reimbursed in the form of an online voucher of their choice (Woolworths, Big W, Caltex, Coles, Target, Kmart, Prepaid Mobile Recharge, or Monetary Card) to** **ensure that payment does not interfere with an individual’s ability to access government aid.**

Members are encouraged to include their participation in the Lived Experience Working Group research project on their Curriculum Vitae (or Resume). At a minimum, we suggest that the following information is included:

***Role:*** Research Consultant
***Group:*** Youth Lived Experience Working Group
***Organisation:*** Brain and Mind Centre, The University of Sydney

Members must provide information on their online voucher of choice ***on the day of the Working Group meeting*** (e.g., during the break). If there is no communication, the Working Group member will receive a voucher from the same retail organisation as the previous meeting.

**Reimbursement Rates**

Individuals who are eligible for participant reimbursement will be recognised for their contribution on a daily rate or an hourly basis where less than eight hours is required. This will be based on the following payment rates:

| **Activity** | **Daily rate (8 hours or more)** | **Hourly rate** |
| --- | --- | --- |
| Lived experience research contributions | $418.00 | $52.25 |

Unless agreed in advance with the Youth Mental Health & Technology team, reimbursement for preparation time is not included and should not be included in the number of hours submitted for payment.

The maximum time for working that may be reimbursed in one day is 8 hours. Any travel time spent by a participant to attend an engagement by the Youth Mental Health & Technology team will be a time cost to the member and not paid by the Brain and Mind Centre.
