# Supplementary material for: Strengthening mental health research outcomes through genuine partnerships with young people with lived or living experience: A pilot evaluation study
Source: Health Expect. 2023 May 17;26(4):1703–15. doi: 10.1111/hex.13777 (PMC10349217; doi:10.1111/hex.13777)
Supplement: Supplementary file 7 — Supporting Information. [file HEX-26--s006.pptx]

## Slide 1
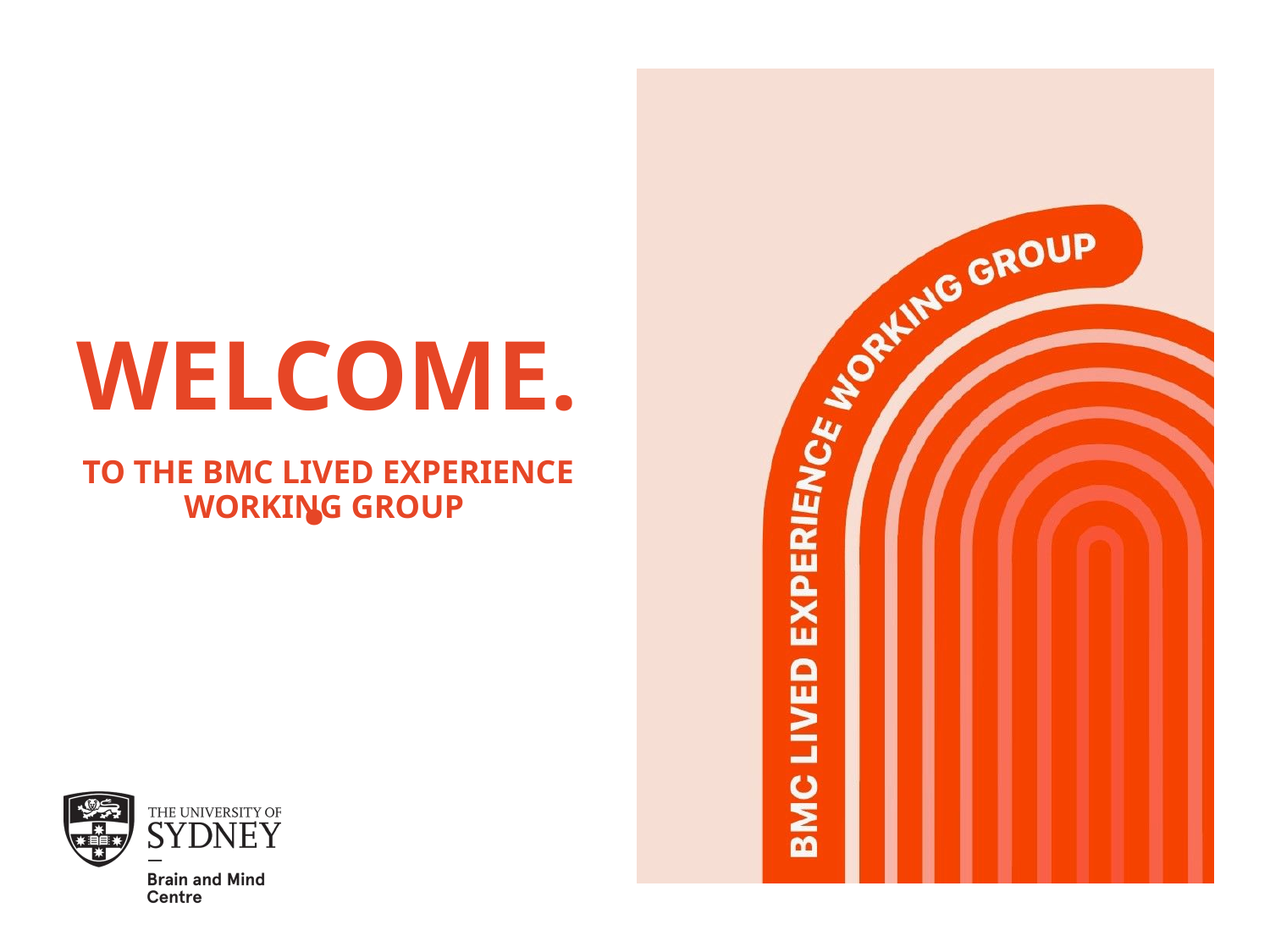

# WELCOME..
TO THE BMC LIVED EXPERIENCE WORKING GROUP

## Slide 2
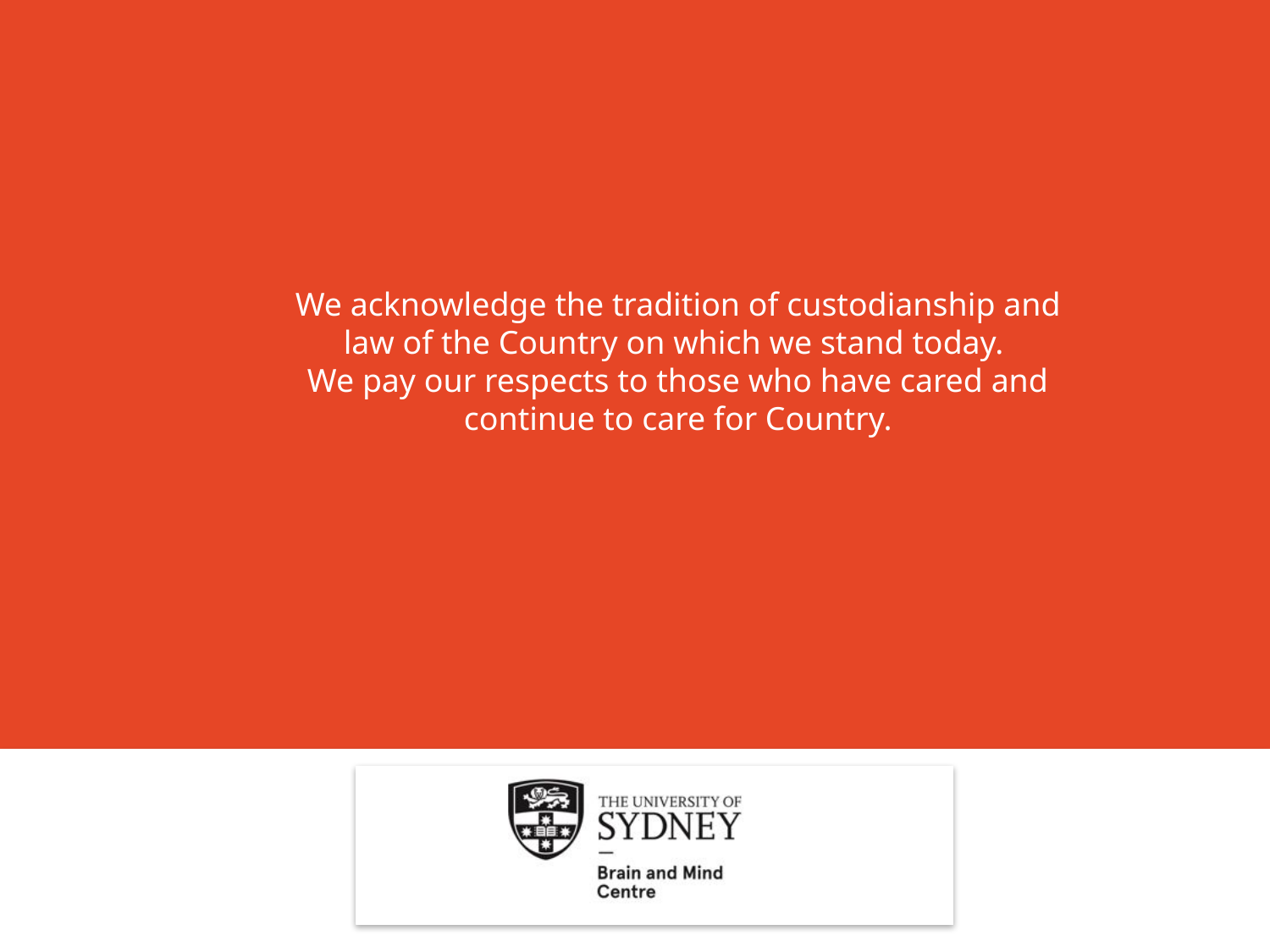

## Slide 3
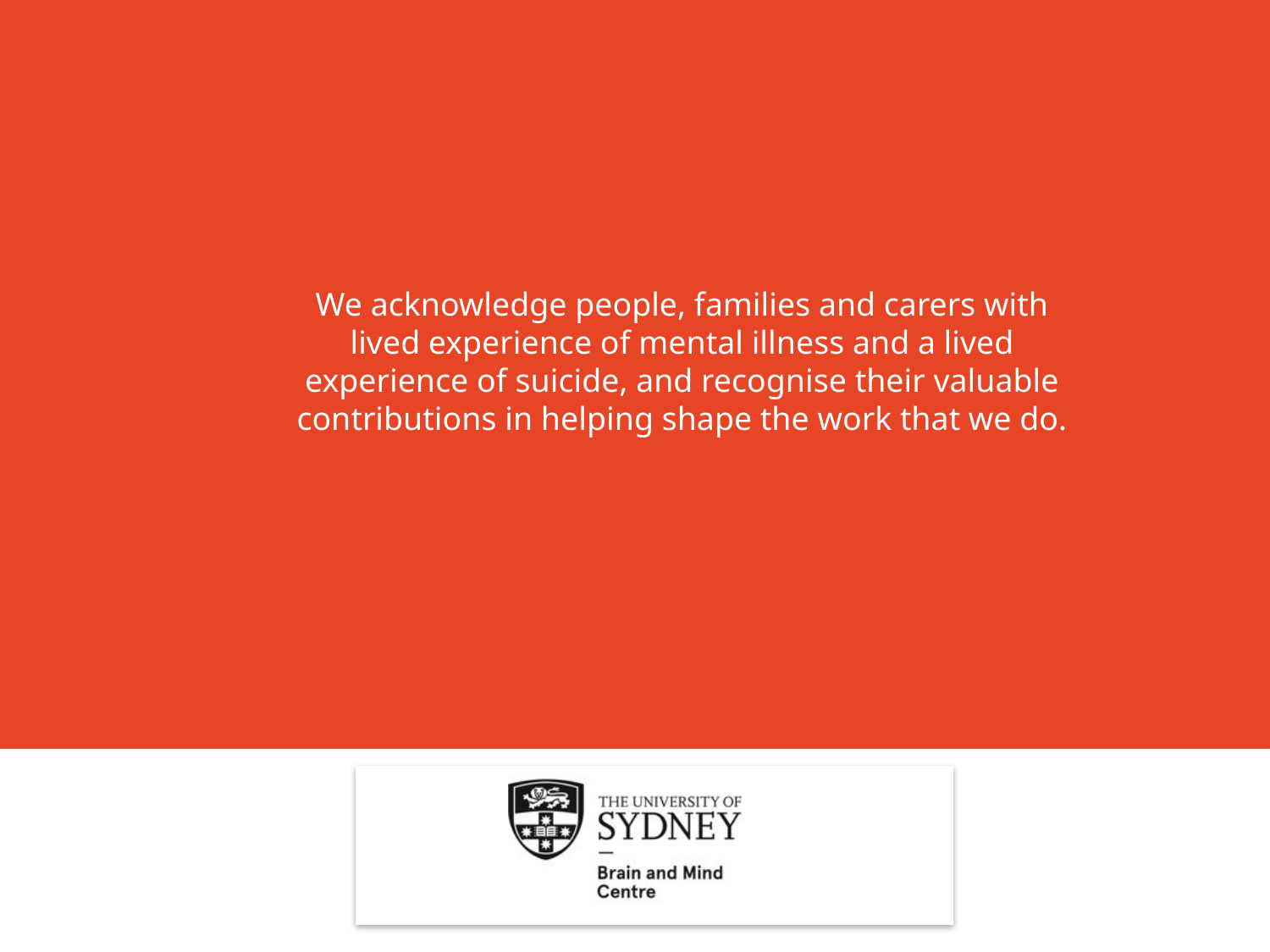

## Slide 4
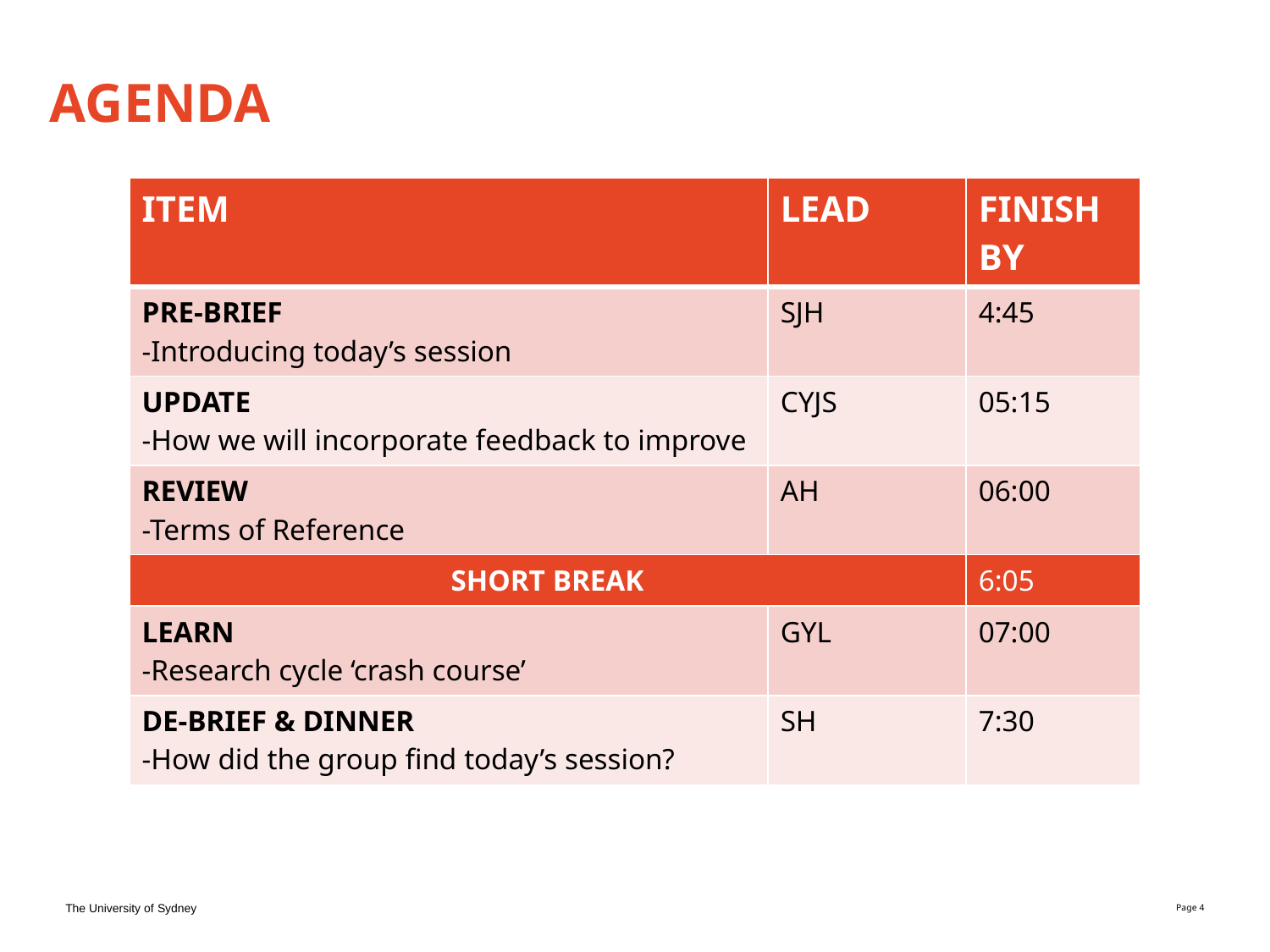

# AGENDA
| ITEM | LEAD | FINISH BY |
| --- | --- | --- |
| PRE-BRIEF -Introducing today’s session | SJH | 4:45 |
| UPDATE -How we will incorporate feedback to improve | CYJS | 05:15 |
| REVIEW -Terms of Reference | AH | 06:00 |
| SHORT BREAK | | 6:05 |
| LEARN -Research cycle ‘crash course’ | GYL | 07:00 |
| DE-BRIEF & DINNER -How did the group find today’s session? | SH | 7:30 |

## Slide 5
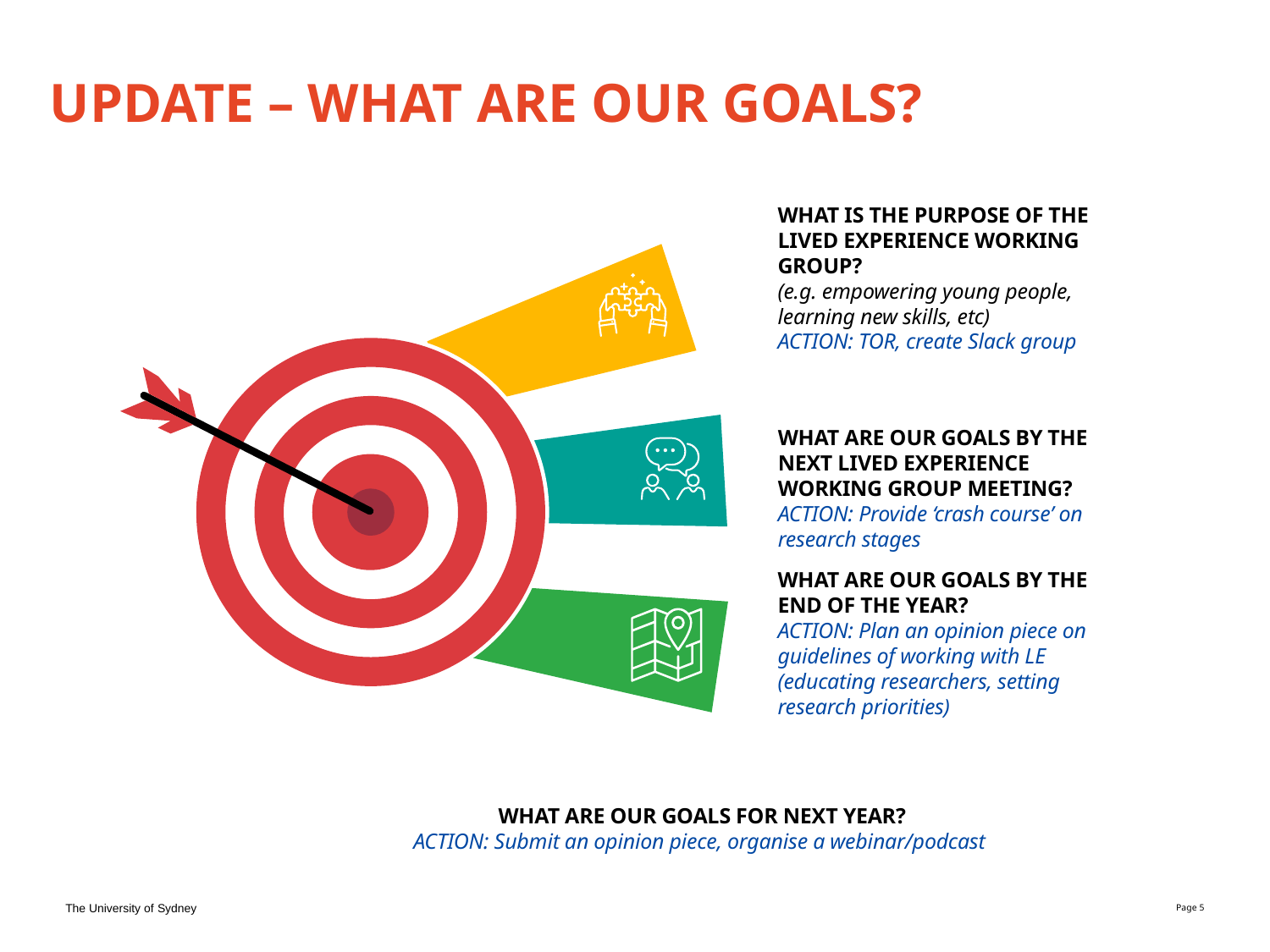

# UPDATE – WHAT ARE OUR GOALS?
WHAT IS THE PURPOSE OF THE LIVED EXPERIENCE WORKING GROUP?
(e.g. empowering young people, learning new skills, etc)
ACTION: TOR, create Slack group
WHAT ARE OUR GOALS BY THE NEXT LIVED EXPERIENCE WORKING GROUP MEETING?
ACTION: Provide ‘crash course’ on research stages
WHAT ARE OUR GOALS BY THE END OF THE YEAR?
ACTION: Plan an opinion piece on guidelines of working with LE (educating researchers, setting research priorities)
WHAT ARE OUR GOALS FOR NEXT YEAR?
ACTION: Submit an opinion piece, organise a webinar/podcast

## Slide 6
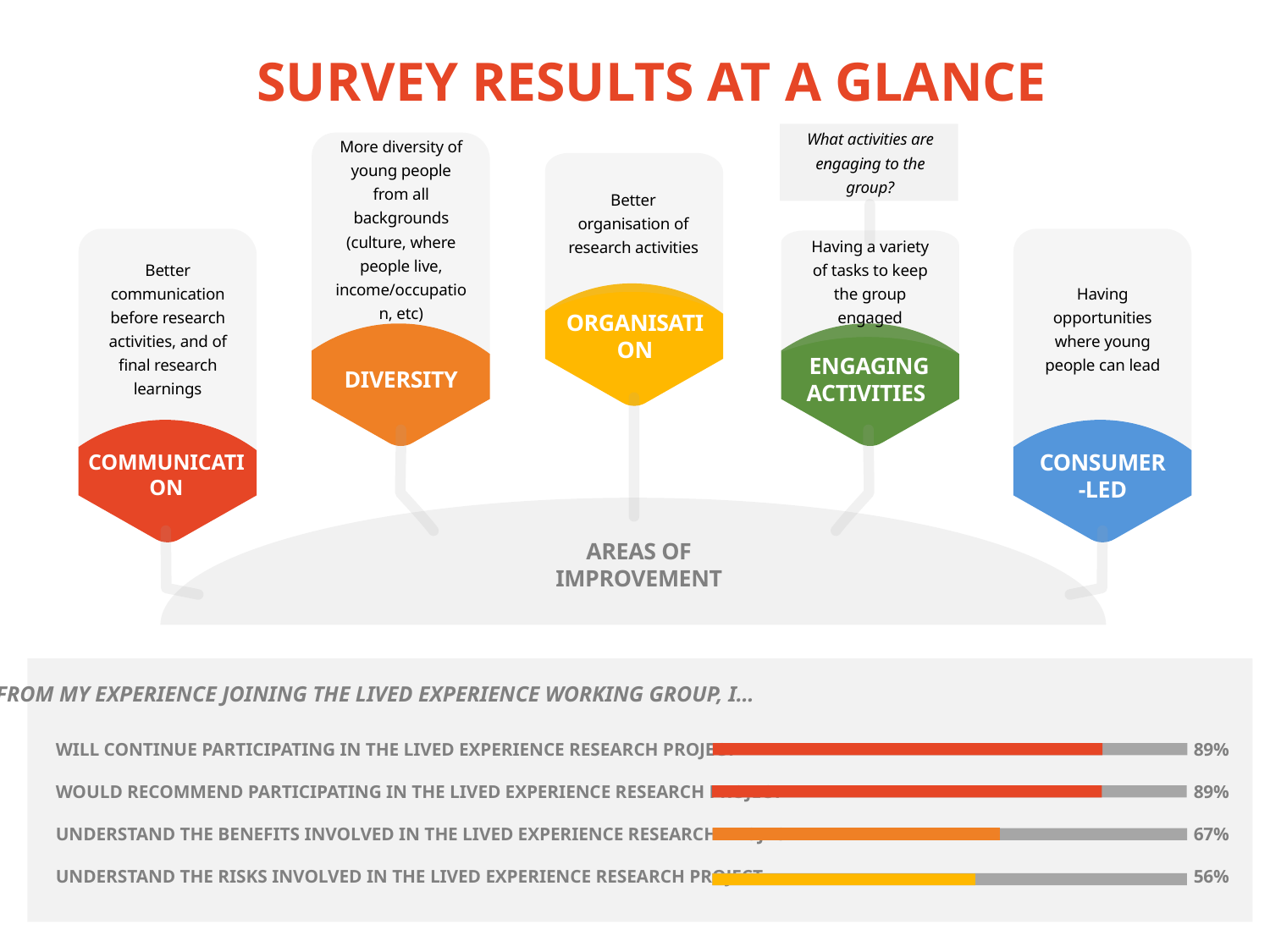

SURVEY RESULTS AT A GLANCE
What activities are engaging to the group?
More diversity of young people from all backgrounds (culture, where people live, income/occupation, etc)
Better organisation of research activities
Having a variety of tasks to keep the group engaged
Better communication before research activities, and of final research learnings
Having opportunities where young people can lead
ORGANISATION
ENGAGING ACTIVITIES
DIVERSITY
CONSUMER-LED
COMMUNICATION
AREAS OF IMPROVEMENT
FROM MY EXPERIENCE JOINING THE LIVED EXPERIENCE WORKING GROUP, I…
WILL CONTINUE PARTICIPATING IN THE LIVED EXPERIENCE RESEARCH PROJECT
WOULD RECOMMEND PARTICIPATING IN THE LIVED EXPERIENCE RESEARCH PROJECT
UNDERSTAND THE BENEFITS INVOLVED IN THE LIVED EXPERIENCE RESEARCH PROJECT
UNDERSTAND THE RISKS INVOLVED IN THE LIVED EXPERIENCE RESEARCH PROJECT
89%
89%
67%
56%

## Slide 7
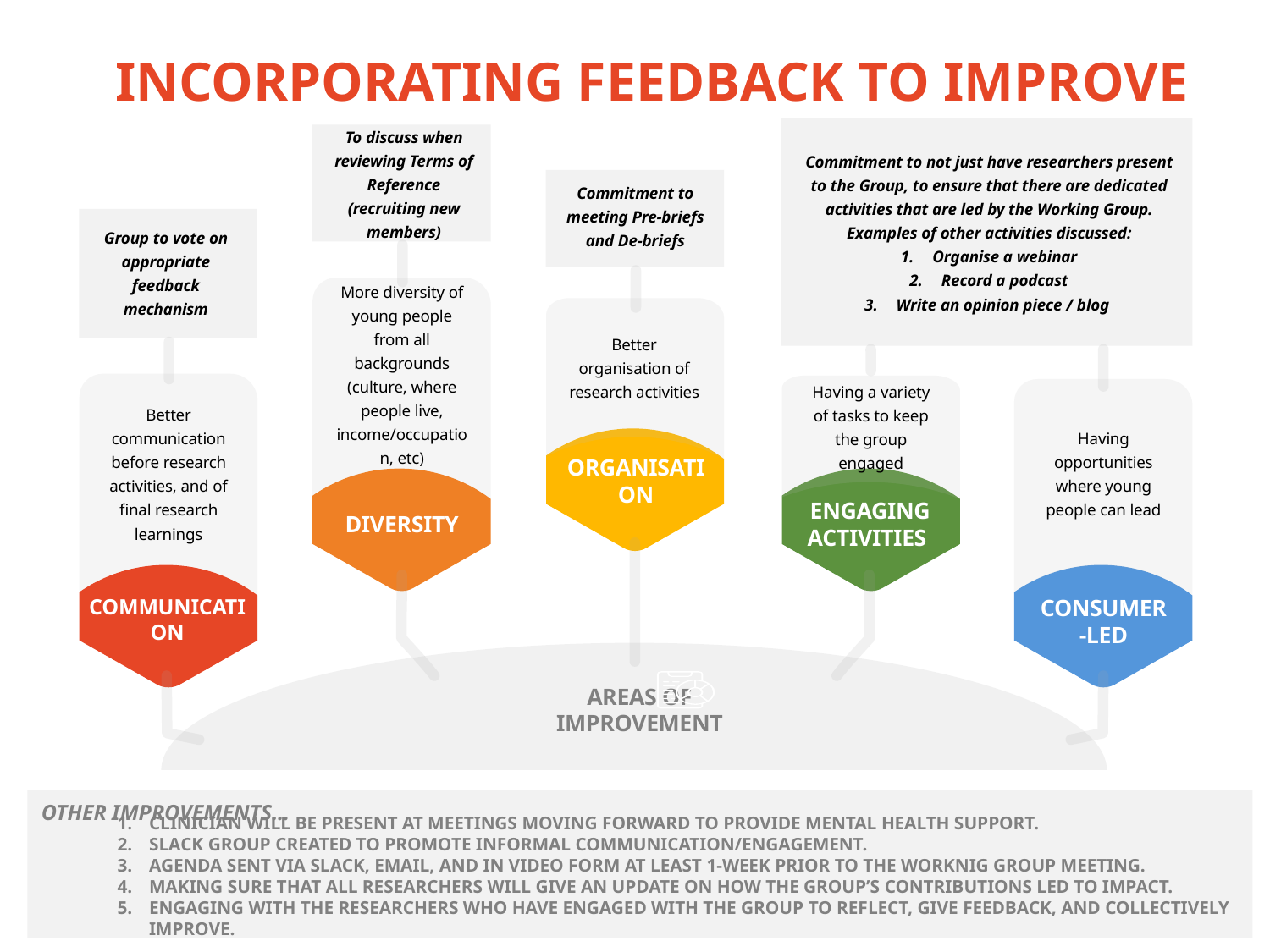

INCORPORATING FEEDBACK TO IMPROVE
To discuss when reviewing Terms of Reference (recruiting new members)
Commitment to not just have researchers present to the Group, to ensure that there are dedicated activities that are led by the Working Group.
Examples of other activities discussed:
Organise a webinar
Record a podcast
Write an opinion piece / blog
Commitment to meeting Pre-briefs and De-briefs
Group to vote on appropriate feedback mechanism
More diversity of young people from all backgrounds (culture, where people live, income/occupation, etc)
Better organisation of research activities
Having a variety of tasks to keep the group engaged
Better communication before research activities, and of final research learnings
Having opportunities where young people can lead
ORGANISATION
ENGAGING ACTIVITIES
DIVERSITY
CONSUMER-LED
COMMUNICATION
AREAS OF IMPROVEMENT
OTHER IMPROVEMENTS…
CLINICIAN WILL BE PRESENT AT MEETINGS MOVING FORWARD TO PROVIDE MENTAL HEALTH SUPPORT.
SLACK GROUP CREATED TO PROMOTE INFORMAL COMMUNICATION/ENGAGEMENT.
AGENDA SENT VIA SLACK, EMAIL, AND IN VIDEO FORM AT LEAST 1-WEEK PRIOR TO THE WORKNIG GROUP MEETING.
MAKING SURE THAT ALL RESEARCHERS WILL GIVE AN UPDATE ON HOW THE GROUP’S CONTRIBUTIONS LED TO IMPACT.
ENGAGING WITH THE RESEARCHERS WHO HAVE ENGAGED WITH THE GROUP TO REFLECT, GIVE FEEDBACK, AND COLLECTIVELY IMPROVE.

## Slide 8
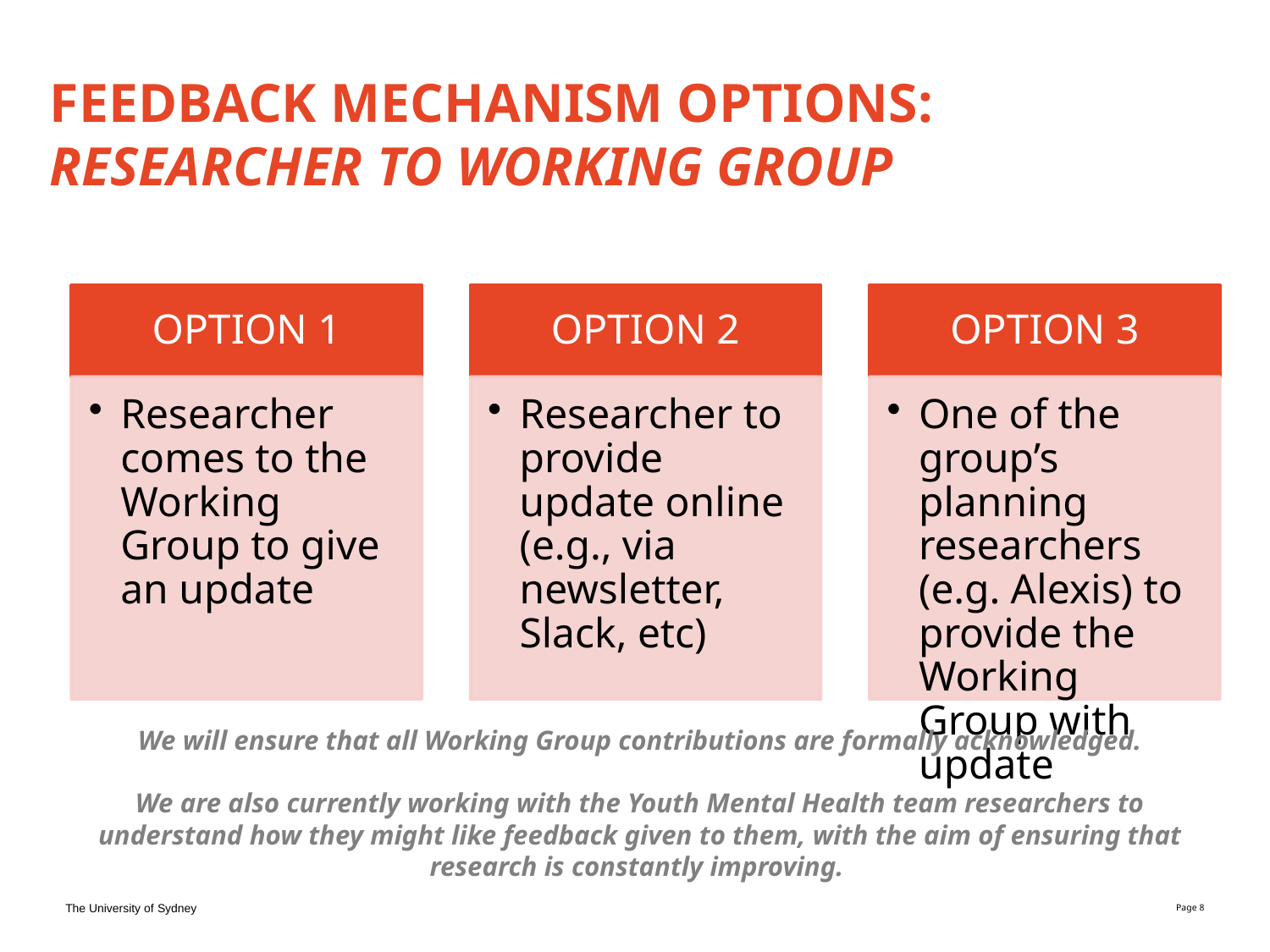

# FEEDBACK MECHANISM OPTIONS: RESEARCHER TO WORKING GROUP
We will ensure that all Working Group contributions are formally acknowledged.
We are also currently working with the Youth Mental Health team researchers to understand how they might like feedback given to them, with the aim of ensuring that research is constantly improving.

## Slide 9
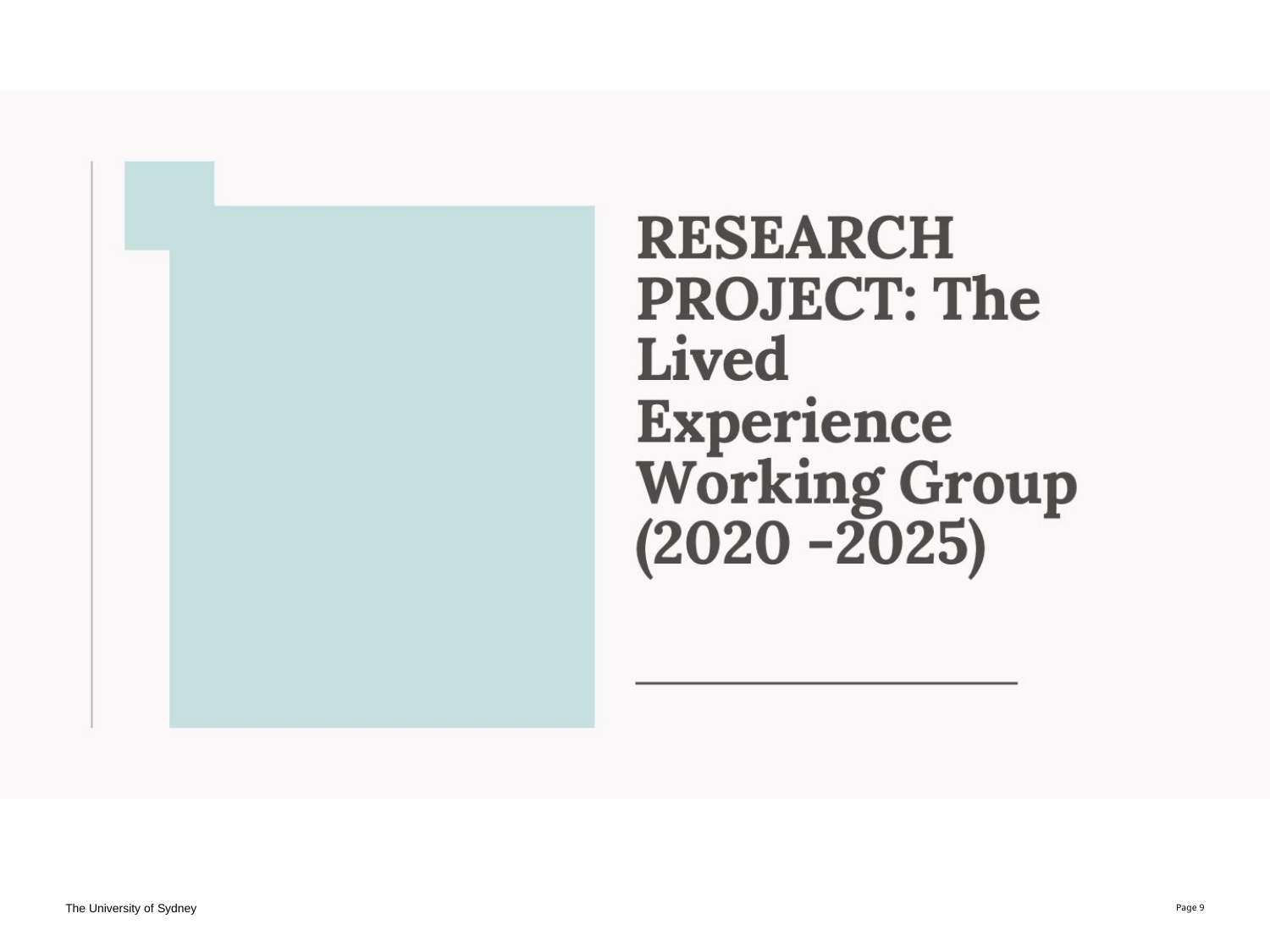

## Slide 10
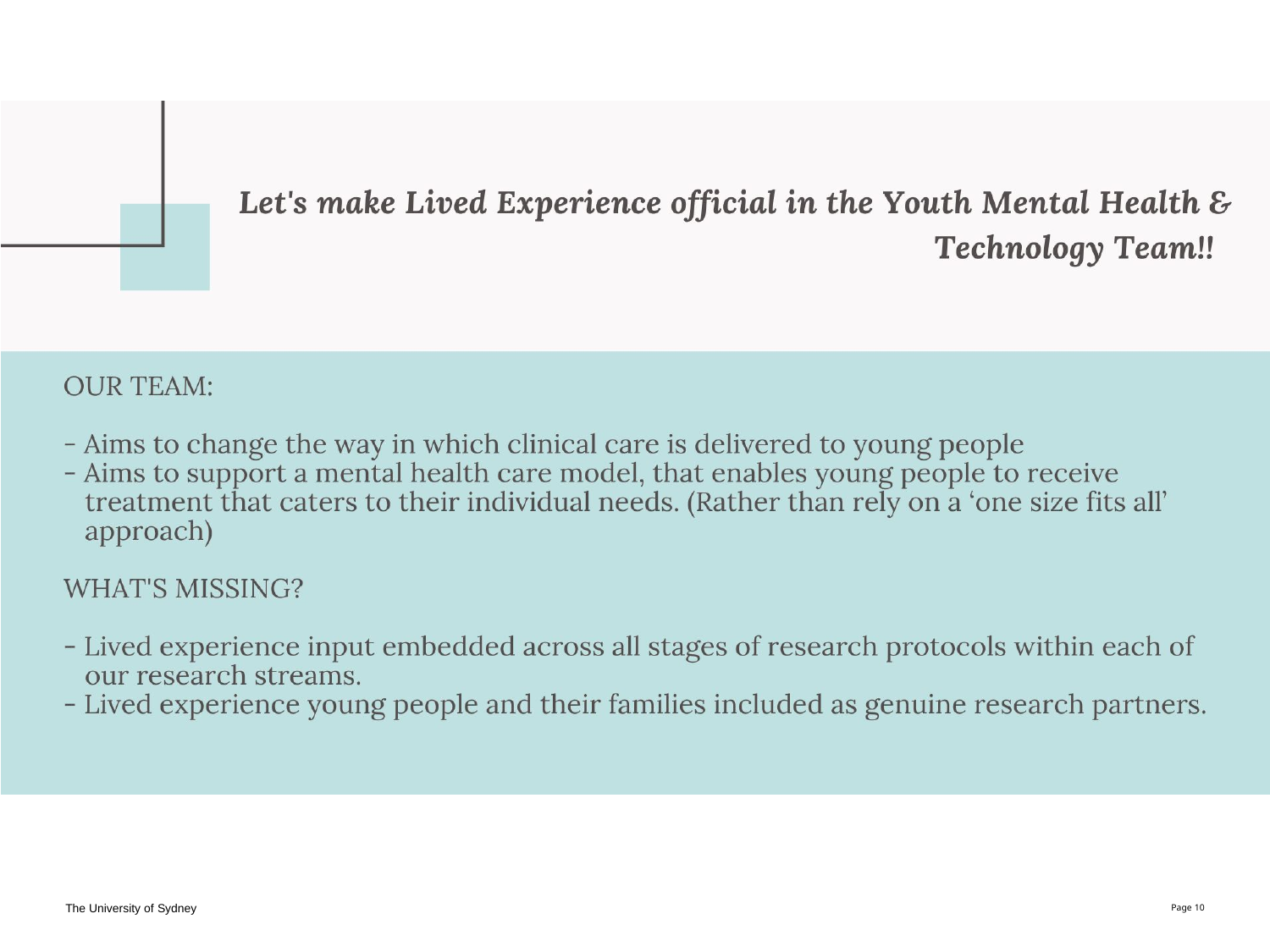

## Slide 11
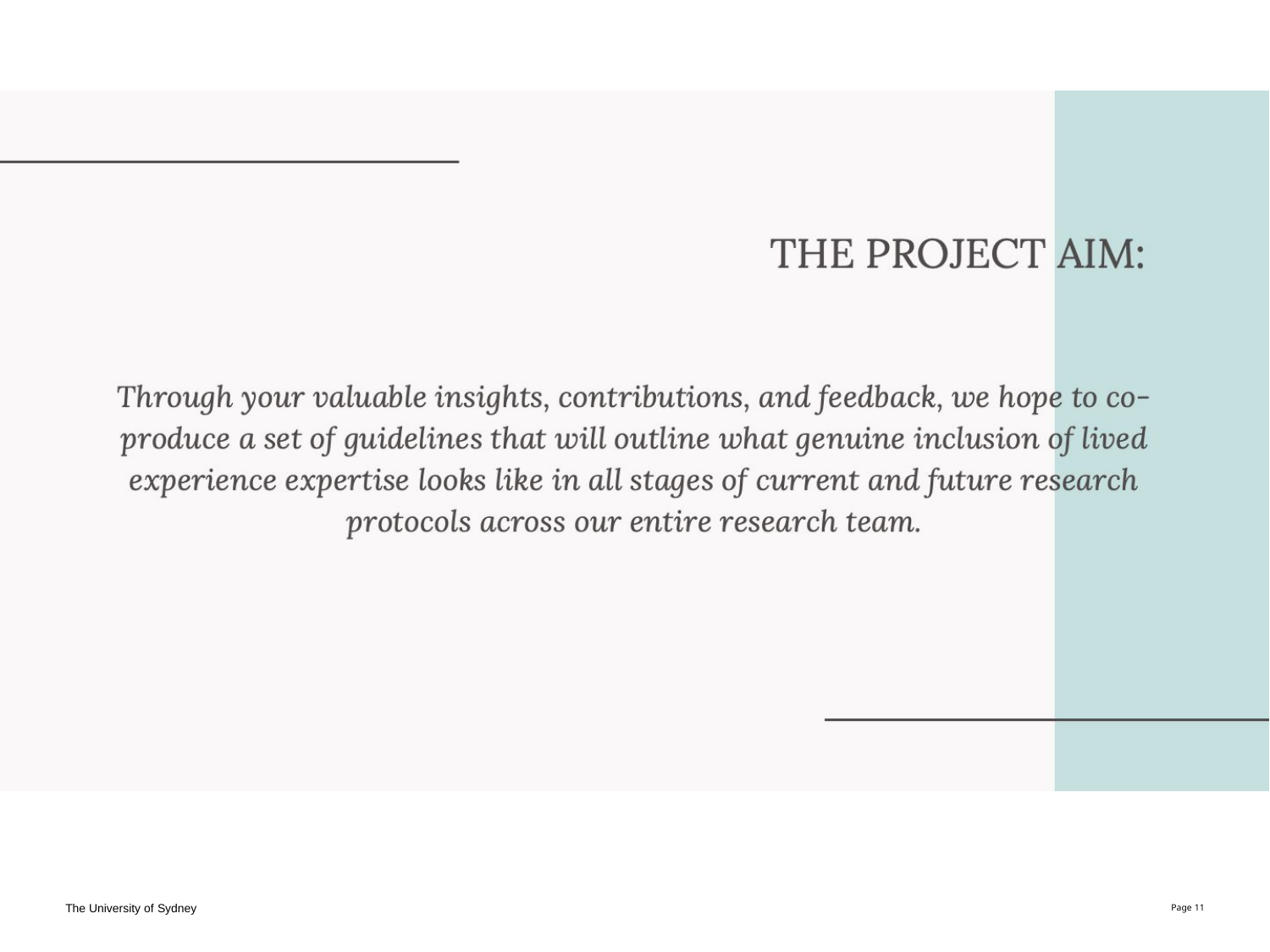

## Slide 12
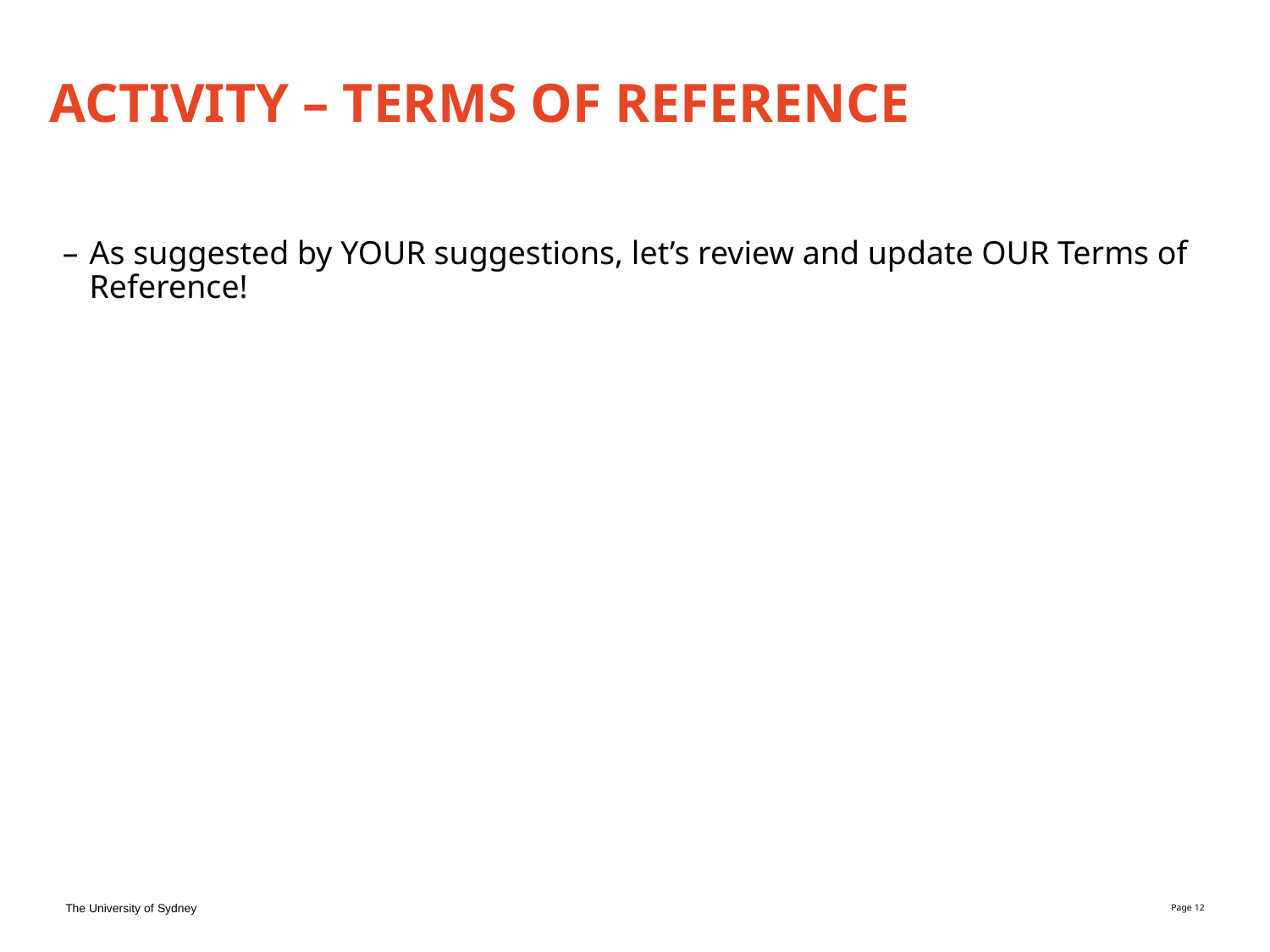

# ACTIVITY – TERMS OF REFERENCE
As suggested by YOUR suggestions, let’s review and update OUR Terms of Reference!

## Slide 13
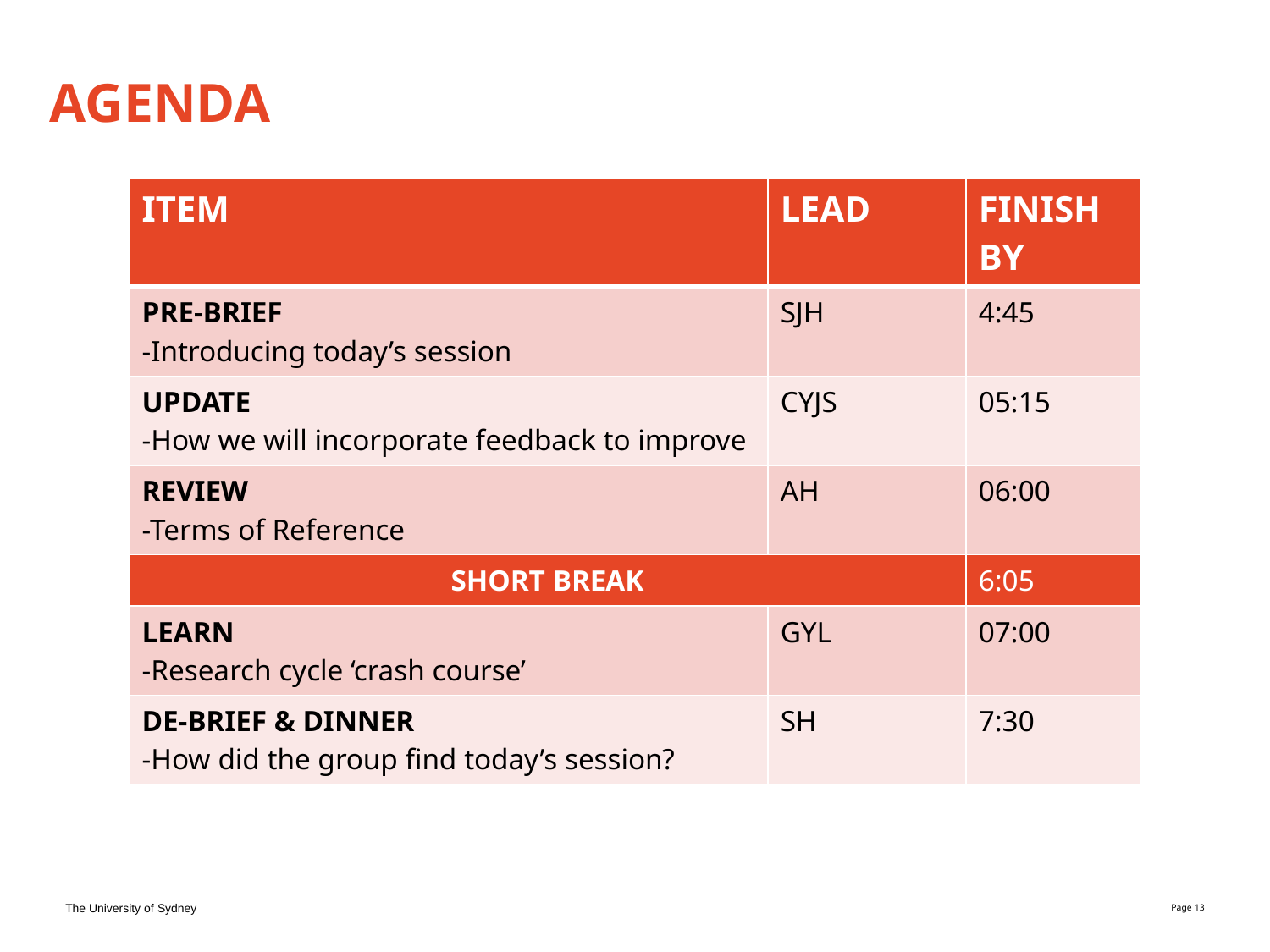

# AGENDA
| ITEM | LEAD | FINISH BY |
| --- | --- | --- |
| PRE-BRIEF -Introducing today’s session | SJH | 4:45 |
| UPDATE -How we will incorporate feedback to improve | CYJS | 05:15 |
| REVIEW -Terms of Reference | AH | 06:00 |
| SHORT BREAK | | 6:05 |
| LEARN -Research cycle ‘crash course’ | GYL | 07:00 |
| DE-BRIEF & DINNER -How did the group find today’s session? | SH | 7:30 |

## Slide 14
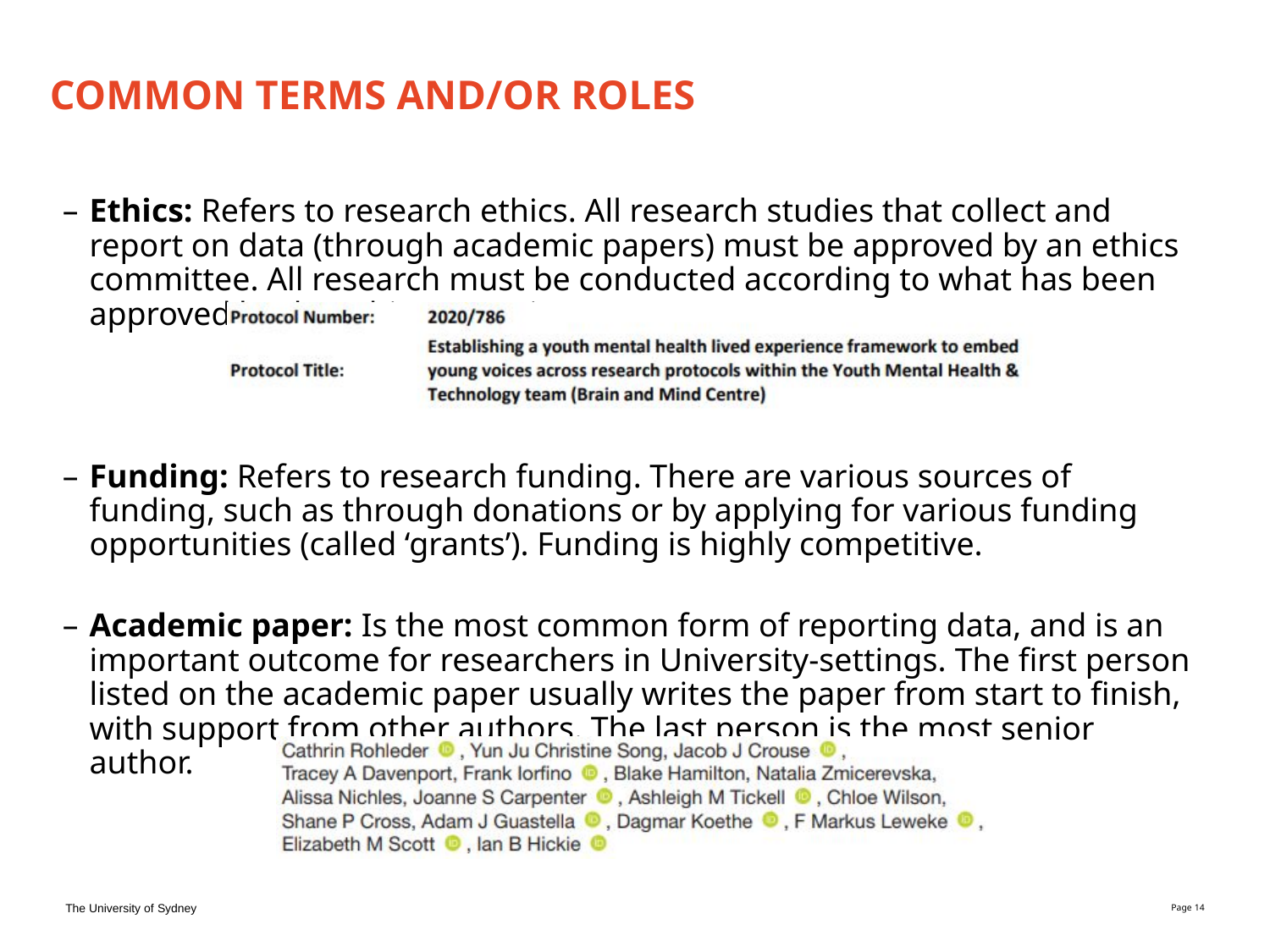

# COMMON TERMS AND/OR ROLES
Ethics: Refers to research ethics. All research studies that collect and report on data (through academic papers) must be approved by an ethics committee. All research must be conducted according to what has been approved by the ethics committee.
Funding: Refers to research funding. There are various sources of funding, such as through donations or by applying for various funding opportunities (called ‘grants’). Funding is highly competitive.
Academic paper: Is the most common form of reporting data, and is an important outcome for researchers in University-settings. The first person listed on the academic paper usually writes the paper from start to finish, with support from other authors. The last person is the most senior author.

## Slide 15
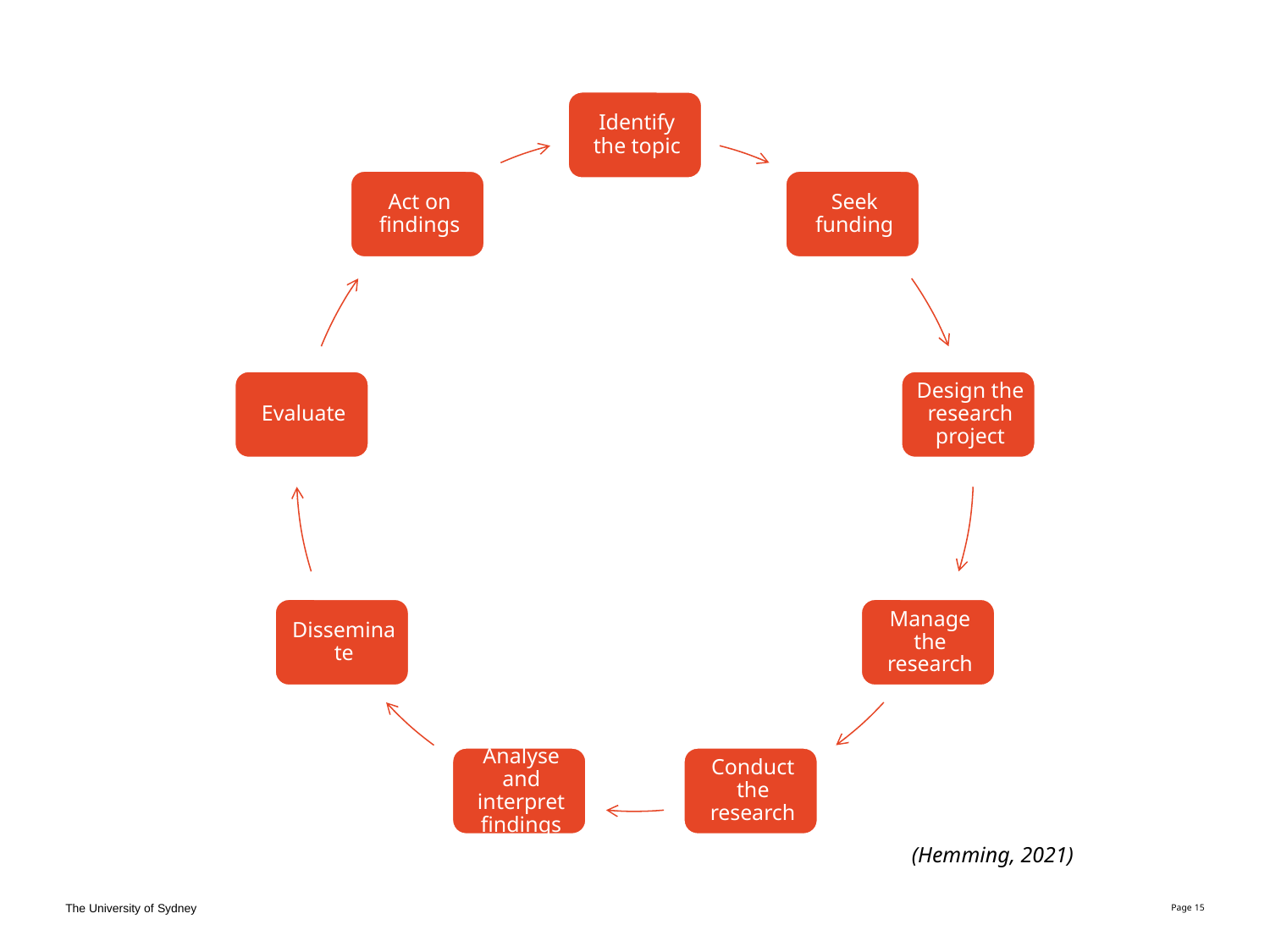

(Hemming, 2021)

## Slide 16
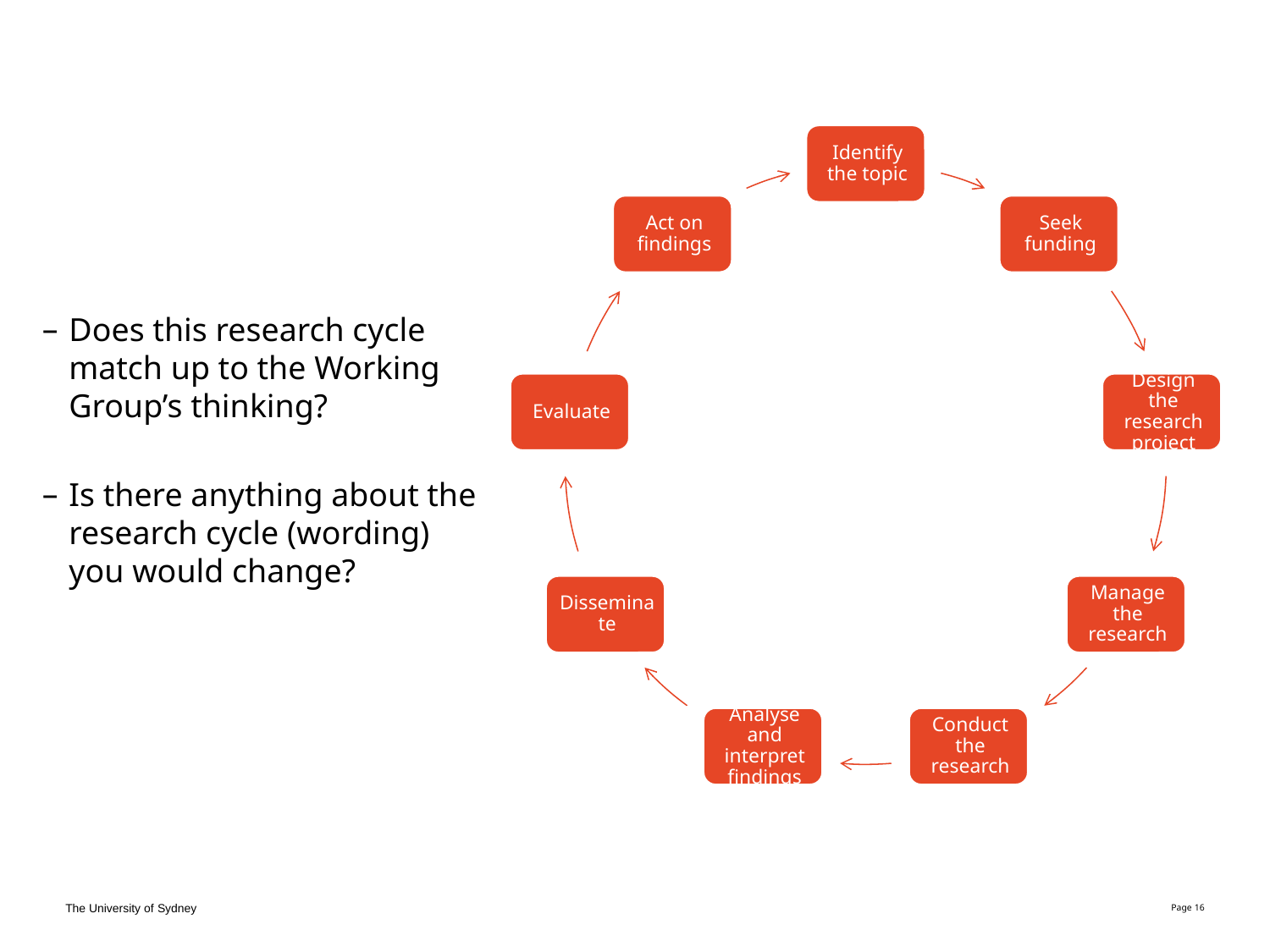

Does this research cycle match up to the Working Group’s thinking?
Is there anything about the research cycle (wording) you would change?

## Slide 17
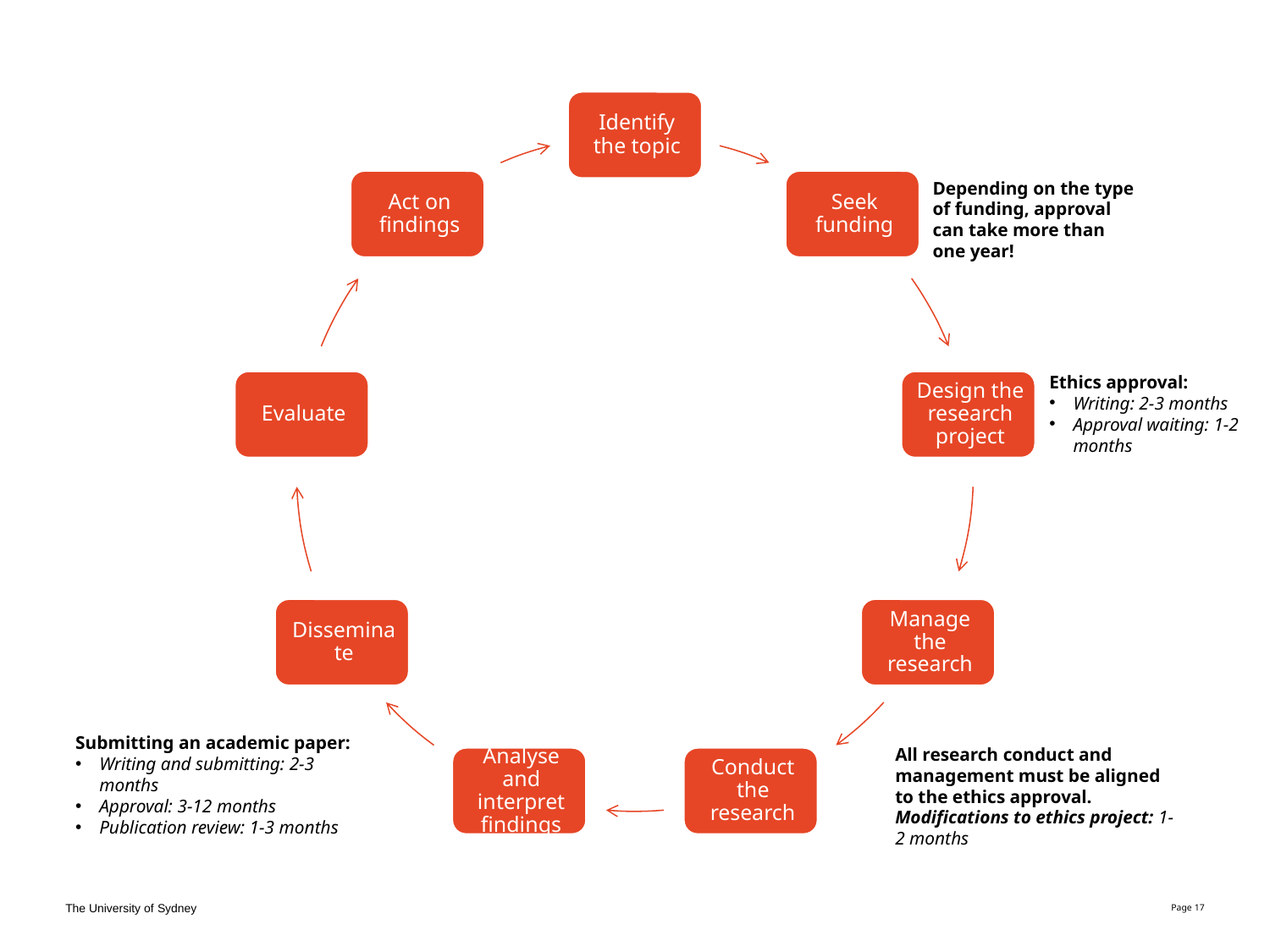

Depending on the type of funding, approval can take more than one year!
Ethics approval:
Writing: 2-3 months
Approval waiting: 1-2 months
Submitting an academic paper:
Writing and submitting: 2-3 months
Approval: 3-12 months
Publication review: 1-3 months
All research conduct and management must be aligned to the ethics approval.
Modifications to ethics project: 1-2 months

## Slide 18
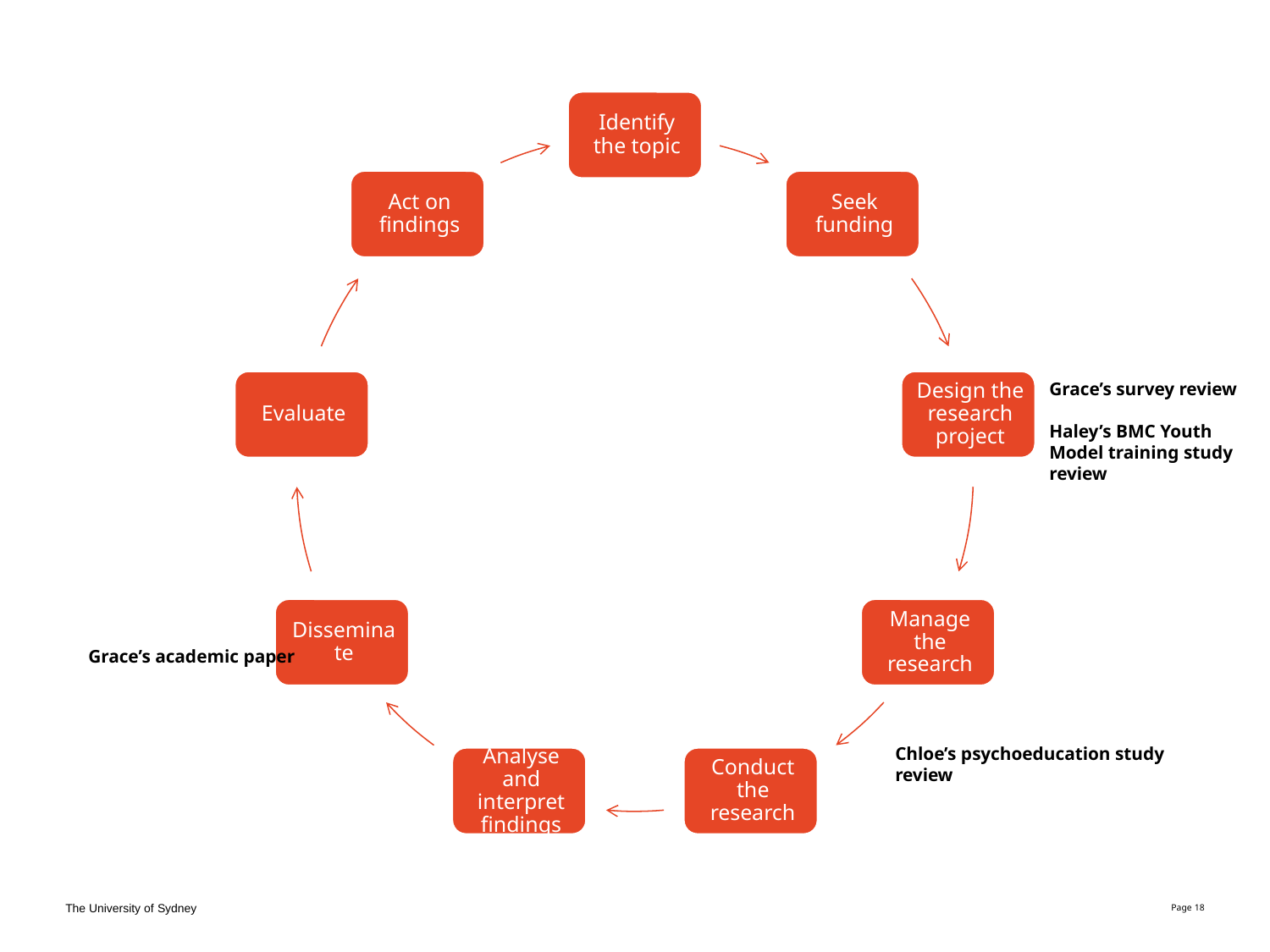

Grace’s survey review
Haley’s BMC Youth Model training study review
Grace’s academic paper
Chloe’s psychoeducation study review

## Slide 19
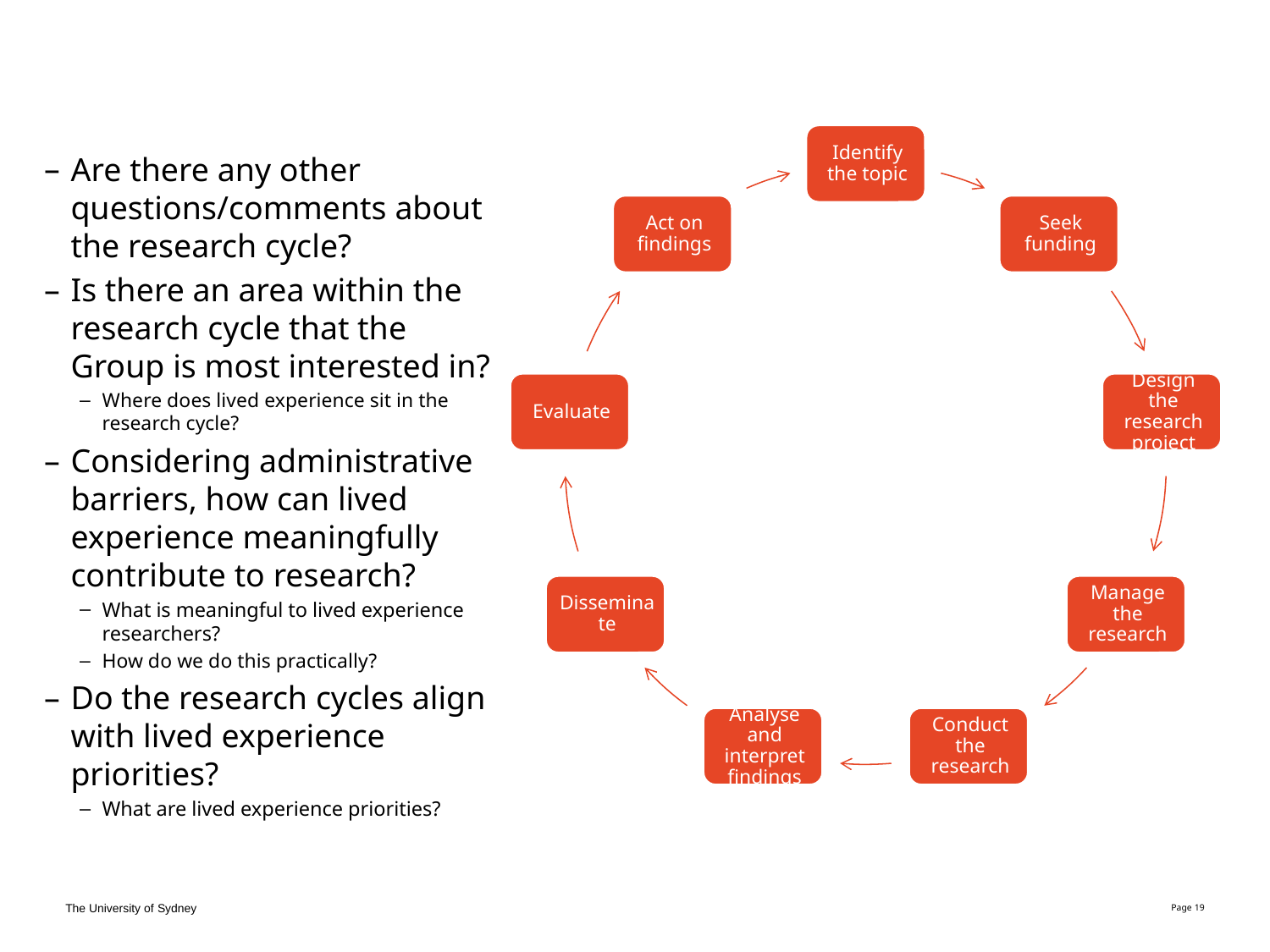

Are there any other questions/comments about the research cycle?
Is there an area within the research cycle that the Group is most interested in?
Where does lived experience sit in the research cycle?
Considering administrative barriers, how can lived experience meaningfully contribute to research?
What is meaningful to lived experience researchers?
How do we do this practically?
Do the research cycles align with lived experience priorities?
What are lived experience priorities?

## Slide 20
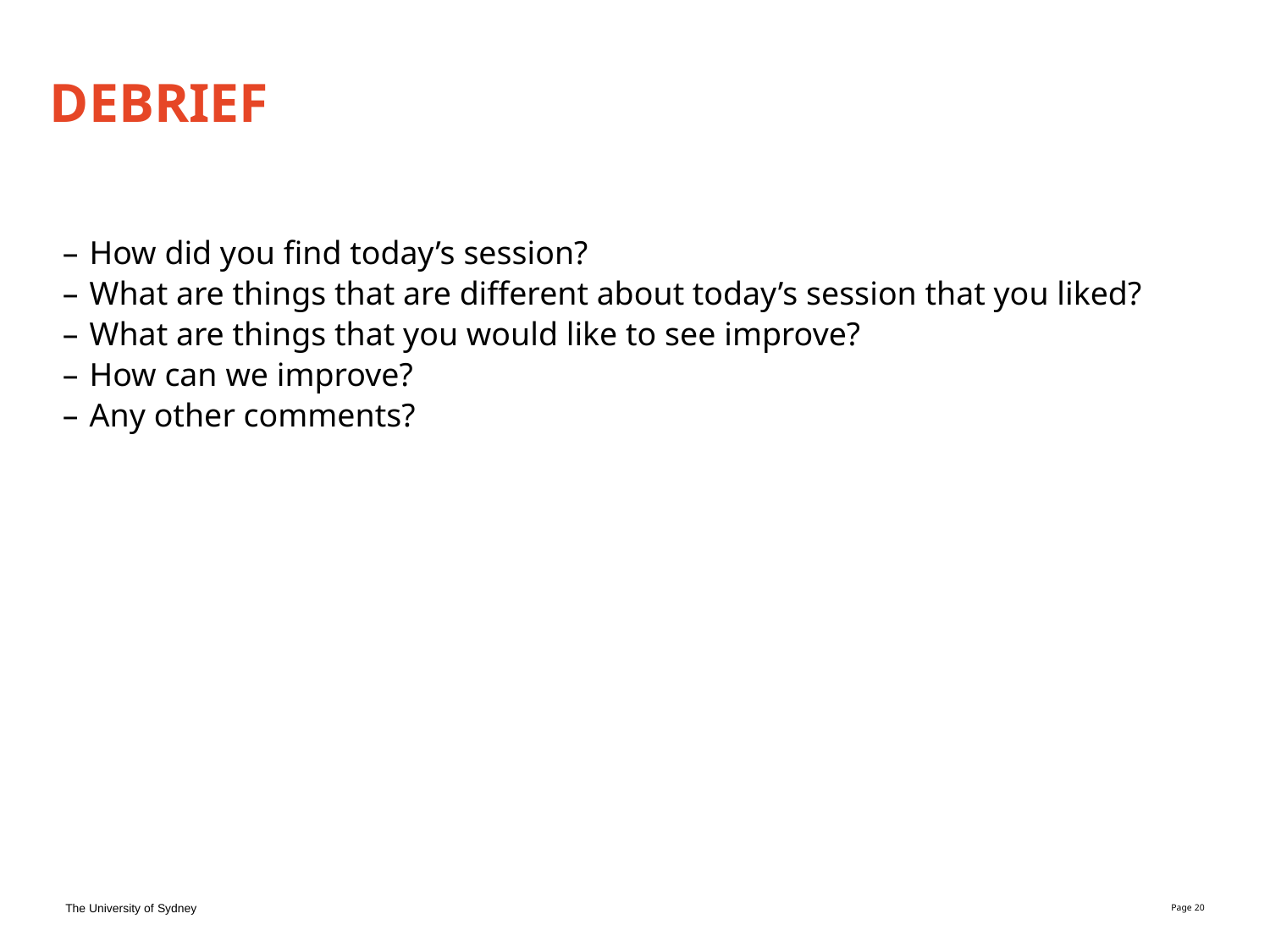

# DEBRIEF
How did you find today’s session?
What are things that are different about today’s session that you liked?
What are things that you would like to see improve?
How can we improve?
Any other comments?

## Slide 21
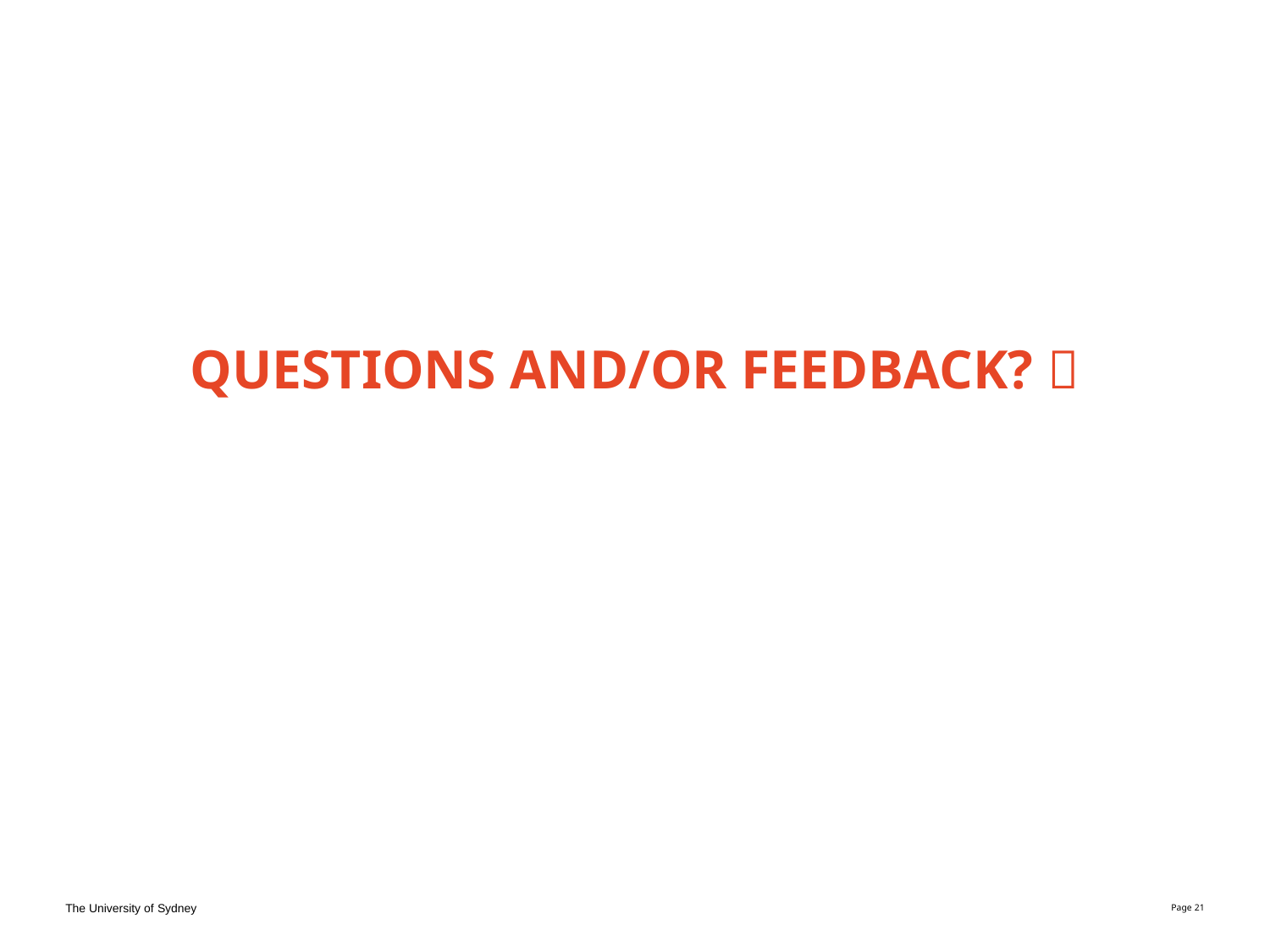

# QUESTIONS AND/OR FEEDBACK? 
